# Conformational entropy bottleneck in polymer nanopore interception driven by branching architecture and excluded volume

**DOI:** 10.1038/s41467-026-78055-1

**Published:** 2026-09-29

**Authors:** Yiren Wang, Haorong Huang, Jiawen Yang, Xin Guan, Mo Zhu, Mingming Ding, Lianwei Li

**Affiliations:** 1 College of Chemistry and Environmental Engineering, Shenzhen University, Shenzhen, China; 2 School of Chemical Engineering and Light Industry, Guangdong University of Technology, Guangzhou, China

**Keywords:** Dendrimers, Chemical physics, Polymers

## Abstract

The transport of polymers through nanopores is fundamentally governed by the interplay between molecular topology and nanoconfinement. However, how branching architecture influences the critical interception size (*R*_h,c_) under weak compression—where diffusion dominates and chain deformation is negligible—remains poorly understood. Here, we address this challenge by integrating well-defined hyperbranched polystyrenes with tunable branch densities (*ρ* = 1/25-1/650), monodisperse anodic aluminum oxide nanopores (*D* = 28-96 nm), all-atom simulations, and theoretical modeling to uncover a conformational entropy bottleneck mechanism. We experimentally reveal a distinct power-law relationship *R*_h,c_ ~ *ρ*^−1/2^ for hyperbranched polymers, in stark contrast to the classic behavior of linear chains. Moreover, hyperbranched architectures exhibit markedly weaker temperature dependence in interception behavior, attributed to enhanced intrachain excluded volume interactions. A combination of molecular dynamics simulation and theoretical analysis confirms that branching restricts conformational fluctuations, narrowing the distribution of accessible states and raising the free-energy barrier for pore entry. Our findings establish branching topology as a key regulator of polymer transport and interception in nanopores, offering new insights into the behavior of biopolymers like glycogen and starch in confined environments and guiding the design of advanced separation systems.

## Abstract (web summary)

The transport of polymers through nanopores is governed by the interplay between molecular topology and nanoconfinement. However, how branching architecture influences the critical interception size under weak compression remains poorly understood. Here, the authors use well-defined hyperbranched polystyrenes with tunable branch densities and monodisperse anodic aluminium oxide nanopores to uncover a conformational entropy bottleneck mechanism.

## Introduction

The transport and diffusion of macromolecules in nanoconfined porous media underpin key physicochemical processes in biological membranes (e.g., glomerular filtration^1^, RNA transport^2^, protein translocation^3,4^) and advanced separation technologies (e.g., gene sequencing^5^, nanosensing^6^, water treatment^7^). These phenomena critically rely on the interplay between molecular topology—ranging from linear and cyclic to globular and hyperbranched architectures—and nanoscale confinement. Based on local flow intensity, confined polymer transport bifurcates into two distinct regimes: the strong-compression limit, where high-velocity flow induces substantial chain deformation (e.g., coil-to-globule transition)^8^, and the weak-compression limit, governed by ultra-low flows where diffusion dominates^9^. Although theoretical frameworks, including confined blob models^10–12^, free energy analyses^13^, and mechanical descriptions^14^, have extensively elucidated strong-compression dynamics—characterized by entropic elasticity, viscous dissipation, and topological disentanglement—for diverse architectures such as linear, star, comb, hyperbranched polymers, and microgels^15–18^, the weak-compression limit remains poorly understood and a subject of ongoing debate.

The weak-compression limit, serving as the physical foundation of transport phenomena and being directly relevant to biological filtration, is characterized by near-equilibrium conditions, negligible deformation, diffusion-dominated dynamics, and a critical interception event when polymer dimensions approach the pore size^19^. The corresponding critical interception size (*R*_h,c_) represents an essential entropic bottleneck for molecular transport. Understanding the determinants of *R*_h,c_—particularly how molecular topology (especially branching) governs the critical conformational size under weak compression—constitutes a frontier challenge in soft matter science. Although previous studies under equilibrium conditions have established that the interception behavior of linear polymers is universal and independent of chemical composition^20^, these conclusions are largely based on architectures with linear connectivity (e.g., linear and star polymers), which fail to predict the behavior of highly branched systems like glycogen or starch^21,22^. This gap raises a fundamental question: Is *R*_h,c_ universal across polymers with vastly different topologies, such as linear and hyperbranched chains, in identical nanoconfined environments?

Indeed, recent studies on hyperbranched and other highly branched polymers have revealed that these systems exhibit complex multi-body hydrodynamic interactions, where the two-body approximation often fails. These interactions alter key scaling relationships—such as size-to-mass scaling and rheology—distinguishing them sharply from linear chains^23,24^. For instance, hyperbranched polystyrenes with uniform subchains show a unique double dependence in the Mark–Houwink–Sakurada relationship, i.e., [*η*]∼*M*_total_^0.39^*M*_sub_^0.31^, reflecting their compact architecture and reduced hydrodynamic volume^25^. In contrast, perfectly symmetric dendrimers lack fractal characteristics—a qualitative departure resulting from their ideal, defect-free, and overcrowded branching structure^26^.

We therefore hypothesize that hyperbranched polymers should exhibit distinct interception behavior from linear chains, namely, *R*_h,c_ may depend on the branching architecture. This distinction may originate from their highly branched topology, which promotes compact, globular conformations with restricted fluctuations, while simultaneously amplifying the excluded volume effect and enhancing chain rigidity. To address this hypothesis, it requires well-defined model systems: nanopores with controlled geometry, strictly regulated ultra-low flows, and topologically standardized polymers with precise branching parameters.

**Fig. 1:**
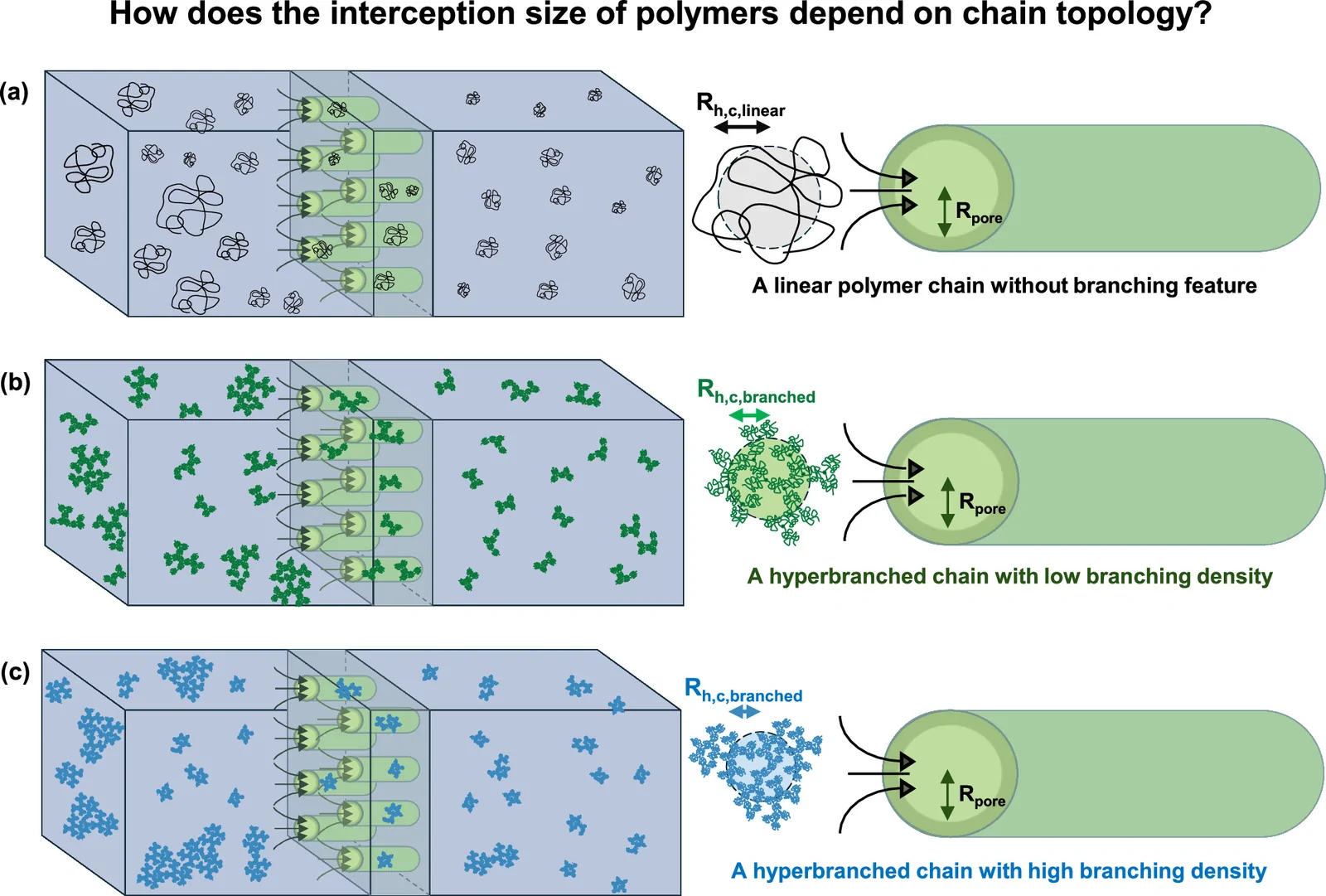
Topology-dependent polymer interception through cylindrical nanopores. **a**–**c** Schematics compare linear polystyrene (L-PS; black) with hyperbranched polystyrene (H-PS) of low (green) and high (blue) branching density. Polymers diffuse through anodic aluminum oxide nanopores under ultra-low flow and weak compression. Light-blue volumes represent the solution compartments, and green cylinders represent the nanopores. The right-hand diagrams compare the critical hydrodynamic interception radius, *R*_h,c_, with the pore radius, *R*_pore_. *R*_h,c,linear_ and *R*_h,c,branched_ denote the critical interception radii of linear and hyperbranched chains, respectively. Increasing branching density compacts H-PS and decreases the critical size required for interception.

To test our hypothesis (Fig. 1), we employed a model-decoupling strategy combining custom anodic aluminum oxide (AAO) nanopore arrays with controlled geometry and well-defined hyperbranched polystyrene (H-PS) samples of tuned branching density (*ρ*), alongside linear PS (L-PS), under strictly controlled ultra-low flows. Key results reveal: (1) For pores (28–96 nm), increasing branching densities (*ρ*) from 1.54 × 10^−3^ to 4.00 × 10^−2^ reduces *R*_h,c_ with scaling *R*_h,c_ ~ *ρ*^−1/2^, significantly below L-PS values; (2) Theoretical analysis and all-atom simulation confirm that branching governs conformational fluctuations, free energy barriers, and translocation probability; (3) While the increase in temperature expands the chain dimensions, *R*_h,c_ remains nearly constant for hyperbranched architectures—unlike the strong temperature dependence in linear chains—demonstrating topology-dominated exclusion effects under weak compression.

## Results

### Sample preparation and structural characterization

**Fig. 2:**
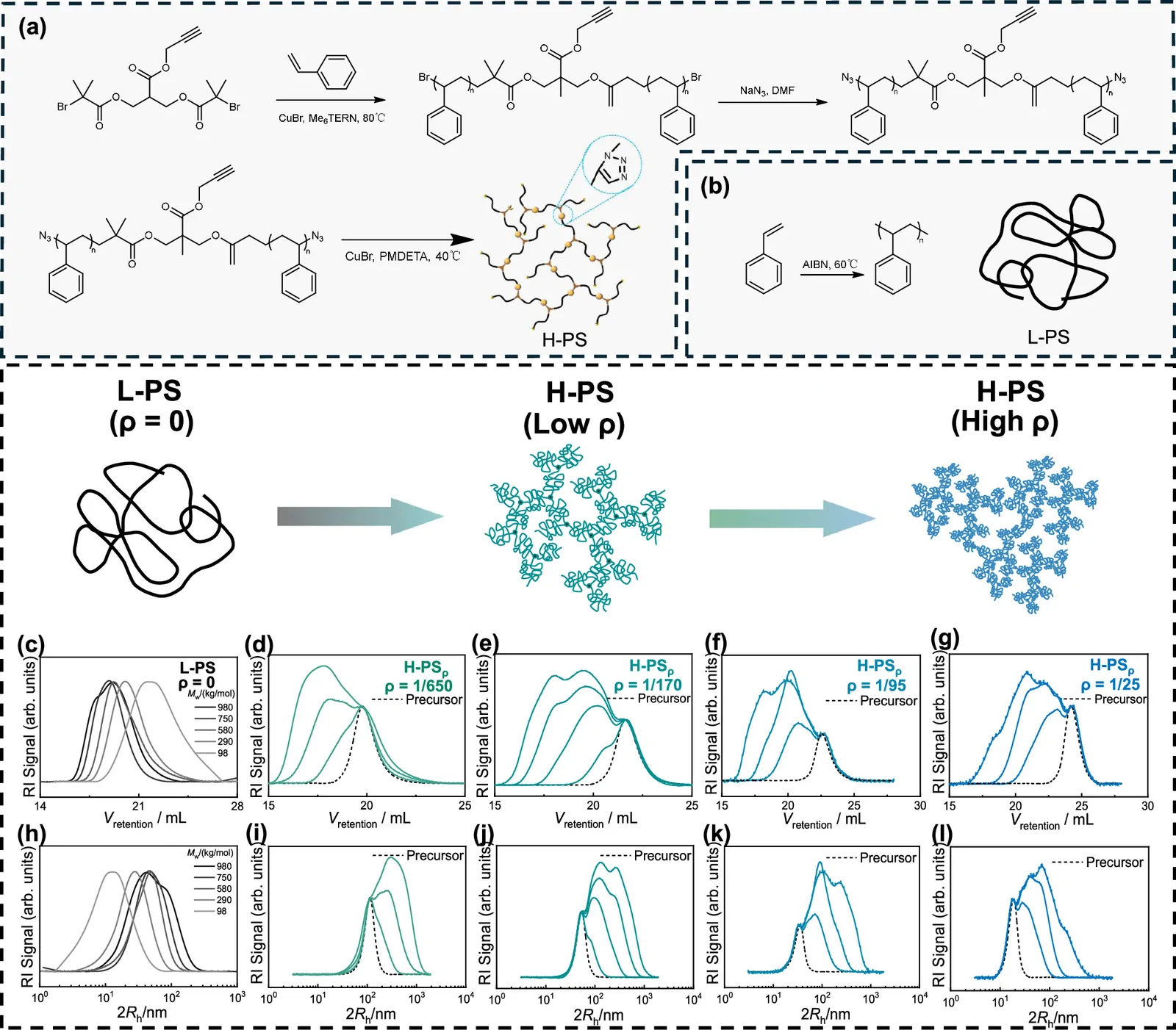
Synthesis and hydrodynamic size characterization of architecturally defined polystyrenes. **a** Hyperbranched polystyrene (H-PS) was prepared by atom-transfer radical polymerization (ATRP), azide substitution, and copper-catalyzed azide–alkyne cycloaddition (CuAAC). **b** Linear polystyrene (L-PS) was prepared by azobisisobutyronitrile (AIBN)-initiated free-radical polymerization. **c**–**g** Size-exclusion chromatography (SEC) traces of L-PS and H-PS with branching densities *ρ* = 1/650, 1/170, 1/95, and 1/25. Black-to-gray traces in (**c**) distinguish the listed weight-average molecular masses (*M*_w_). Green-to-blue traces identify H-PS samples with increasing *ρ*, as labeled. Black dotted traces are the corresponding macromonomer precursors. **h**–**l** Hydrodynamic-diameter (2*R*_h_) distributions obtained from SEC calibration; colors and dotted precursor traces follow (**c**–**g**). The schematics shown alongside the data illustrate the architectural progression from L-PS to H-PS with increasing branching density. The refractive-index (RI) signal is shown in arbitrary units (arb. units), and *V*_retention_ is retention volume. SEC used tetrahydrofuran as the mobile phase at 1.0 mL min^−1^.

Figure 2a, b outlines the synthetic routes for model H-PS and L-PS samples. H-PS architectures were synthesized via a group-developed strategy combining atom transfer radical polymerization (ATRP) and copper-catalyzed azide–alkyne cycloaddition (CuAAC) click chemistry^27–29^. Using well-characterized AB_2_-type macromonomers with degrees of polymerization (DP) ranging from 50 to 1300 (Supplementary Figs. 1–3), we obtained hyperbranched structures possessing branch lengths of 25–650 styrene units, corresponding to *ρ* from 1/25 to 1/650. For each macromonomer, a series of H-PS samples (denoted H-PS_ρ_) were prepared, covering an apparent weight-average molecular weight (*M*_w,app_) range of 0.19 × 10^5^−5.13 × 10^5 ^Da and polydispersity indices (*Đ*) of 1.51–6.89 (Supplementary Table 1). The subchain segments of H-PS_ρ_ samples exhibit flexible characteristics, given that the Kuhn length of PS is approximately 1.5 nm (≈7 styrene units)^30,31^. In parallel, L-PS samples were prepared via conventional azobisisobutyronitrile (AIBN)-based free-radical polymerization, yielding materials with *M*_w,app_ = 0.96 × 10^5^−9.8 × 10^5 ^Da and *Đ* = 2.12–2.89 (Supplementary Table 2).

Unlike previous studies that relied on sporadic, discrete samples to estimate pore-passage probability or identify a critical interception size, such a sparse sampling strategy inherently increases experimental uncertainty, making it difficult to precisely pinpoint the true critical size for diffusion through a pore^32–34^. In contrast, our use of broadly distributed samples enables a continuous assessment of interception probability as a function of chain length or molecular weight, thereby avoiding the ambiguities associated with selectively chosen discrete data points. Although size-exclusion chromatography (SEC) provides only an apparent molecular weight for non-linear architectures, it affords reliable hydrodynamic size estimations. Grounded in the universal principle of size exclusion, SEC served as the most suitable method for resolving the continuous, broad molecular weight distributions essential for this study.

The raw SEC chromatograms (Fig. 2c–g) were calibrated using linear polystyrene standards to establish a definitive *M* versus *V*_retention_ relationship (Supplementary Fig. 4). Applying universal scaling laws for hydrodynamic radius *R*_h_ and gyration radius *R*_g_ versus *M* (Supplementary Fig. 5), we transformed these into continuous profiles of 2*R*_h_ (Fig. 2h–l) and *R*_g,app_ (Supplementary Fig. 6). It is well-established by rigorous theoretical calculations^35,36^ that SEC separation is universally governed by hydrodynamic volume (or *R*_h_), rather than *R*_g_, irrespective of chain topology. Extensive experimental evidence on diverse branched architectures further confirms that *R*_h_ remains a robust, topology-independent metric for SEC calibration, as branching primarily compresses the distal segments without dramatically altering the hydrodynamic core^37,38^. Therefore, deriving the *R*_h_ profiles for our hyperbranched variants using linear polystyrene standards is physically justified and circumvents systematic topology-induced bias (Supplementary Fig. 7). In this context, *R*_h_ closely approximates the true hydrodynamic radius for hyperbranched samples, whereas *R*_g,app_, derived from linear chains, may deviate from actual *R*_g_ due to architectural differences and serves merely as a structural reference. To experimentally verify that topological differences do not introduce systematic bias in our *R*_h_ determination, preparative SEC fractionation and offline dynamic light scattering (DLS) were performed on co-eluting matrices (Supplementary Figs. 8 and 9), confirming precise physical size equivalence. Quantitatively, the hydrodynamic diameters (2*R*_h_) for samples (L-PS, H-PS_1/650_, H-PS_1/170_, H-PS_1/95_, H-PS_1/25_) span ~10 to ~100 nm, with some extending sub-10 nm or exceeding 100 nm, providing a solid foundation for systematically probing interception behavior across pore sizes.

### Nanopore uniformity and interception protocol validation

**Fig. 3:**
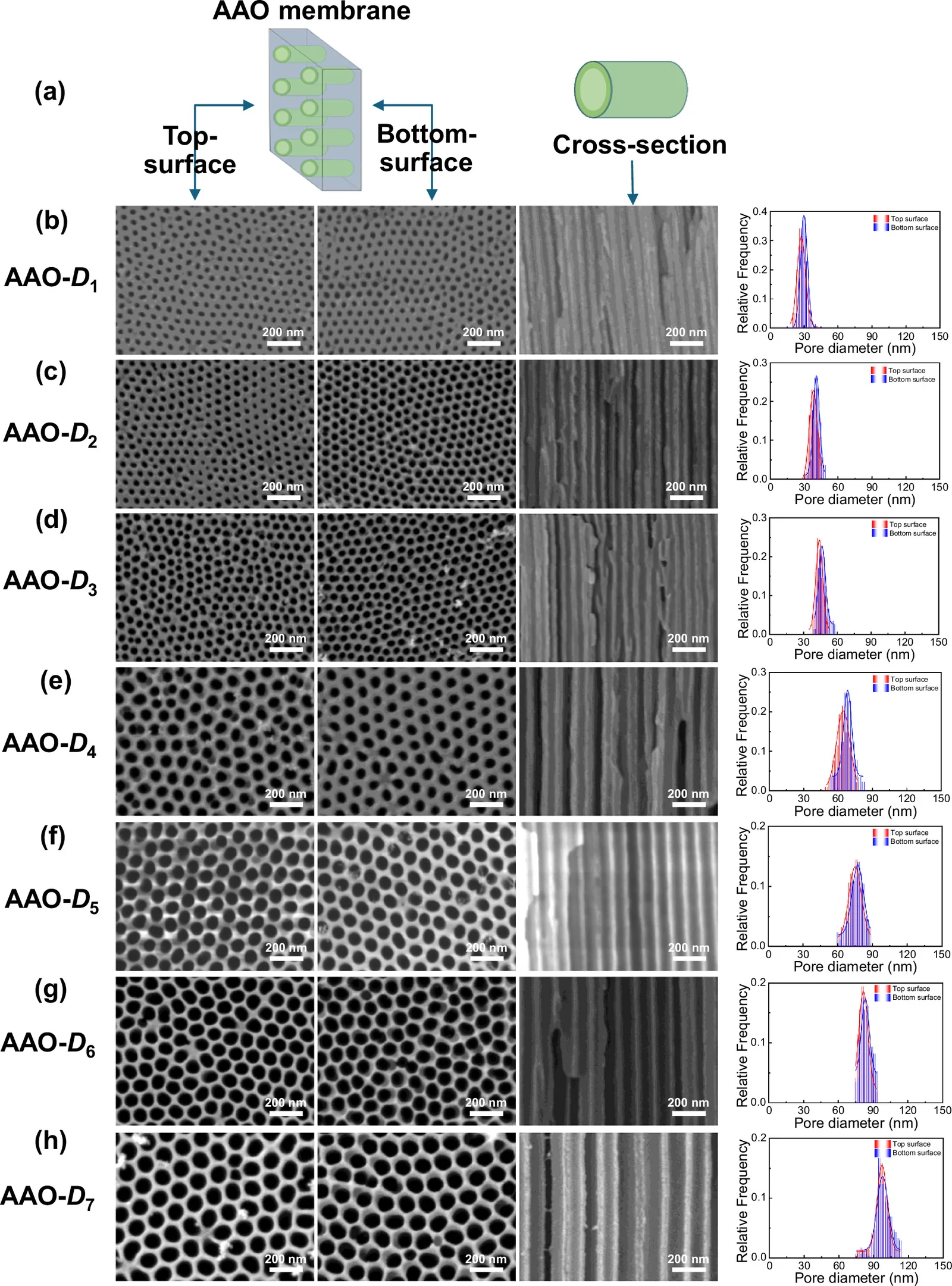
Structural uniformity of anodic aluminum oxide nanopores. **a** Schematic showing the top surface, bottom surface, and cross-section of an anodic aluminum oxide (AAO) membrane. **b**–**h** Scanning electron microscopy (SEM) images and pore-diameter distributions for AAO-*D*_1_ to AAO-*D*_7_. In each row, the first, second, and third images show the top surface, bottom surface, and cross-section, respectively. Red and blue histograms and fitted curves represent the top- and bottom-surface pore-size distributions, respectively. Mean pore diameters span ~28–96 nm, and the dispersity (*Đ*) is below 1.02. All SEM scale bars, 200 nm.

The availability of well-defined nanoporous membranes is crucial for reliable interception studies. Based on the previous work^39,40^, we fabricated a batch of anodic aluminum oxide (AAO-*D*_n_) nanochannel membranes. These membranes exhibit average pore diameters (*D*) ranging from ~28 to ~96 nm (AAO-*D*_1_ to AAO-*D*_7_), a consistent channel length of ~50 μm, and a pore density of ~6.2 × 10^9^ to 2.7 × 10^10^ pores/cm^2^ (Supplementary Table 3), providing the basis for estimating single-pore diffusion and microscopic flow rates. Systematic structural characterization of the top-surface, bottom-surface, and cross-section of AAO-*D*_n_ membranes was performed to evaluate their pore size (Fig. 3a). Statistical analysis of the pore size distributions (Fig. 3b–h) revealed nearly circular pores on both top and bottom surfaces, with mean diameters differing by <2.0 nm and a narrow polydispersity index (*Đ* < 1.02). Cross-sectional imaging further verified their well-defined, parallel cylindrical morphology, establishing the AAO-*D*_n_ series as a quasi-ideal model porous medium for interception experiments.

In addition to scanning electron microscopy (SEM) characterization, we employed a transmembrane pressure method to assess the average pore size of the AAO-*D*_n_ membranes (Supplementary Fig. 10). Sequential filtration experiments were performed using pure solvent followed by PS samples of varying topologies (L-PS and H-PS with different *ρ*). Filtrates from each interception step were analyzed by SEC to determine the critical hydrodynamic interception size.

**Fig. 4:**
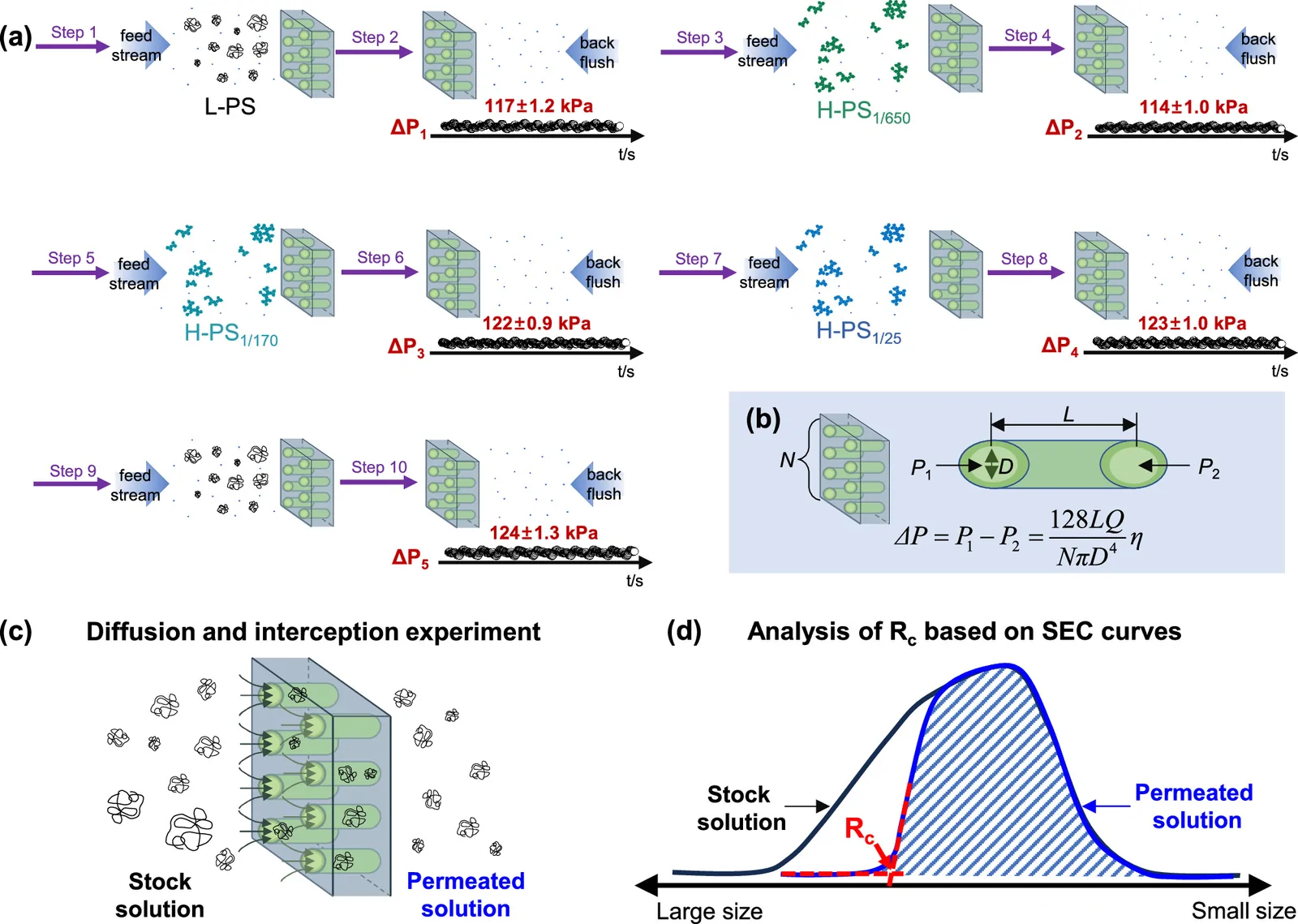
Sequential interception protocol and determination of the critical retention size. **a** Sequential measurements with linear polystyrene (L-PS), hyperbranched polystyrene (H-PS)_1/650_, H-PS_1/170_, H-PS_1/25_ and L-PS, separated by reverse-solvent flushing. Purple arrows indicate the experimental sequence, and blue arrows indicate feed or back-flush direction. Black markers show transmembrane pressure (Δ*P*) versus time (*t*); red annotations report values and their stated uncertainties, which remain within 114−124 kPa. H-PS subscripts denote branching density. **b** Poiseuille description of flow through *N* pores. *P*_1_ and *P*_2_ are upstream and downstream pressures; *D*, *L*, *η*, and *Q* denote pore diameter, channel length, solvent viscosity, and volumetric flow rate, respectively. **c** Diffusion and interception of polymers from the stock solution into the permeate. **d** Determination of the critical retention size (*R*_c_) from size-exclusion chromatography (SEC) curves. Black and blue curves denote stock and permeate distributions; blue hatching marks the size range represented in the permeate. Red dashed guides, and the red arrow locates *R*_c_.

Figure 4a depicts a sequential interception experiment conducted in the order: linear L-PS → low-branching-density H-PS → medium-branching-density H-PS → high-branching-density H-PS → linear L-PS. Following each interception, a reverse solvent flush was employed to mitigate concentration polarization and membrane contamination, while the transmembrane pressure difference (Δ*P*) was monitored at each step. Initial Δ*P* after L-PS interception using a *D* = 32 nm AAO membrane was 117 kPa, with subsequent values of 114, 122, 123, and 124 kPa. The minimal pressure variation indicates membrane stability, corroborated by similar results for a *D* = 72 nm membrane (Supplementary Fig. 11).

The transmembrane pressure (Δ*P*) correlates with pore diameter (*D*), channel length (*L*), pore density (*n*), solvent viscosity (*η*), and flow rate (*Q*) via the Poiseuille equation (Fig. 4b). Since Δ*P* ~ 1/*D*^4^, the slight increase from 117 to 124 kPa corresponds to a negligible pore diameter change (~1.5%), confirming the structural integrity and uniformity of the AAO membranes. Control experiments further validated the Poiseuille equation’s applicability, showing linear relationships for both variable pore diameters (fixed solvent) and variable solvents (fixed pore), as summarized in Supplementary Fig. 12. Excellent agreement between pore sizes derived from the Poiseuille equation and SEM analysis (Supplementary Table 3) reinforces the robustness of this hydrodynamic approach for assessing membrane permeability and pore stability.

Interception experiments were conducted at *Q* = 0.2 mL/h with temperature maintained at 25 ± 1°C and polymer concentration below 1.00 g/L. Approximately 10 mL of pre-filtered polymer solution was injected into the syringe. As schematically illustrated in Fig. 4c, the interception experiment continuously drives the dilute polymer feed stream through the AAO membrane. Subsequently, by quantitatively comparing the molecular information—specifically the size-exclusion chromatograms—of the original feed solution and the collected permeate, the specific retention size for each topology can be precisely determined (Fig. 4d). Quantitatively, the microscopic flow rate (*q*) for each single pore was calculated to be *q* = 2.06 × 10^−21^ to 8.97 × 10^−21^ m^3^/s based on the pore density data (6.20 × 10^9^ to 2.71 × 10^10^ pores/cm^2^), corresponding to microscopic flow velocity of ~1.24 × 10^−6^ to 3.35 × 10^−6 ^m/s (Supplementary Table 3), which is 10–10^2^ times smaller than that of solvent molecules. The provided flow disturbance is much smaller than the fluctuation of solvent motion excited by thermal energy, which can ensure that the designed system belongs to the weak compression limit. In addition, the cumulative hydrodynamic shear energy acting on a molecule (with an estimated contour length of 50–1000 nm) is ~1.9 × 10^−6^ to 7.9 × 10^−6^ *k*_B_*T*, significantly lower than the thermal energy *k*_B_*T*.

### Branching-dependent interception behavior

**Fig. 5:**
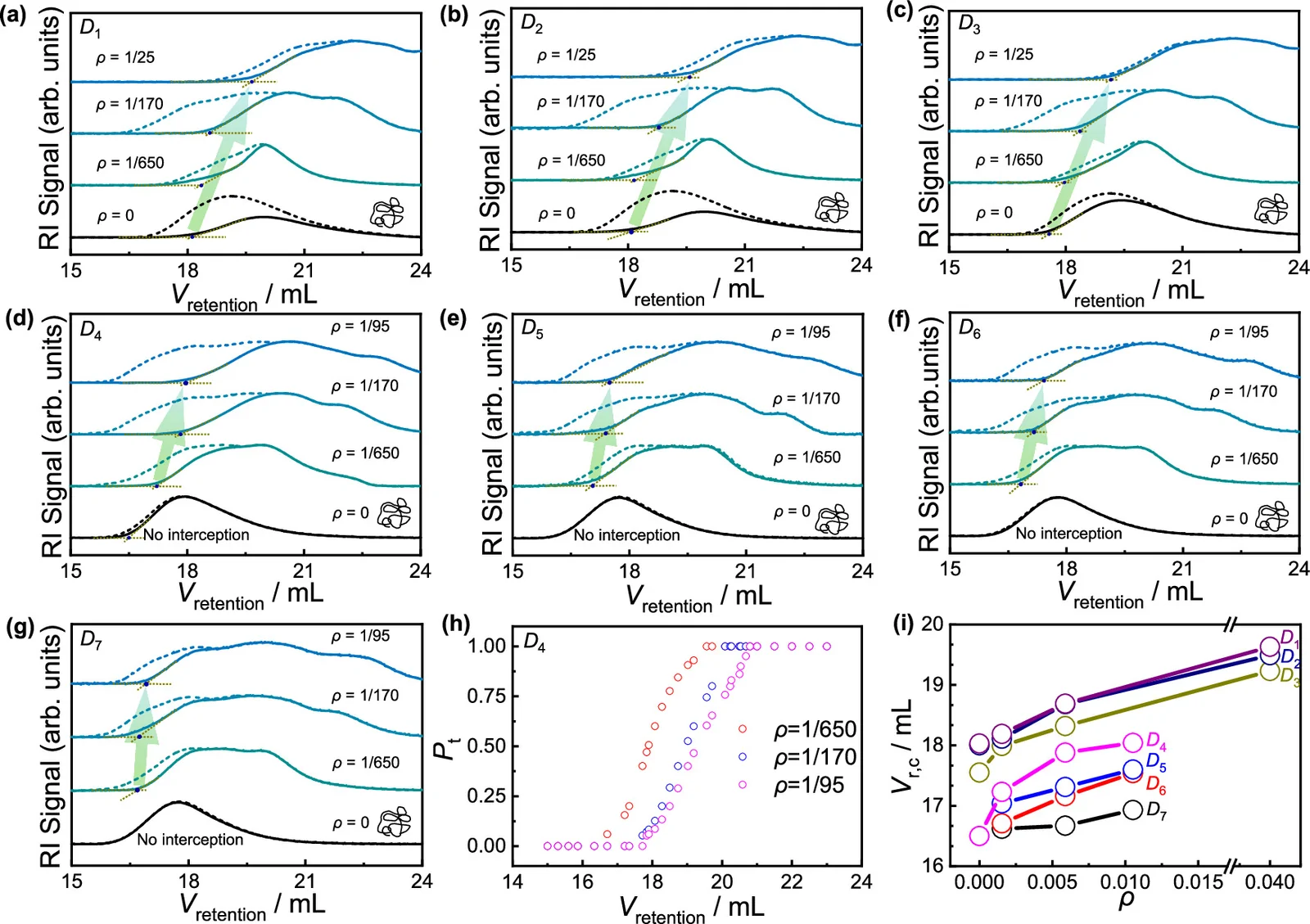
Branching-dependent interception across nanopores of different diameters. **a**–**g** Size-exclusion chromatography (SEC) traces for linear polystyrene (L-PS; *ρ* = 0) and hyperbranched polystyrene (H-PS) with the labeled branching densities *ρ*. Measurements used anodic aluminum oxide (AAO) membranes *D*_1_ to *D*_7_ (28–96 nm). Dotted and solid curves denote stock and permeate solutions, respectively. Curve colors distinguish *ρ* values as labeled. Translucent green arrows trace the branching-dependent shift in transmission onset, and yellow dotted guides mark the corresponding critical retention volumes. “No interception” indicates overlapping stock and permeate distributions. **h** Translocation probability (*P*_t_) versus retention volume (*V*_retention_) for AAO-*D*_4_; open-circle colors identify *ρ* values as labeled. **i** Critical retention volume (*V*_r,c_) versus *ρ*; colored open circles and connecting lines identify *D*_1_–*D*_7_ as labeled in the panel. Experiments used *T* = 25 °C, *Q* = 0.20 mL h^−1^. SEC used tetrahydrofuran as the mobile phase at 1.0 mL min^−1^.

It is worth noting that for the four samples to be intercepted, we all followed the principle of selecting samples from the sample library that are slightly larger in size than the pore diameter, in order to reduce the concentration difference and polarization effects. Taking the hyperbranched sample with *ρ* = 1/650 as an example, since the pore diameters of *D*_1_-*D*_3_ are small, we chose the sample with a smaller molecular weight (Supplementary Table 1, entry 11), while the corresponding *D*_4_-*D*_7_ were selected from samples with a larger molecular weight (Supplementary Table 1, entry 12). Figure 5a–g presents the raw SEC data from sequential interception experiments for linear and hyperbranched polymers across nanopores with diameters ranging from 28 to 96 nm. Consistently, branching-dependent interception curves were observed across all pore sizes. For example, in the case of pore *D*_6_ (88 nm), the critical retention volume (*V*_r,c_) increased sequentially with increasing branching density: *V*_r,c_ values for H-PS_1/650_, H-PS_1/170_, and H-PS_1/95_ were measured as 16.82 mL, 17.16 mL, and 17.42 mL, respectively.

Linear PS exhibited distinct pore-size-dependent interception: no significant interception was detected for pores larger than 55 nm (e.g., *D*_4_), indicating that even the largest chains in the broad-distribution sample could pass through. In contrast, for pores smaller than 55 nm, a clear critical retention volume emerged. This finding is intriguing given that SEC analysis indicated a maximum hydrodynamic diameter of ~100 nm for L-PS, suggesting a discrepancy that can be rationalized by the inherent band-broadening effects in SEC, which distort size estimation for species at the distribution extremes. For hyperbranched PS, although some samples had sizes comparable to the pore dimensions, extensive statistical analysis unequivocally demonstrated that the interception size is primarily governed by branching density, with higher density leading to a smaller critical interception size.

Beyond the analysis of critical interception sizes (Fig. 5a–g), the translocation probability as a function of retention volume, *P*_t_(*V*_r_), offers a comprehensive perspective over the full chain-length distribution. Figure 5h shows the pore-passage probability versus retention volume for the three hyperbranched samples tested with the *D*_4_ pore, demonstrating a gradual transition from 100% interception at large sizes to 100% diffusion passage at small sizes. *P*_t_(*V*_r_) curves for other pore sizes are provided in Supplementary Fig. 13. From these curves, we extracted the critical retention volume, *V*_r,c_, for each polymer–pore combination. Across all pore sizes, *V*_r,c_ shifted systematically to larger values as the branching density, *ρ*, increased (Fig. 5i). Because SEC retention volume increases as hydrodynamic size decreases, this trend indicates that more densely branched polymers were intercepted at smaller hydrodynamic sizes. At a fixed *ρ*, *V*_r,c_ decreased with increasing pore diameter, consistent with the larger critical size required for interception by wider pores.

The critical interception size, *R*_h,c_, for each polymer–pore combination was objectively determined from these *P*_t_(*V*_r_) curves. Taking into account the broadening effect of the SEC curve, we define *R*_h,c_ as the hydrodynamic radius at which the interception starts. The critical *V*_r,c_ can be converted to *R*_h,c_ using the well-established SEC calibration relationship between retention volume and hydrodynamic radius (Supplementary Figs. 4 and 5). The use of broadly distributed polymer samples was crucial here, as it enabled a continuous mapping of *P*_t_ versus *R*_h_, thereby avoiding the ambiguities associated with interpreting data from only a few discrete molecular weights. Representative examples illustrating the determination of *R*_h,c_ from the interception probability curves are provided in Supplementary Fig. 14.

A key observation is that linear chains display a broader transition interval in retention volume—from full interception to full passage—than hyperbranched chains. This wider transition window, corresponding to an extended size range, is intrinsically associated with the broader conformational fluctuations typical of linear chains. These results clearly demonstrate that branching density systematically shifts the interception curves, suggesting a topology-dependent critical size. To quantify this dependence, we next performed a scaling analysis to extract *R*_h,c_ as a function of *ρ*.

### Scaling analysis of critical interception size

**Fig. 6:**
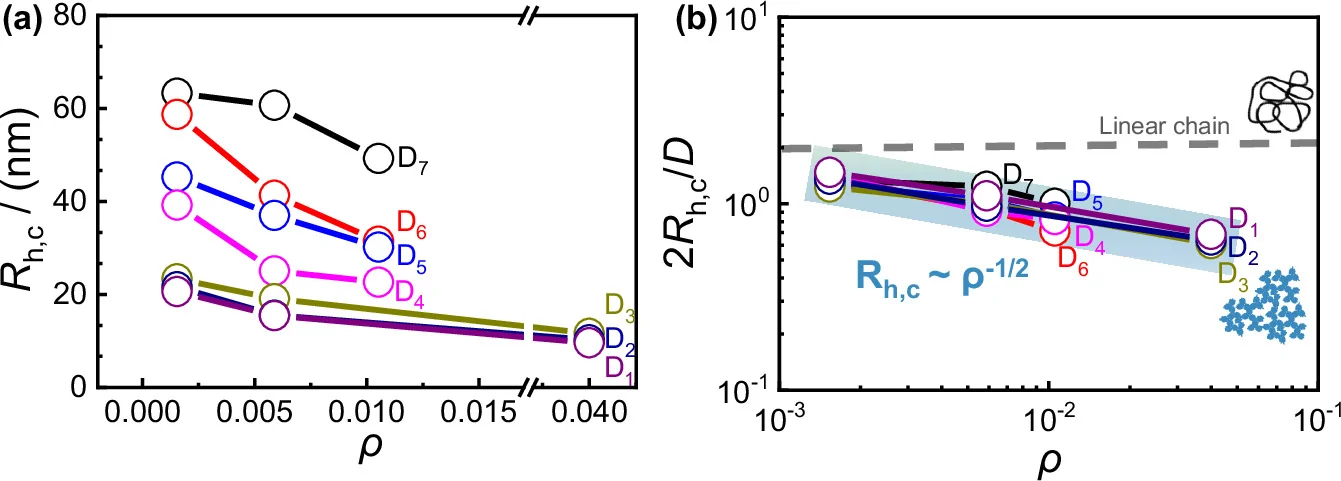
Critical hydrodynamic interception radius scales with branching density. **a** Critical hydrodynamic interception radius (*R*_h,c_) versus branching density (*ρ*) for anodic aluminum oxide (AAO) nanopores *D*_1_ to *D*_7_. Colored open circles and connecting lines identify the pore series as labeled. **b** Normalized interception diameter, 2*R*_h,c_/*D*, versus *ρ* on logarithmic axes, where *D* is pore diameter. The colored curves retain the *D*_1_–*D*_7_ assignments from **a**. The gray dashed line marks the linear-chain reference. The light-blue shaded region describes the topology-dependent scaling *R*_h,c_ ∼ *ρ*^−1/2^. Polymer cartoons indicate the linear-chain and compact hyperbranched limits.

Building on the interception profiles presented in Fig. 5, we now systematically extract the critical hydrodynamic interception size *R*_h,c_ for each branching density and pore size. Figure 6a presents absolute *R*_h,c_ values, while Fig. 6b shows the pore-diameter-normalized quantity 2*R*_h,c_/D. As shown in Fig. 6a, a universal trend emerges across all pore sizes (*D*_1_-*D*_7_): *R*_h,c_ decreases systematically with increasing *ρ*. For example, with pore *D*_3_ (41 nm), *R*_h,c_ drops from ~27 nm for linear PS (*ρ* = 0) to ~15 nm for highly branched H-PS_1/25_ (*ρ* ≈ 0.04), confirming the strong dependence of interception size on molecular topology. The normalized analysis in Fig. 6b offers deeper physical insight. The double-logarithmic plot can be roughly described by the blue band with a power-law scaling of 2*R*_h,c_/*D* ~ *ρ*^−1/2^, which starkly contrasts with the behavior of linear chains and points to a distinct entropic bottleneck mechanism. Strikingly, the experimental data exhibit an approximate empirical scaling behavior, 2*R*_h,c_/*D* ~ *ρ*^−1/2^. From a physical perspective, since Flory theory predicts *R*_g_ ~ *ρ*^−1/4^ for ideal hyperbranched chains, the observed scaling implies *R*_h,c_ ~ *R*_g_^2^. This intriguing relationship suggests that the nanopore interception is dominantly governed by the two-dimensional projected area of the dense topological core, rather than its simple linear dimension. This scaling analysis reveals for the first time how molecular topology governs interception under nanoconfinement—specifically, increased branching density yields more compact architectures, reducing the effective size required for nanopore passage. We further hypothesize that the dependence of 2*R*_h,c_/*D* ~ *ρ*^−1/2^ should be attributed to the difference in conformational distribution and the free energy barrier for pore translocation relative to linear chains.

### Temperature dependence of interception behavior: hyperbranched vs. linear

The transport and interception of biopolymers such as starch and glycogen in porous media are often influenced by changes in external conditions. Therefore, we further examined whether the interception behavior of branched and linear polymers exhibits significant temperature dependence. Cyclohexane, a typical theta solvent for PS, was selected because PS chains display pronounced temperature-dependent conformational changes in this solvent. Near the theta temperature (*T*_θ_ = 34.5 °C), the excluded volume parameter *v* changes sign, allowing us to decouple topological effects from solvent-mediated interactions. Below *T*_θ_, chains collapse and excluded volume is negative; above *T*_θ_, chains swell with positive excluded volume. This solvent system thus provides a sensitive means to assess whether interception is governed primarily by chain size (temperature-dependent) or by branching topology (temperature-invariant).

**Fig. 7:**
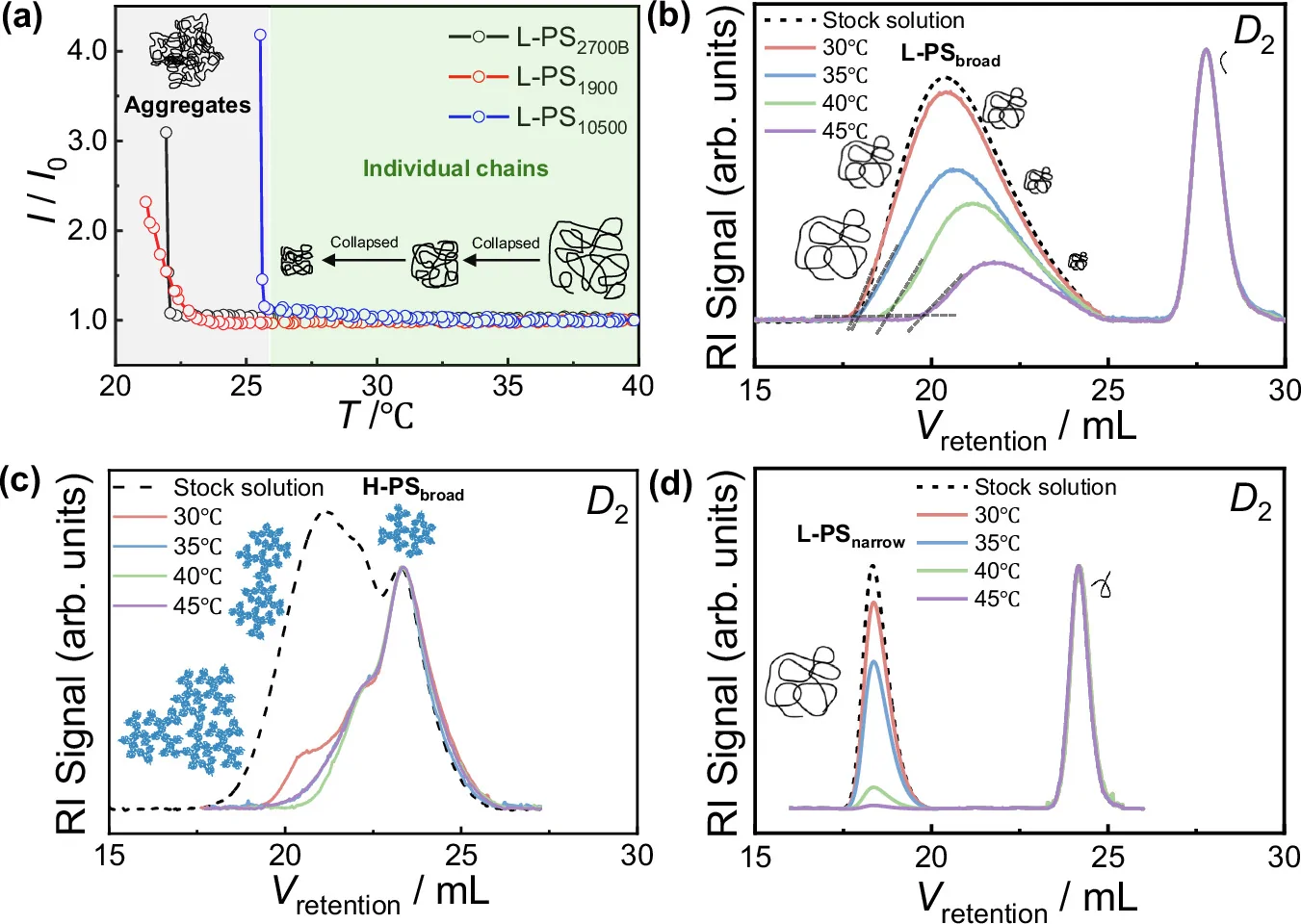
Temperature dependence of linear- and hyperbranched-polystyrene interception. **a** Relative static-light-scattering intensity (*I*/*I*_0_) versus temperature (*T*) for L-PS_2700B_ (black), L-PS_1900_ (red), and L-PS_10500_ (blue) in cyclohexane. The low-temperature unshaded region denotes aggregates, and light-green shading denotes dispersed individual chains; cartoons illustrate chain collapse with decreasing temperature. **b**–**d** Size-exclusion chromatography (SEC) profiles after interception through an anodic aluminum oxide membrane (AAO-*D*_2_) for broadly dispersed L-PS, broadly dispersed H-PS, and narrowly dispersed L-PS, respectively. Dashed black curves denote stock solutions. Red, blue, green, and purple solid curves denote permeates collected at 30, 35, 40, and 45 °C, respectively. L-PS and H-PS are linear and hyperbranched polystyrene; “broad” and “narrow” denote broad and narrow molecular-weight distributions. Interception was performed in cyclohexane; SEC used tetrahydrofuran as the mobile phase at 1.0 mL min^−1^.

First, static light scattering was used to measure the temperature dependence of scattering intensity (Fig. 7a). For both narrowly distributed (L-PS_1900_, L-PS_10500_) and broadly distributed (L-PS_2700B_) samples, significant intermolecular aggregation was observed in the 20–30 °C range, reflected by a increase in scattering intensity with decreasing temperature. Above *T* = 30 °C, the chains remained well-dispersed in a collapsed single-chain state. Figure 7b, c further illustrates the distinct temperature dependence of interception between linear and hyperbranched chains. Ultra-low molecular weight components were included as internal standards for concentration correction. Specifically, for linear chains at two characteristic temperatures (*T* = 35 °C and 40 °C), the critical interception size shifted drastically from *R*_h,c_ ≈ 35 nm to 55 nm. In contrast, for hyperbranched systems, we systematically expanded the measurements across a broader temperature window (30, 35, 40, and 45 °C) spanning the *T*_θ_. As explicitly shown in Fig. 7c, while a minor conformational compaction at the sub-*θ* temperature (30 °C) leads to a slightly higher permeation, the SEC elution profiles from 35 °C to 45 °C overlap almost perfectly. Within this wide good-solvent regime, the corresponding critical interception sizes remained nearly constant at ~20–23 nm, demonstrating a strong topological suppression of thermal swelling.

It is important to emphasize that lowering the temperature introduces two competing effects: reduced conformational dimensions, which favor diffusion, and enhanced intrachain excluded volume, which promotes molecular interception. In branched systems, experimental results clearly indicate that the latter dominates. Control experiments consistently demonstrate that branching topology is the key factor governing the interplay between conformational size and excluded volume. These findings imply that for weakly branched or linear polymers, interception can be effectively tuned by temperature via conformational modulation. As shown in Fig. 7d, a narrow temperature window can shift linear chains from nearly complete interception to nearly complete translocation. This phenomenon provides a robust basis for developing novel separation and characterization strategies—for instance, enabling temperature-regulated transport and interception of components with similar sizes but different chemical compositions or branching architectures by exploiting differences in their conformational temperature dependence.

The pronounced temperature dependence of linear chains—contrasted with the near-invariance of hyperbranched chains—strongly suggests that interception in the weak-compression limit is governed not merely by chain dimensions, but by topology-modulated conformational fluctuations and excluded volume interactions. To directly probe these molecular-scale features, we turn to all-atom molecular dynamics (MD) simulations.

### Conformational distribution and fluctuation by computational simulations

**Fig. 8:**
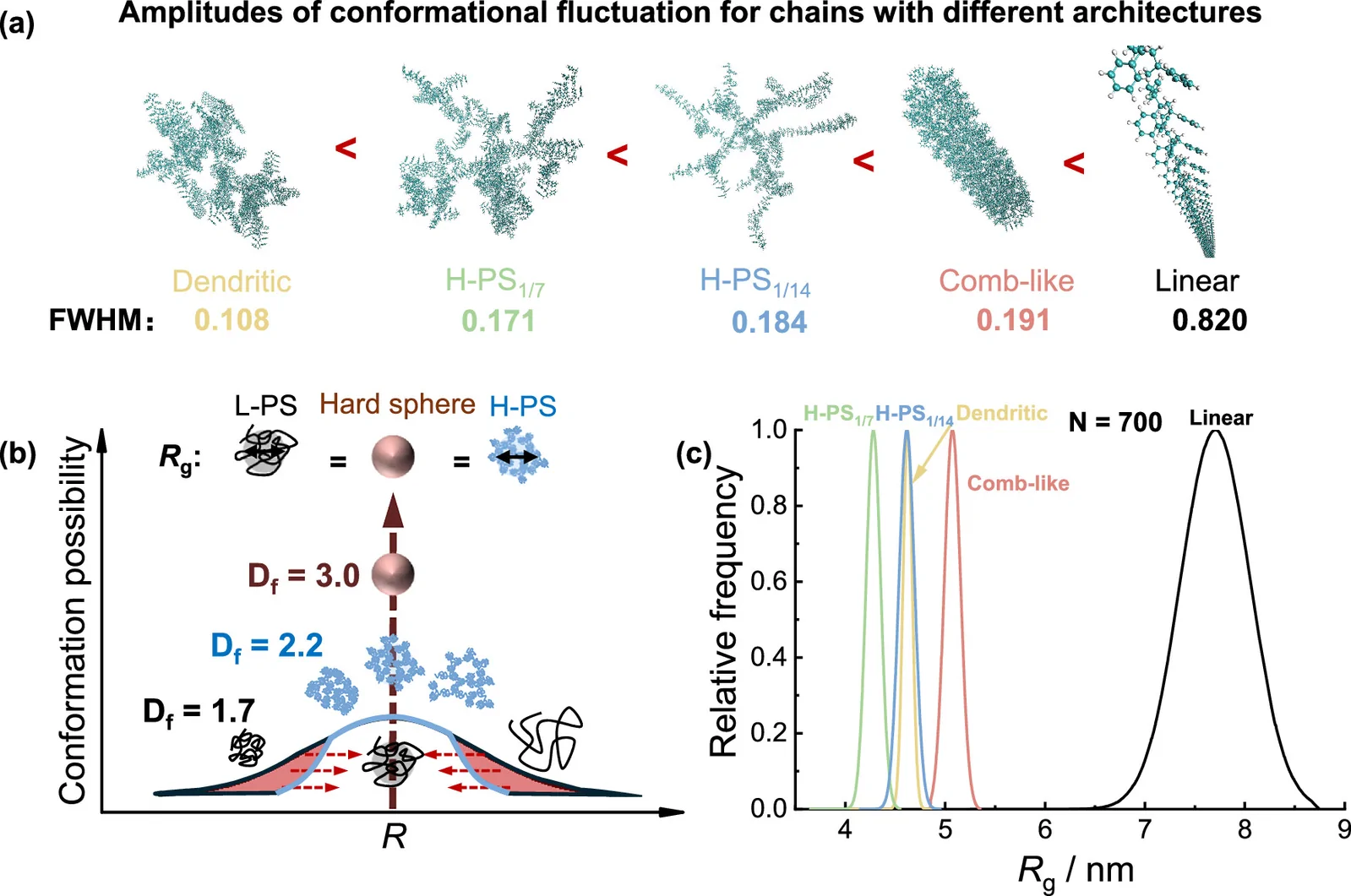
Topology-dependent conformational distributions and fluctuation amplitudes. **a** Representative late-stage conformations and ranking of the amplitudes of radius-of-gyration (*R*_g_) fluctuations for linear, comb-like, hyperbranched, and dendritic chains in all-atom molecular dynamics (MD) simulations. Fluctuation amplitudes are quantified using the full width at half maximum (FWHM, in nm) of the *R*_g_ distributions. H-PS_1/7_ and H-PS_1/14_ denote hyperbranched polystyrene systems containing ~7 and 14 monomers per branch, respectively. **b** Schematic comparison of linear polystyrene (L-PS), hyperbranched polystyrene (H-PS), and an ideal hard sphere at the same *R*_g_. The broad black and narrower blue curves represent the accessible conformational distributions of L-PS and H-PS, respectively. The salmon-shaded regions indicate conformations accessible to L-PS but suppressed by branching in H-PS. Red horizontal arrows denote the range of conformational fluctuations, whereas the upward brown arrow indicates increasing fractal dimension (*D*_f_) and compactness from L-PS (*D*_f_ = 1.7), through H-PS (*D*_f_ = 2.2), to the hard-sphere limit (*D*_f_ = 3.0). **c** Relative-frequency distributions of *R*_g_ for linear (black), comb-like (red), H-PS_1/14_ (blue), H-PS_1/7_ (green), and dendritic (yellow) chains obtained from all-atom MD simulations. Each simulated polymer contains 700 monomer units.

The experimentally observed scaling suggests that branching not only reduces chain dimensions but also alters the free-energy landscape for pore entry. To elucidate the molecular origin of topology-dependent conformational properties, we performed all-atom MD simulations on polymer systems with distinct architectures—linear, comb-like, hyperbranched, and dendritic—each comprising 700 monomer units. Figure 8a schematically outlines these molecular architectures, setting the structural foundation for comparative analysis. Given the immense topological complexity of these large macromolecules, achieving absolute global equilibration is fundamentally challenging. To rigorously overcome local conformational minima, the systems were initially subjected to a 100 ns cyclic annealing protocol (Supplementary Fig. 15). Subsequent extended constant-temperature simulations (up to 120 ns) confirmed that the key structural observable, *R*_g_, had reached a stable late-stage plateau without persistent monotonic drift, thereby ensuring the statistical robustness of our comparative topological analysis. Although hyperbranched polymers are conceptually positioned to exhibit conformational fluctuations intermediate between flexible linear chains and rigid, non-fluctuating hard spheres, the precise quantitative role of branching topology in modulating these dynamics remained unresolved.

Accordingly, the simulations targeted four interrelated aspects inaccessible to direct experiment: (1) equilibrium size distributions and shape anisotropy across topologies; (2) time-dependent dimensional fluctuations and their correlation with branch length; (3) spatial monomer density profiles and their influence on effective excluded volume; and (4) the interplay among chain configuration, branch density, and rigidity under confinement-mimicking conditions. Hyperbranched variants were explicitly designed with two branch densities, ~7 and 14 monomers per branch, in order to isolate architectural effects.

To elucidate the molecular origins of topology-dependent interception, we examined the simulated radius-of-gyration (*R*_g_) distributions (Fig. 8c). The full width at half maximum (FWHM, in nm) of *R*_g_ serves as a precise quantitative metric for conformational fluctuations. As shown in Fig. 8a, increasing branching density progressively suppresses conformational freedom, with the FWHM narrowing significantly from highly flexible linear chains (0.820) to comb-like (0.191), hyperbranched (0.171-0.184), and dendritic architectures (0.108).

The schematic in Fig. 8b places this dynamic trend in a structural context by comparing L-PS, H-PS, and an ideal hard sphere at the same *R*_g_. Despite their comparable overall dimensions, the broad L-PS distribution indicates a larger ensemble of accessible coil conformations. By contrast, the narrower H-PS distribution indicates restricted conformational excursions, with the shaded difference representing conformational space suppressed by branching. This restriction is accompanied by an increase in fractal dimension (*D*_f_), from 1.7 for swollen linear coils to 2.2 for hyperbranched polymers. The latter approaches the non-fluctuating hard-sphere limit of *D*_f_ = 3.0. Thus, branching couples reduced fluctuation amplitude with increased spatial compactness, positioning hyperbranched polymers between flexible linear coils and rigid particles.

Collectively, these findings confirm that branching topology imposes a strict conformational entropy bottleneck. To semi-quantitatively bridge these simulated static dimensions (*R*_g_) with the experimentally determined hydrodynamic radius (*R*_h_), we note that hyperbranching induces a highly compact architecture with a significantly reduced shape factor (*R*_g_/*R*_h_ ≈ 1.08). This tighter coupling of spatial mass distribution and hydrodynamic size, synergizing with the drastically narrowed FWHM, directly raises the free-energy barrier for pore entry, inherently shifting the critical interception (*R*_h,c_) to a smaller relative size.

### Theoretical analysis based on Flory's argument and blob picture

Based on the experimentally observed power-law relationship between the critical interception size and branching density (2*R*_h,c_/*D* ~ *ρ*^−1/2^), we further employed a combination of Flory mean-field theory and the blob model to theoretically analyze the potential dependence of the diffusion free energy barrier on molecular topology. According to the mean-field theory for a single polymer chain^41,42^, the free energy of a polymer chain with arbitrary architecture can be expressed as:

1 $$\frac{\Delta F}{{k}_{B}T}\approx \frac{{R}^{2}}{{{R}_{0}}^{2}}+\frac{{b}^{3}}{V}{{N}_{{{{\rm{total}}}}}}^{2}$$

where the first term on the right-hand side represents the contribution of conformational entropy, and the second term represents the contribution of monomer-monomer interactions. *R* and *R*_0_ denote the real and ideal chain dimensions, respectively; *V* ~ *R*^3^ is the occupied volume of the chain; *b* is the monomer size, and *N* is the degree of polymerization. Minimizing Eq. (1) with respect to *R* yields the chain size in a good solvent for arbitrary topology:

2 $$R\sim {({{R}_{0}}^{2}{{N}_{{{{\rm{total}}}}}}^{2}{b}^{3})}^{\frac{1}{5}}$$

On the other hand, the ideal dimension of linear and hyperbranched chains can be expressed as:

3 $${R}_{0}\sim {{N}_{{{{\rm{total}}}}}}^{\frac{1}{4}}{{N}_{{{{\rm{sub}}}}}}^{\frac{1}{4}}b$$

where *N*_total_ and *N*_sub_ are the degrees of polymerization for the whole chain and the branch chain, respectively, and the branching density *ρ* = 1/*N*_sub_. For linear chains (*N*_total_ = *N*_sub_), *α* = 1/2, *β* = 0; for hyperbranched chains, *α* = 1/4, *β* = 1/4. Substituting Eq. (3) into Eq. (2) gives the chain size in a good solvent:

4a $$R\sim {{N}_{{{{\rm{total}}}}}}^{\alpha }{{N}_{{{{\rm{sub}}}}}}^{\beta }b$$

Specifically, for linear and hyperbranched chains, *R* is expressed as:

$R\sim {{N}_{{{{\rm{total}}}}}}^{\frac{3}{5}}b$ Linear chain 4b $R\sim {{N}_{{\rm{total}}}}^{\frac{1}{2}}{{N}_{{\rm{sub}}}}^{\frac{1}{10}}b$ Hyperbranched chain 4c.

Similarly, when a chain is confined within a cylindrical pore of diameter *D*, its confined length (*L*) can be obtained by minimizing a modified free energy expression:

5 $$\frac{\Delta F}{{k}_{B}T}=\frac{{L}^{2}}{{{R}_{0}}^{2}}+\frac{{b}^{3}}{L{D}^{2}}{{N}_{{{{\rm{total}}}}}}^{2}$$

6 $$L\sim {\left(\frac{{{R}_{0}}^{2}{b}^{3}{{N}_{{{{\rm{total}}}}}}^{2}}{{D}^{2}}\right)}^{\frac{1}{3}}$$

Combining Eqs. (4) and (6) gives the normalized confined length *L* and critical confined volume (*V*):

7 $$\frac{L}{D}\sim {\left(\frac{R}{D}\right)}^{\frac{5}{3}}$$

8 $$V=L{D}^{2}\sim {D}^{3}{\left(\frac{R}{D}\right)}^{\frac{5}{3}}$$

This indicates that the confinement scale primarily depends on the initial chain size and the pore diameter. Although Flory’s mean field theory can accurately provide the dimensions of polymer chains before and after being restricted, the estimation of free energy deviates significantly from the true value^41,42^. To analyze the activation energy, we use the blob model, treating the confined chain as a string of blobs of size *ξ*. The size of each blob satisfies:

9 $$\xi \sim {{N}_{\xi }}^{\alpha {\prime} }{{N}_{{\rm{sub}}}}^{\beta {\prime} }b$$

where *N*_*ξ*_ is the number of monomers per blob. According to the segment density conservation:

10 $$\frac{{N}_{{{{\rm{total}}}}}}{V}=\frac{{N}_{\xi }}{{\xi }^{3}}$$

Combining Eqs. (4), (9), and (10) yields:

11 $$\xi \sim {\left(\frac{{N}_{{{{\rm{total}}}}}{{N}_{{{{\rm{sub}}}}}}^{\frac{\beta {\prime} }{\alpha {\prime} }}}{{R}^{\frac{5}{3}}{D}^{\frac{4}{3}}}\right)^{\frac{\alpha {\prime} }{1-3\alpha {\prime} }}}\sim {\left[{R}^{(\frac{\alpha {\prime} }{\alpha }-\frac{5\alpha {\prime} }{3})}{N}_{{\rm{sub}}}^{(\beta {\prime} -\frac{\alpha {\prime} }{\alpha }\beta )}\right]}^{\frac{1}{1-3\alpha {\prime} }}\,{D}^{-\frac{4\alpha {\prime} }{3(1-3\alpha {\prime} )}}$$

For linear chains, assuming the chain structure and conformation within a blob remain consistent with the unperturbed state (where *N*_total_ = *N*_sub_, *α*' = *α* = 3/5, and *β*' = *β* = 0), the blob size scales as *ξ* ~ *D*. In contrast, for hyperbranched chains under the same assumption (*α*' = *α* = 1/2, *β*' = *β* = 1/10), the blob size scales as *ξ* ~ (*R*/*D*)^−1/3^*D*. This highlights a key difference: the blob size in hyperbranched chains depends strongly on both the original chain size and pore diameter, unlike in linear chains.

Under the condition of a weak compression limit, a central question is whether the energy barrier for diffusion through the pore depends solely on the initial chain size or also on the branching density. We thus analyze the normalized energy barrier Δ*E*/(*k*_B_*T*), which is proportional to the total number of blobs in the nanoconfined space.

12 $$\frac{\Delta E}{{k}_{B}T}\sim \frac{{N}_{{{{\rm{total}}}}}}{{N}_{\xi }}=\frac{L{D}^{2}}{{\xi }^{3}}$$

Using Eqs. (7) and (11), and noting that *N*_sub_ ~ *ρ*^−1^, we derive:

13 $$\frac{\Delta E}{{k}_{B}T}\sim \frac{1}{{\xi }^{3}}\sim {{N}_{{{{\rm{sub}}}}}}^{\frac{3}{3\alpha ^{\prime} -1}(\frac{\alpha \beta ^{\prime} -\alpha {\prime} \beta }{\alpha })}$$

In Eq. (13), the sign of the exponent (*αβ*' − *α*'*β*) is critical. For hyperbranched chains in the free state, *α* = 1/2 and *β* = 1/10. Under weak confinement at the interception boundary, chain segment density more or less increases, leading to *α*' <*α* and *β*' <*β*, with *α*' + *β*' <3/5. For example, in the strong confinement limit (hard-sphere regime), *α*' = 1/3 and *β*' = 0 result in (*αβ*' − *α*'*β*) = −1/30. This negative exponent confirms that Δ*E* slightly decreases with *N*_sub_. Considering that *ρ* = 1/*N*_sub_, Δ*E* actually slightly increases with the branching density *ρ*. Simple numerical analysis across plausible values (e.g., *α*‘ ranging from 1/3 to 1/2, *β*‘ from 0 to 1/10) consistently yields (*αβ*' − *α'β*) <0, supporting the generality of this trend.

This theoretical framework qualitatively supports the experimental observation: higher branching density introduces a slightly higher free energy barrier for chain diffusion through nanochannels. Thus, the *ρ*^−1/2^ scaling in interception size arises not only from compact chain architectures but also from topology-dependent energy barriers, providing a mechanistic foundation for the observed phenomena. It is worth noting that while the qualitative blob analysis conceptually supports the scaling of the free-energy barrier, a rigorous derivation of the exact −1/2 exponent from first principles remains challenging. Classical theories rely on average conformations and largely overlook the width of the conformational distribution. As our simulations revealed, the drastically narrowed FWHM of *R*_g_ critically determines the interception. Therefore, the observed scaling is currently regarded as a robust empirical observation, highlighting a genuine theoretical gap regarding how topological constraints and many-body interactions restrict conformational fluctuations.

## Discussion

This study systematically investigated the nanoscale transport behavior of polymers with varying topological architectures through an integrated approach combining precisely synthesized H-PS samples, well-defined AAO nanopores, all-atom MD simulations, and theoretical scaling analysis. By decoupling the effects of branching density, pore geometry, and flow conditions, we established a comprehensive structure-transport relationship under the weak compression limits. Key findings reveal that: (1) The critical interception size (*R*_h,c_) exhibits a distinct power-law dependence on branching density (*ρ*), roughly described by the scaling of *R*_h,c_ ~ *ρ*^−1/2^, which is significantly smaller than that of linear chains across pore diameters ranging from 28 to 96 nm. (2) While temperature variation noticeably alters the dimensions of linear chains and their interception behavior, *R*_h__,c_ remains nearly constant for hyperbranched architectures, underscoring that topology—rather than solvent-mediated excluded volume effects—dominates the interception process in the weak-flow regime. (3) Combined theoretical and simulation analyses demonstrate that the free energy barrier for pore translocation is strongly topology-dependent, with hyperbranched chains facing a higher entropic barrier due to restricted conformational fluctuations.

The underlying physical origin of these observations can be mainly attributed to two interrelated mechanisms: the topology-dependent free energy barrier and the conformational distribution breadth. Hyperbranched chains, with their compact and rigid architectures, undergo limited conformational fluctuations, leading to a narrowed distribution curve and a higher energy barrier for pore entry. In contrast, linear chains possess broad conformational distributions, enabling a finite probability of transiently contracting to a smaller size and thereby broadening the effective interception size. These two mechanisms are intrinsically linked—the confined fluctuations of hyperbranched chains directly raise the initial free energy barrier for translocation.

This work not only resolves long-standing questions regarding the role of branching topology in nanopore transport but also establishes a unified physical framework for understanding polymer translocation across biological and synthetic systems. The insight that molecular architecture can be leveraged to tune transport selectivity offers promising avenues for the design of advanced separation membranes, nanofiltration systems, and biomimetic channels. Future studies extending these principles to dynamic conditions, polydisperse systems, and heterogeneous pore networks could further unlock the potential of topology-engineered polymers in nanoscale fluidic applications.

## Methods

### Materials

Styrene was passed through basic alumina, distilled under reduced pressure, and stored at −18 °C. Azobisisobutyronitrile was recrystallized from methanol. Copper(I) bromide was washed successively with glacial acetic acid and ethanol and dried under vacuum. Dimethylformamide was dried over anhydrous magnesium sulfate and distilled under reduced pressure. Toluene, triethylamine, dichloromethane, anisole, and tetrahydrofuran were distilled over calcium hydride. All other reagents were used as received. Suppliers, purities, and complete handling procedures are provided in Supplementary Methods.

### Characterization

SEC was performed using a BWAIC P98II HPLC pump, Shodex KF-806, KF-804, and KF-802.5 columns, and a Shodex RI-201H refractive index detector. Tetrahydrofuran was used as the mobile phase at 1.0 mL min^−1^. The system was calibrated with narrow-dispersity polystyrene standards of 2,050, 9,460, 28,500, 68,000, 156,000, 696,000, and 3,500,000 g mol^−1^. Proton nuclear magnetic resonance (^1^H NMR) spectra were recorded at room temperature on a Bruker Avance III Ascend 400 MHz spectrometer using CDCl_3_ containing tetramethylsilane. Fourier-transform infrared spectra were acquired on a Bruker VECTOR-22 spectrometer using KBr pellets, 64 scans, and a resolution of 4 cm^−1^.

### Synthesis of the AB₂ initiator and polymer samples

The AB_2_ initiator, propargyl 2,2-bis[(2-bromo-2-methylpropanoyloxy)methyl]propionate, was prepared through a four-step route adapted from a reported procedure^43^. 2,2-Bis(hydroxymethyl)propionic acid (50.0 g) was protected with 2,2-dimethoxypropane and p-toluenesulfonic acid in acetone for 4 h at room temperature. The protected intermediate was coupled with propargyl alcohol using dicyclohexylcarbodiimide and 4-(dimethylamino)pyridine in dichloromethane overnight. Acidic deprotection was conducted using 1 M HCl in tetrahydrofuran for 4 h. The resulting diol was reacted with 2-bromoisobutyryl bromide and triethylamine in dichloromethane at 0 °C, followed by overnight reaction at room temperature. The product was purified by silica-gel chromatography and confirmed by ^1^H NMR spectroscopy (400 MHz, CDCl_3_ containing 0.03% tetramethylsilane; Supplementary Fig. 3a). Exact reagent quantities, work-up procedures, and intermediate purification steps are provided in Supplementary Methods.

AB_2_ polystyrene macromonomers were prepared by ATRP. A representative reaction contained AB_2_ initiator (0.251 g), Me_6_TREN (0.123 g), Sn(EH) _2_ (0.216 g), styrene (100 g), anisole (110 mL), and CuBr (0.077 g). After three freeze-pump-thaw cycles, polymerization proceeded at 80 °C for 11 h. The product was passed through alumina and precipitated into cold methanol. Bromine termini were converted to azides using NaN_3_ in dimethylformamide for 36 h at room temperature. Hyperbranched polystyrene was then formed by Cu(I)-catalyzed azide–alkyne cycloaddition. A representative reaction used PS_340_ (200 mg), PMDETA (10.4 mg), CuBr (4.79 mg), and dimethylformamide (2 mL) at 40 °C for 24 h. Other macromonomers and hyperbranched samples were prepared analogously, as detailed in Supplementary Methods and Supplementary Table 1.

### Fabrication and characterization of AAO membranes

High-purity aluminum foils were electropolished in 65% HClO_4_/ethanol (1:4, v/v) at 20 V and 10 °C. Ordered AAO membranes were prepared by two-step anodization in 0.3 M oxalic acid at 7 °C and 40 V. The first anodization lasted 18 h for *D*_1_–*D*_3_ and 36 h for *D*_4_-*D*_7_. The oxide layer was removed using 1.8 wt% H_2_CrO_4_ and 6 wt% H_3_PO_4_ at 45 °C for 8 h, followed by a 24 h anodization. Residual aluminum was dissolved in CuCl_2_/HCl solution. Double-through pores were opened in 5 wt% H_3_PO_4_ at 30 °C for 10–120 min, producing membranes with effective pore diameters of 28–96 nm. Pore morphology and size distributions were measured by field-emission scanning electron microscopy (Supplementary Table 3). Detailed solution compositions and etching times are provided in Supplementary Methods.

### Polymer interception under weak-compression conditions

Polymer solutions were prepared at concentrations below 1.00 g L^−1^, corresponding to less than one-tenth of the critical overlap concentration. An aliquot of each stock solution was analyzed by SEC before interception. Each AAO membrane was mounted in a custom stainless-steel holder connected to a pressure sensor and maintained at 25 ± 1 °C. Pure solvent was first pumped through the membrane at 6.0 mL h^−1^. The steady-state transmembrane pressure was monitored for at least 10 min, and the effective pore diameter was calculated using the Poiseuille equation.

The feed was then changed to the polymer stock solution, and the flow rate was reduced to 0.2 mL h^−1^. Under these conditions, the estimated shear energy was ~10^−6^ *k*_B_ *T*, where *k*_B_ is the Boltzmann constant, and *T* is the absolute temperature. The first 250 μL of filtrate was discarded to remove solvent from the dead volume. A subsequent 250 μL permeate fraction was collected and analyzed immediately by SEC. Full assembly, calibration, and sampling procedures are provided in Supplementary Methods.

### Determination of translocation probability and critical interception size

The translocation probability *P*_t_ was calculated from the normalized SEC signals as *P*_t_ = *C*/*C*_0_, where *C* and *C*_0_ are the normalized polymer concentrations in the permeate and stock solutions, respectively. The critical retention point was converted to the critical hydrodynamic interception radius *R*_h,c_ using the SEC calibration. The uncertainty in *P*_t_ was ±3%. The uncertainty in *R*_h,c_ was propagated from the SEC normalization and the AAO pore-size distribution.

### MD simulations

Polystyrene models containing 700 styrene units were generated using Sobtop^44^. Five initial conformations were prepared, and Amber99SB partial charges and force-field parameters were assigned^45^. Each structure was placed in a 30 nm × 30 nm × 30 nm periodic box containing toluene. Simulations were performed using GROMACS 2022.5^46^ with a 2 fs integration time step. Energy minimization was followed by 20 ns of NPT equilibration at 300 K and 1 bar. A 100 ns cyclic-annealing protocol was then conducted in the NVT ensemble by heating from 300 to 378 K and cooling to 300 K. Short-range interactions used a Verlet neighbor list, long-range electrostatics used particle-mesh Ewald summation, and bonds involving hydrogen were constrained. Radius-of-gyration trajectories were calculated using GROMACS analysis tools, and molecular structures were inspected using VMD^47^. Expanded model and simulation information is provided in Supplementary Methods.

## Supplementary information

Supplementary Information

Transparent Peer Review file

## Source data

Source data

## Data Availability

The data that support the plots within this paper and other findings of this study are presented in the main article and the Supplementary Information. All data are available from the corresponding author upon request. Source data are provided with this paper.
