# Supplementary material for: Conformational entropy bottleneck in polymer nanopore interception driven by branching architecture and excluded volume
Source: Nat Commun. 2026 Sep 29;17:11231. doi: 10.1038/s41467-026-78055-1 (PMC13720087; doi:10.1038/s41467-026-78055-1)
Supplement: Supplementary file 1 [file 41467_2026_78055_MOESM1_ESM.pdf]

# Supplementary Information

# For

# Conformational Entropy Bottleneck in Polymer Nanopore Interception Driven by Branching Architecture and Excluded Volume

Yiren Wang[1], Haorong Huang[1], Jiawen Yang[2], Xin Guan*[,1], Mo Zhu[1], Mingming Ding[2] & Lianwei Li*[,1]

[1] College of Chemistry and Environmental Engineering, Shenzhen University, Shenzhen, China 518060

[2] School of Chemical Engineering and Light Industry, Guangdong University of Technology, Guangzhou, China 510006

# Content

**1. Supplementary Methods............................................................3**

**2. Summarized molecular parameters of H-PS samples..............................9**

**3. Summarized molecular parameters of L-PS samples..............................10**

**4. Summarized information of nanochannels..........................................10**

**5. SEC curve of $AB_2$-type PS macromonomers.......................................11**

**6. FTIR spectra of bromine-terminated PS macromonomers, azide-terminated PS macromonomers and hyperbranched PS ...............................................11**

**7. $^1$H NMR spectra of $AB_2$-type initiator, bromine-terminated PS macromonomer and azide-terminated PS macromonomer...................................................12**

**8. SEC curves of standard mixtures of linear PS standard samples...................13**

**9. Scaling law between the size and molecular weight of PS in toluene.............13**

**10. Apparent radius of gyration ($R_{g,app}$) profiles.......................................14**

**11. Schematic representations of model polymers and semilog plots of $M[\eta]$ versus elution volume.......................................................................15**

**12. Preparative SEC fractionation of linear and hyperbranched polystyrene samples...........15**

**13. Dynamic light scattering (DLS) measurements of the co-eluting fractions.........................16**

**14. Transmembrane pressure and Poiseuille pore diameter for AAO-$D_n$................................17**

**15. Stability test of the membrane before and after the interception experiment....................17**

**16. Dependence of transmembrane pressure on solvent viscosity.............................................18**

**17. Dependence of translocation probability on retention volume...............................................18**

**18. Determination of the critical interception size.......................................................................19**

**19. Time evolution of the radius of gyration ($R_g$) over 120 ns all-atom molecular dynamics simulations...........................................................................................................................19**

**20. Reference...........................................................................................................................20**

## Supplementary Methods

These Supplementary Methods provide expanded reagent handling, synthetic work-up, instrumental settings and procedural details supporting the Methods section of the main manuscript.

### Reagents and analytical methods

**Materials.** All chemicals, unless otherwise specified, were purchased from commercial suppliers and used without further purification. Styrene (Alfa Aesar, AR) was purified by passage through a basic alumina column to remove inhibitors and subsequently distilled under reduced pressure before storage at -18 °C. Toluene (Sigma-Aldrich, 99%), triethylamine (TEA, Sinopharm, 99%), dichloromethane (DCM, General-Reagent, AR), anisole (Sigma-Aldrich, 99%) and tetrahydrofuran (THF, Aladdin, AR) were distilled over calcium hydride prior to use. Cyclopentane (Sinopharm, 99.5%) was used as received. Azobisisobutyronitrile (AIBN, TCI, 98%) was recrystallized from methanol. Copper(I) bromide (CuBr, Sigma-Aldrich, 98%) was washed with glacial acetic acid to remove soluble oxidized species, followed by ethanol, and dried under vacuum. Dimethylformamide (DMF, General-Reagent, AR) was dried over anhydrous magnesium sulfate and then distilled under reduced pressure. Aluminum foils (Aladdin, 99.999%), methanol (Aladdin, AR), tris(2-(dimethylamino)ethyl)amine ($Me_6$TREN, TCI, 98%), pentamethyldiethylenetriamine (PMDETA, Alfa Aesar, 99%), tin(II)-2-ethylhexanoate ($Sn(EH)_2$, Sigma-Aldrich, 95%), 2,2-bis(hydroxymethyl) propionic acid (DMPA, Aladdin, 97%), 4-(dimethylamino)pyridine (DMAP, Aladdin, 99%), dicyclohexyl carbodiimide (DCC, Aladdin, 99%), p-toluenesulfonic acid monohydrate (PTSA, Aladdin, 99%), 2,2-dimethoxypropane (DMP, Aladdin, 99%), propargyl alcohol (Sigma-Aldrich, 99%), perchloric acid ($HClO_4$, Aladdin, 70%), sodium azide ($NaN_3$, Aladdin, 99%), ethanol (Sinopharm, AR), oxalic acid (Aladdin, 99%), chromic acid ($H_2CrO_4$, Dayang Chem, 98%), phosphoric acid ($H_3PO_4$, Alfa Aesar, 85%), copper(II) chloride dihydrate ($CuCl_2 \cdot 2H_2O$, Alfa Aesar, 99%), and hydrochloric acid (HCl, Sinopharm, AR) were used as received without additional treatment.

**Size Exclusion Chromatography (SEC).** The molecular weights (number-average $M_n$, weight-average $M_w$, and peak $M_p$) along with the polydispersity index ($M_w/M_n$) of polystyrene and related intermediate samples were determined by single-detector size exclusion chromatography (SEC). The system consisted of a BWAIC P98II HPLC pump and a series of Shodex KF-800 columns (KF-806, KF-804, and KF-802.5; each 8 × 300 mm), providing effective separation across molecular weight ranges of approximately $1.5 \times 10^5$ - $2 \times 10^7$ g/mol, $7 \times 10^3$ - $3 \times 10^5$ g/mol, and $3 \times 10^2$ - $8 \times 10^3$ g/mol, respectively. Detection was carried out using a Shodex RI-201H refractive index detector placed downstream of the column set. HPLC-grade tetrahydrofuran (THF) was employed as the mobile phase at a flow rate of 1.0 mL/min. The system was calibrated with a series of narrow-dispersity polystyrene standards with molecular weights of 2050, 9460, 28500, 68000, 156000, 696000, and 3500000 g/mol.

**Proton Nuclear Magnetic Resonance Spectroscopy ($^1$H-NMR).** All $^1$H NMR measurements were performed at room temperature using a Bruker Avance III Ascend 400 MHz spectrometer. Samples were dissolved in deuterated chloroform ($CDCl_3$) containing tetramethylsilane (TMS) as an internal standard for chemical shift calibration.

**Fourier Transform Infrared Spectroscopy (FTIR).** Measurements were conducted using a Bruker VECTOR-22 spectrometer. The sample was mixed with potassium bromide at a mass ratio of 1:100, and then pressed into small pellet for measurement. Spectral data were acquired with 64 cumulative scans at a resolution of 4 $cm^{-1}$.

**Synthesis of the $AB_2$ initiator and polymer samples**

**Synthesis of propargyl 2,2-bis[(2-bromo-2-methylpropanoyloxy)methyl] propionate**

The $AB_2$ initiator was prepared through a four-step procedure adapted from the reported synthetic route[1].

For the protection step, 2,2-bis(hydroxymethyl)propionic acid (50.0 g, 372.8 mmol), 2,2-dimethoxypropane (69.0 mL, 559.0 mmol), and *p*-toluenesulfonic acid monohydrate (3.50 g, 18.5 mmol) were dissolved in acetone (200 mL). The solution was maintained at room temperature for 4 h. The acid catalyst was then quenched with 5.0 mL of a 1:1 (v/v) mixture of 25% aqueous ammonia and ethanol. After removal of acetone under reduced pressure, the residue was taken up in DCM (500 mL). The organic phase was washed with water (2 × 150 mL), dried over anhydrous $Na_2SO_4$, and concentrated to obtain protected intermediate I.

Intermediate I (49.7 g, 286.7 mmol), propargyl alcohol (24.5 mL, 426.7 mmol), and 4-(dimethylamino)pyridine (17.0 g, 140.0 mmol) were dissolved in DCM (500 mL). After 10 min, dicyclohexylcarbodiimide (70.0 g, 340.0 mmol) in DCM (200 mL) was introduced dropwise over 30 min under nitrogen. The reaction was allowed to proceed overnight at room temperature. The resulting dicyclohexylurea was removed by filtration, and the filtrate was diluted with DCM (2 × 500 mL). The solution was washed with water (2 × 250 mL), dried over anhydrous $Na_2SO_4$, and evaporated. Silica-gel chromatography with hexane/ethyl acetate (25:1, v/v) afforded propargyl 2,2,5-trimethyl-1,3-dioxane-5-carboxylate (II).

Compound II (16.7 g, 79.2 mmol) was combined with 1 M HCl (41.7 mL) and THF (41.7 mL). After stirring at room temperature for 4 h, the precipitated material was removed by filtration. The filtrate was diluted with DCM (500 mL) and washed with water (2 × 100 mL). The organic phase was dried over anhydrous $Na_2SO_4$ and concentrated. Addition of hexane followed by storage in a deep freezer for 48 h yielded propargyl 3-hydroxy-2-(hydroxymethyl)-2-methylpropanoate (III).

Compound III (7.50 g, 43.3 mmol) and triethylamine (13.3 mL, 95.8 mmol) were dissolved in DCM (150 mL) and cooled to 0 °C. A solution of 2-bromoisobutyryl bromide (10.1 g, 43.3 mmol) in DCM (100 mL) was added over 30 min. The mixture was subsequently stirred overnight. After filtration,

the mixture was partitioned between DCM (300 mL) and saturated aqueous $NaHCO_3$ (80 mL). The aqueous phase was further extracted with DCM (200 mL). The combined organic layers were dried over anhydrous $Na_2SO_4$ and concentrated. The residue was purified by silica-gel chromatography using hexane/ethyl acetate (4:1, v/v), affording propargyl 2,2-bis[(2-bromo-2-methylpropanoyloxy)methyl]propionate. Product identity was confirmed by $^{1}$H NMR spectroscopy (400 MHz, $CDCl_3$ containing 0.03% TMS; Supplementary Fig. 3a).

**Preparation of $AB_2$ Polystyrene Macromonomers.** In a typical synthesis, propargyl 2,2-bis[(2-bromo-2-methylpropanoyloxy)methyl]propionate (0.251 g, 0.534 mmol), $Me_6$TREN (0.123 g, 0.534 mmol), $Sn(EH)_2$ (0.216 g, 0.534 mmol), styrene (100 g, 0.962 mol), and anisole (110 mL) were successively added to a 250 mL glass tube equipped with a magnetic stir bar. The mixture was stirred until homogeneous, degassed via three freeze-pump-thaw cycles, and charged with CuBr (0.077 g, 0.053 mmol) while frozen. The sealed tube was immersed in an oil bath at 80 °C for 11.0 h, then quenched in liquid nitrogen. The crude product was diluted with THF, passed through a short alumina column to remove metal residues, and concentrated by rotary evaporation. The polymer was precipitated into cold methanol, collected, and dried under vacuum at 40 °C to afford the target macromonomer (yield 14.26 g, 78.5%; $M_w = 3.41 \times 10^4$ g/mol). Other PS macromonomers were prepared analogously (Supplementary Fig. 1).

**Azidation Substitution Reaction.** 4.0 g (0.12 mmol) of alkynyl-(polystyrene-bromine)$_2$, 50 mL of N,N-dimethylformamide (DMF), and 0.300 g (4.70 mmol) of sodium azide were added to a 100 mL round-bottom flask. The mixture was stirred at room temperature for 36 hours. After removing most of the solvent under reduced pressure, the remaining portion was diluted with tetrahydrofuran, and the supernatant was collected after high-speed centrifugation and poured into an excess of cold methanol for precipitation. The precipitate was dried overnight in a vacuum oven at room temperature to obtain an $AB_2$ polystyrene macromonomer (yield 3.40 g, 85%) containing one alkynyl functional group and two azide functional groups. Following the same experimental procedure, the bromine-terminated PS monomers of other molecular weights were replaced with azide-terminated $AB_2$ macromonomers.

**Preparation of Hyperbranched Samples from $AB_2$ Macromonomers via Azide−Alkyne Cycloaddition Reaction.** A 5 mL dried glass tube equipped with a magnetic stir bar was charged successively with $PS_{340}$ (200 mg, 0.006 mmol), PMDETA (10.4 mg, 0.06 mmol), and DMF (2 mL). After thorough mixing, the solution was degassed via two freeze–vacuum–thaw cycles. CuBr (4.79 mg, 0.03 mmol) was then added to the frozen mixture, and the tube was subjected to an additional freeze–vacuum–thaw cycle before being sealed under vacuum. The sealed tube was immersed in an oil bath at 40 °C for 24 h. After reaction, the mixture was diluted with THF and quickly passed through a neutral alumina column. Residual copper salts and ligand were removed by repeated precipitation from THF/methanol. The final product, hyperbranched polystyrene H-$PS_{1/170}$ (entry 8,

Supplementary Table 1), was collected by filtration and dried under vacuum at 45 °C overnight (yield: ~180 mg). Other H-PS samples with varying branching densities and molecular weights (Supplementary Table 1) were prepared following analogous procedures.

### Fabrication of double-through AAO nanochannel membranes

High-purity aluminum foils were electropolished in a 1:4 (v/v) mixture of 65% $HClO_4$ and ethanol at 20 V and 10 °C. Ordered porous templates were fabricated via a two-step anodization process in 0.3 M oxalic acid at 7 °C under 40 V. For membranes $D_1$-$D_3$ (target pore diameters: 28-41 nm; interpore distance: 66 ± 5nm), the first anodization was performed for 18 h, followed by oxide removal in a mixture of 1.8 wt% $H_2CrO_4$ and 6 wt% $H_3PO_4$ at 45 °C for 8 h to generate hexagonal concaves. For membranes $D_4$-$D_7$ (55-96 nm diameter; interpore distance: 99 ± 8 nm), the first anodization duration was extended to 36 h, with identical etching conditions. The second anodization (24 h) yielded highly ordered nanopores. The aluminum substrate was selectively dissolved using a $CuCl_2$/HCl solution (7.0 g $CuCl_2 \cdot 2H_2O$, 100 mL 40 wt% HCl, 100 mL DI water), exposing barrier layers at both ends. Double-through channels were formed by immersing membranes in 5 wt% $H_3PO_4$ at 30 °C with precisely controlled etching durations: $D_1$ (10 min), $D_2$ (15 min), $D_3$ (25 min), $D_4$ (40 min), $D_5$ (60 min), $D_6$ (90 min), and $D_7$ (120 min). This protocol yielded seven distinct double-through AAO membranes with cylindrical pores spanning 28-96 nm in diameter. Morphological fidelity and dimensional uniformity were confirmed by field-emission scanning electron microscopy (FE-SEM), with representative pore structures and size distributions detailed in Supplementary Table 3.

### Polymer interception and data analysis

#### Standardized Protocol for Polymer Interception under the Weak-Compression Limit

Preparation of Ultra-Dilute Polymer Solutions: To isolate the intrinsic interception behavior and fundamentally eliminate artifacts caused by multi-chain synergistic translocation, the polymer stock solutions were prepared at extremely dilute concentrations. The concentration was strictly capped below 1.00 g/L, which is less than 1/10 of the critical overlap concentration ($C$*). This ensures that the polymer chains remain physically isolated without inter-chain entanglement. Following complete dissolution, an initial aliquot of the stock solution was analyzed via Size Exclusion Chromatography (SEC) to establish the baseline molecular weight distribution for subsequent comparison.

Membrane Assembly and In-situ Pore Size Calibration: The fragile AAO membrane was carefully inserted into a custom-made stainless steel bracket with threaded connections. During the sealing process, pure solvent was injected to ensure complete exhaust, and a moderate torque (~30°) was manually applied to the threaded connection area. This procedure guarantees absolute airtightness while preventing excessive mechanical stress that could fracture the membrane. The assembled

module was integrated with a high-sensitivity pressure sensor and placed in a thermostat locked precisely at 25 ± 1 °C. Pure solvent was then pumped through the system at 6 mL/h. By monitoring the steady-state transmembrane pressure drop for over 10 minutes, we verified the patency of the pores and calculated the exact effective hydrodynamic pore size in-situ using the Poiseuille equation.

Weak-Compression Interception and Immediate SEC Analysis: The system was subsequently switched to the polymer stock solution, and the macroscopic flow rate was drastically reduced to an ultra-low level of 0.2 mL/h using a high-precision syringe pump. At this flow rate, the localized shear energy is negligible (~$10^{-6}$ $k_BT$), ensuring the translocation is entirely dominated by Brownian diffusion and conformational entropy rather than flow-induced stretching. To thoroughly flush any residual solvent from the system's dead volume, the initial 250 μL of the filtrate was discarded. Once the system reached a fully stable operational state, exactly 250 μL of the effective permeate was collected. To maximize data fidelity and prevent systemic errors originating from time-dependent column drift, we strictly adhered to an "immediate sampling and testing" protocol, instantly subjecting the collected permeate to SEC analysis.

**Calculation of Translocation Probability ($P_t$) and critical hydrodynamic radius ($R_{h,c}$).** In this work, the translocation probability ($P_t$) was determined by comparing the size-exclusion chromatography (SEC) traces of the hyperbranched polymer stock solution and the permeate collected after nanochannel interception. The SEC curve of the stock solution ($C_0$) served as the concentration reference. The corresponding SEC signal of the permeate ($C$) was normalized to $C_0$ on the same retention volume scale, yielding $P_t$ directly from the ratio of the two chromatograms:

$$P = \frac{C}{C_0}$$

where $C$ and $C_0$ represent the normalized polymer concentrations in the permeate and stock solutions, respectively. This method effectively eliminates signal fluctuations from instrumental and operational variations, allowing a precise and reproducible assessment of the interception behavior. The typical uncertainty in $P_t$ determination was within ± 3%. Error bars for the translocation probability ($P_t$) represent a ± 3% instrument/baseline uncertainty derived from repeated SEC normalization. Horizontal error bars for the critical hydrodynamic radius ($R_{h,c}$) represent the propagated uncertainty, calculated by mapping the $P_t$ error onto the retention volume axis and converting it via the SEC calibration equation. The shaded blue region encompasses the compounded standard deviation considering both the measurement uncertainty of $R_{h,c}$ and the structural size distribution ($\Delta D$) of the AAO nanopores.

## Molecular simulation details

**Model and Simulation Details.** Based on experimental observations and computational efficiency considerations, we constructed a simplified polystyrene (PS) model. Each polymer chain consists of 700 styrene monomer units, and five distinct initial configurations were prepared to investigate

how the starting geometry influences the radius-of-gyration ($R_g$) distribution. The molecular topology was generated using Sobtop[2]. Partial charges and force-field parameters were assigned within the Amber99sb framework[3]. To ensure sufficient relaxation and to avoid trapping in local minima, the initial structures were placed in a 30 nm × 30 nm × 30 nm periodic simulation box. Toluene was used as the solvent to represent the solution environment for conformational and statistical analyses.

Molecular dynamics simulations were performed under periodic boundary conditions using the GROMACS 2022.5 package[4]. The simulation protocol comprised energy minimization (EM), NPT equilibration, and NVT production. EM was first carried out to remove unfavorable contacts. The system was then equilibrated for 20 ns in the NPT ensemble at 300 K and 1 bar with a 2 fs integration time step. Subsequently, a total of 100 ns of cyclic annealing was conducted in the NVT ensemble: the temperature was gradually increased from 300 K to 378 K, held briefly at the elevated temperature to facilitate crossing conformational energy barriers, and then cooled back to 300 K to obtain well-relaxed chain conformations for subsequent statistical analysis. Short-range interactions were evaluated using a Verlet neighbor list, while long-range electrostatics were treated with the particle-mesh Ewald (PME) method; bond lengths involving hydrogen atoms were constrained. Trajectory post-processing and the calculation of structural observables, including $R_g$, were performed using the built-in GROMACS gmx analysis tools, based on the trajectories collected after completion of the annealing procedure[4]. Molecular visualization and trajectory inspection were carried out using VMD[5].

**Supplementary Table 1**. Summary of relative molecular structure parameters of hyperbranched PS samples measured by SEC.

| Samples | Entry | $M_w{}^a/10^5$ (g/mol) | $M_n{}^a/10^5$ (g/mol) | $M_p{}^a/10^5$ (g/mol) | $\langle M_w/M_n \rangle^a$ |
|---|---|---|---|---|---|
| $H\text{-}PS_{1/25}$ | 1 | 0.19 | 0.09 | 0.39 | 2.14 |
| | 2 | 0.38 | 0.13 | 0.94 | 3.04 |
| | 3 | 0.77 | 0.16 | 3.15 | 4.83 |
| $H\text{-}PS_{1/95}$ | 4 | 0.57 | 0.25 | 1.15 | 2.29 |
| | 5 | 1.18 | 0.38 | 3.01 | 3.06 |
| | 6 | 3.22 | 0.47 | 13.12 | 6.89 |
| $H\text{-}PS_{1/170}$ | 7 | 0.49 | 0.32 | 0.75 | 1.51 |
| | 8 | 1.27 | 0.51 | 2.99 | 2.50 |
| | 9 | 2.73 | 0.68 | 9.03 | 4.01 |
| | 10 | 4.65 | 0.85 | 18.55 | 5.49 |
| $H\text{-}PS_{1/650}$ | 11 | 2.11 | 1.24 | 3.66 | 1.70 |
| | 12 | 2.30 | 0.73 | 6.39 | 3.18 |
| | 13 | 5.13 | 1.61 | 18.04 | 3.18 |

[a] Data are obtained by analyzing the SEC data based on the classical calibration method.

**Supplementary Table 2**. Summary of relative molecular structure parameters of linear PS samples measured by SEC.

| Samples | $M_w$[a]/$10^5$ (g/mol) | $M_n$[a]/$10^5$ (g/mol) | $M_p$[a]/$10^5$ (g/mol) | $<M_w/M_n>$[a] | $<R_g>$[b]/nm | $<C^*>$[c]/(g/L) |
|---|---|---|---|---|---|---|
| L-$PS_1$ | 0.96 | 0.4 | 0.6 | 2.39 | 11.2 | 26.5 |
| L-$PS_2$ | 2.8 | 1.3 | 2.6 | 2.15 | 21.2 | 11.4 |
| L-$PS_3$ | 5.8 | 2.7 | 7.1 | 2.12 | 32.6 | 6.5 |
| L-$PS_4$ | 7.4 | 3.3 | 5.8 | 2.23 | 37.7 | 5.4 |
| L-$PS_5$ | 9.8 | 3.4 | 5.0 | 2.89 | 44.5 | 4.3 |

[a] Data are obtained by analyzing the SEC data based on the classical calibration method.
[b] Data are estimated based on the scaling relation of $R_g = 1.20 \times 10^{-2} M_w^{0.595}$ in toluene[6].
[c] Data are estimated based on the scaling relation of $C^* = 2.11 \times 10^{-2} M_w^{-0.782}$ in toluene[6].

**Supplementary Table 3**. Summarized information of nanochannels with different average pore sizes calculated based on scanning electron microscopy characterization and Poiseuille equation

| Series | $D_{SEM,F}$[a]/ nm | $D_{SEM,B}$[a] /nm | $[(D_{SEM,F}+D_{SEM,B})/2]$[a] /nm | $Đ$[a] | $N$[a]/ ($10^{10}$pores/$cm^2$) | $D_{Poiseuille}$[b]/nm | $Q_{micro}$[c]/ ($10^{-21}$ $m^3$/s) | $v_{micro}$[c]/ ($10^{-6}$ m/s) |
|---|---|---|---|---|---|---|---|---|
| $D_1$ | 30 | 28 | 29 | 1.02 | 2.71 | 28 | 2.06 | 3.35 |
| $D_2$ | 40 | 36 | 38 | 1.01 | 2.62 | 32 | 2.11 | 2.62 |
| $D_3$ | 46 | 44 | 45 | 1.01 | 2.42 | 41 | 2.31 | 1.75 |
| $D_4$ | 68 | 64 | 66 | 1.01 | 1.02 | 55 | 5.44 | 2.29 |
| $D_5$ | 78 | 76 | 77 | 1.01 | 0.87 | 72 | 6.39 | 1.57 |
| $D_6$ | 82 | 80 | 81 | 1.00 | 0.71 | 88 | 7.83 | 1.29 |
| $D_7$ | 96 | 94 | 95 | 1.00 | 0.62 | 96 | 8.97 | 1.24 |

[a] Data were obtained by analyzing the scanning electron microscope images using the IMAGE-J software.
[b] Data were obtained by analyzing the transmembrane pressure using the Poiseuille equation.
[c] Data are estimated based on the macroscopic flow rate $Q$ = 0.2 mL/h.

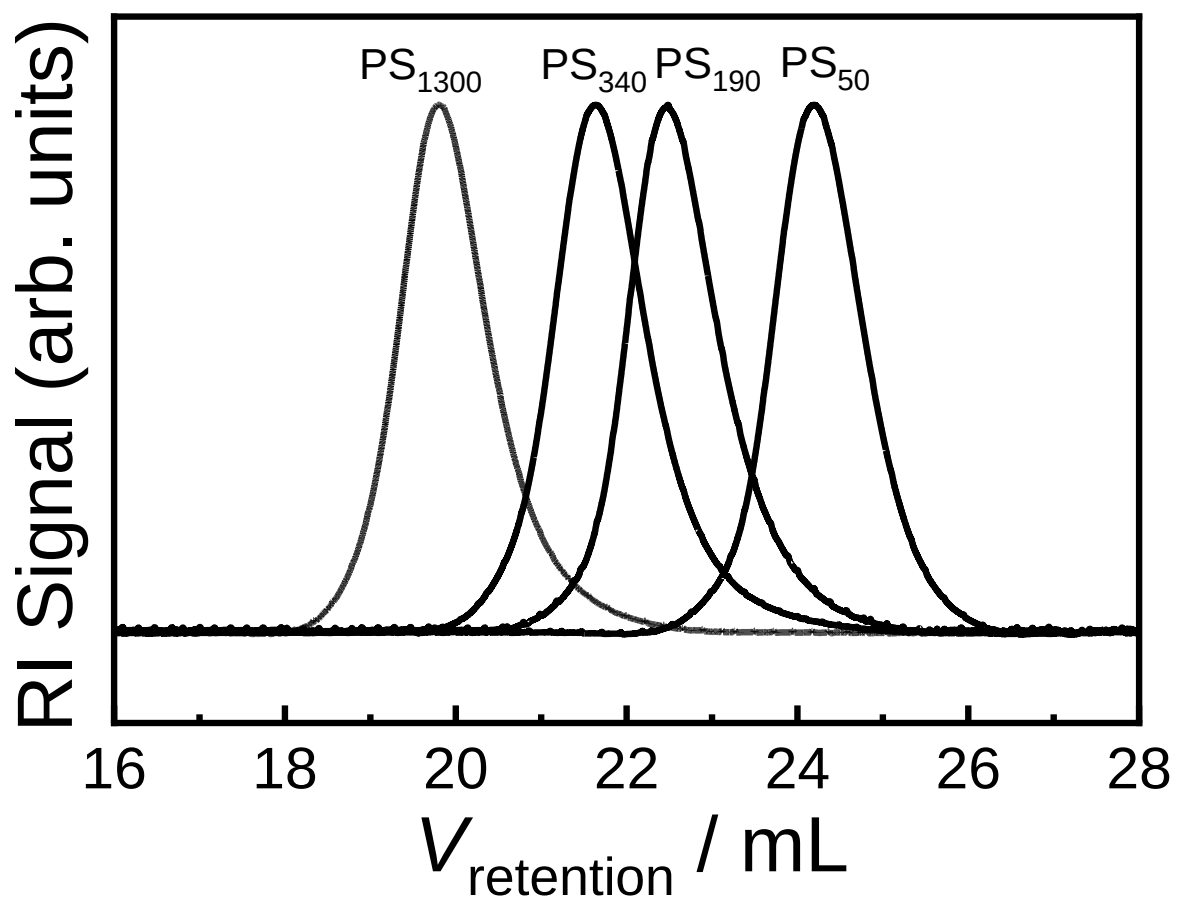


**Supplementary Fig. 1.** Size exclusion chromatography (SEC) curves of prepared $AB_2$-type $PS_{1300}$, $PS_{340}$, $PS_{190}$ and $PS_{50}$ macromonomers. SEC used tetrahydrofuran as the mobile phase at 1.0 mL $min^{-1}$.

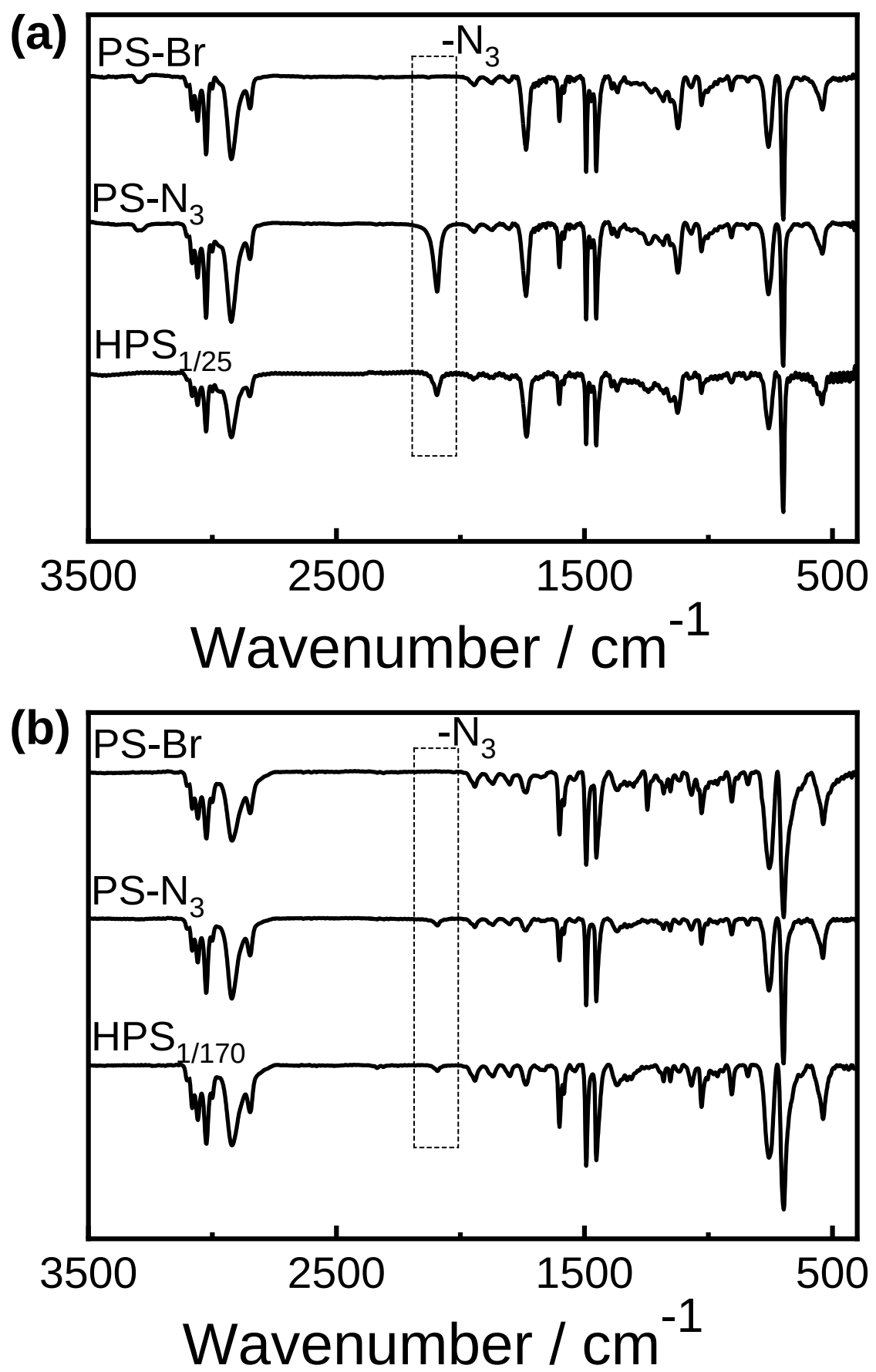


**Supplementary Fig. 2.** FTIR spectra of bromine and azide functionalized macromonomers, and hyperbranched polymers corresponding to **a** $PS_{50}$ and **b** $PS_{340}$.

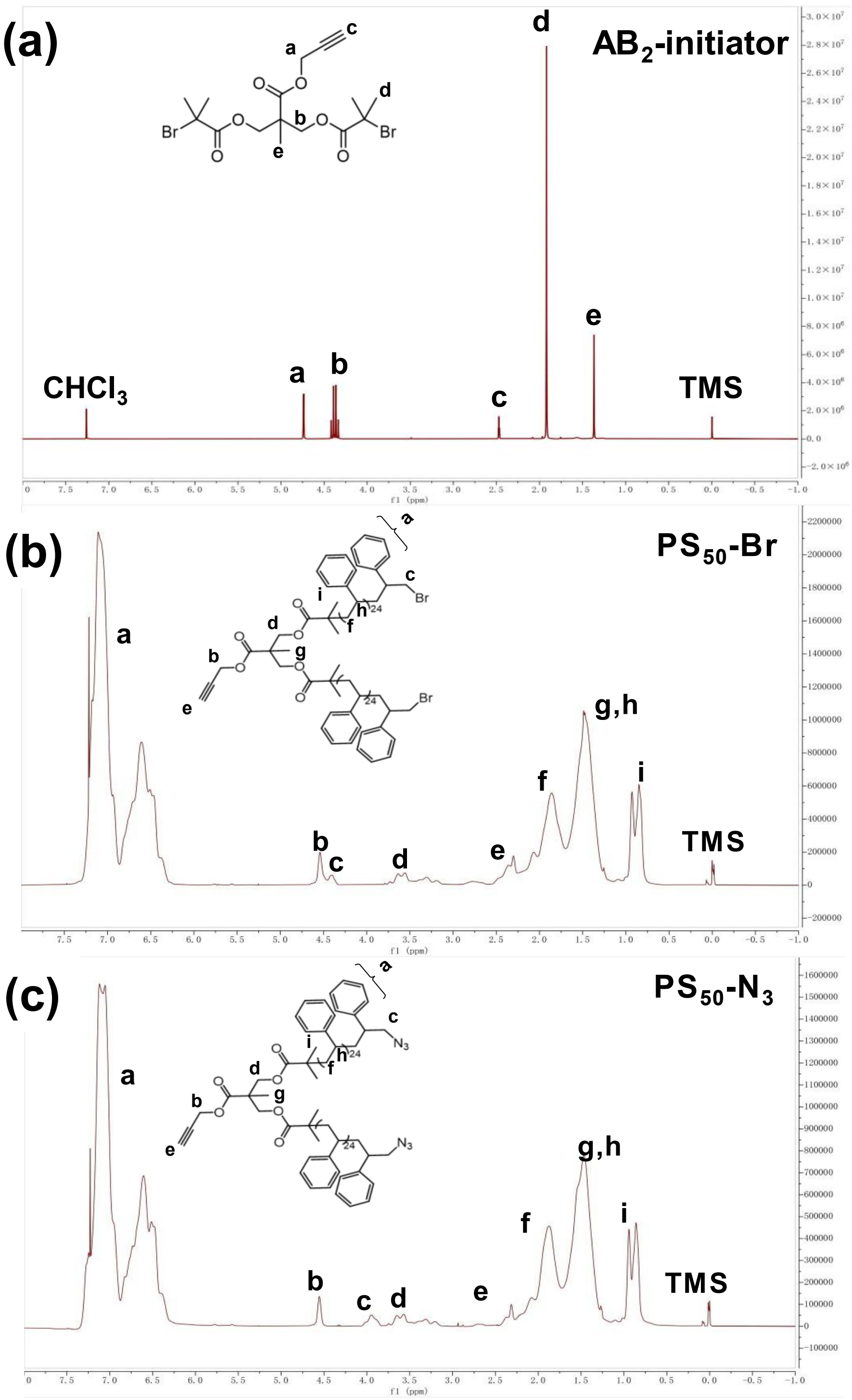


**Supplementary Fig. 3.** $^{1}$H NMR spectra (400 MHz, $CDCl_3$ containing 0.03% TMS) of **a** $AB_2$ initiator; **b** bromine and **c** azide functionalized macromonomers corresponding to $PS_{50}$.

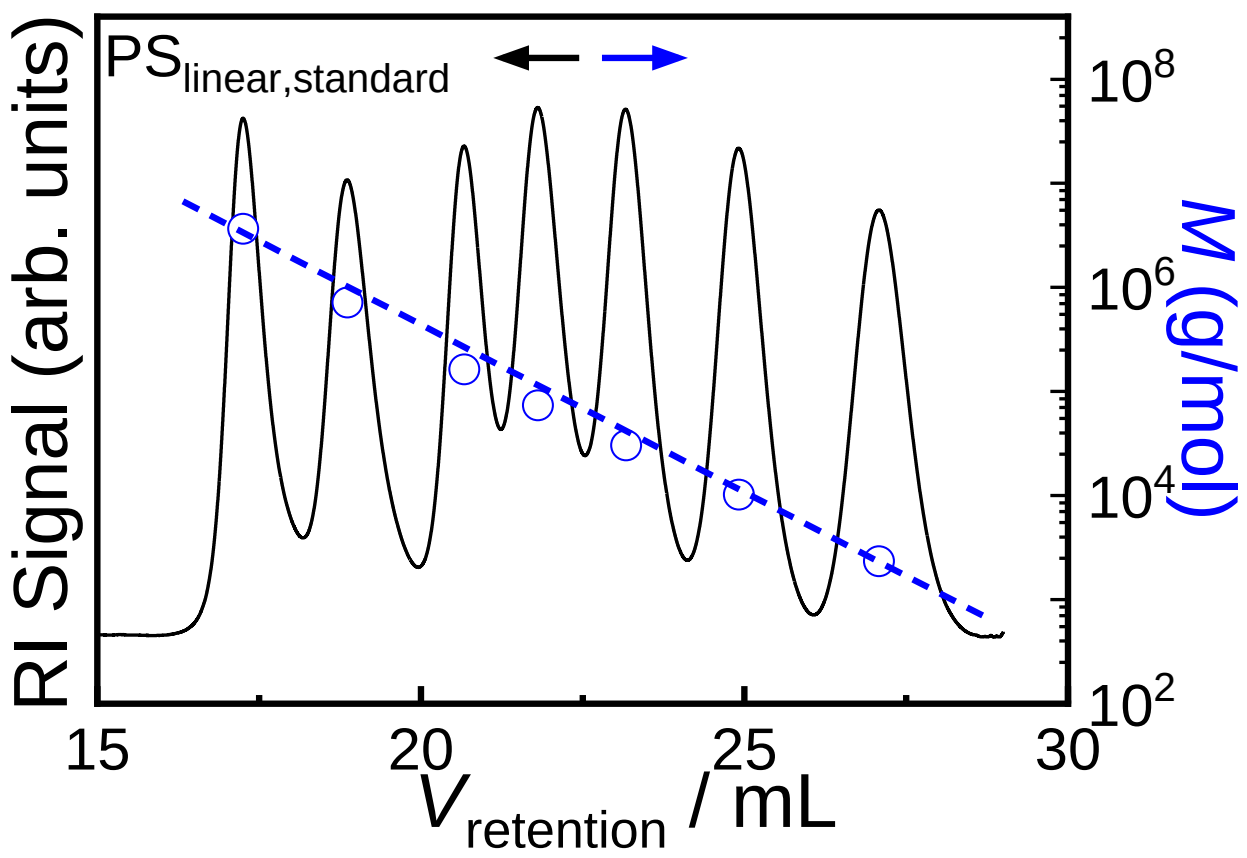


**Supplementary Fig. 4.** SEC curves of standard mixtures of linear polystyrene standard samples ($M_w$ = 3,500,000, 696,000, 156,000, 68,000, 28,500, 9,460, and 2,050 g/mol) (black solid line) and the molecular weight calibration curve (blue dashed line). SEC used tetrahydrofuran as the mobile phase at 1.0 mL min$^{-1}$.

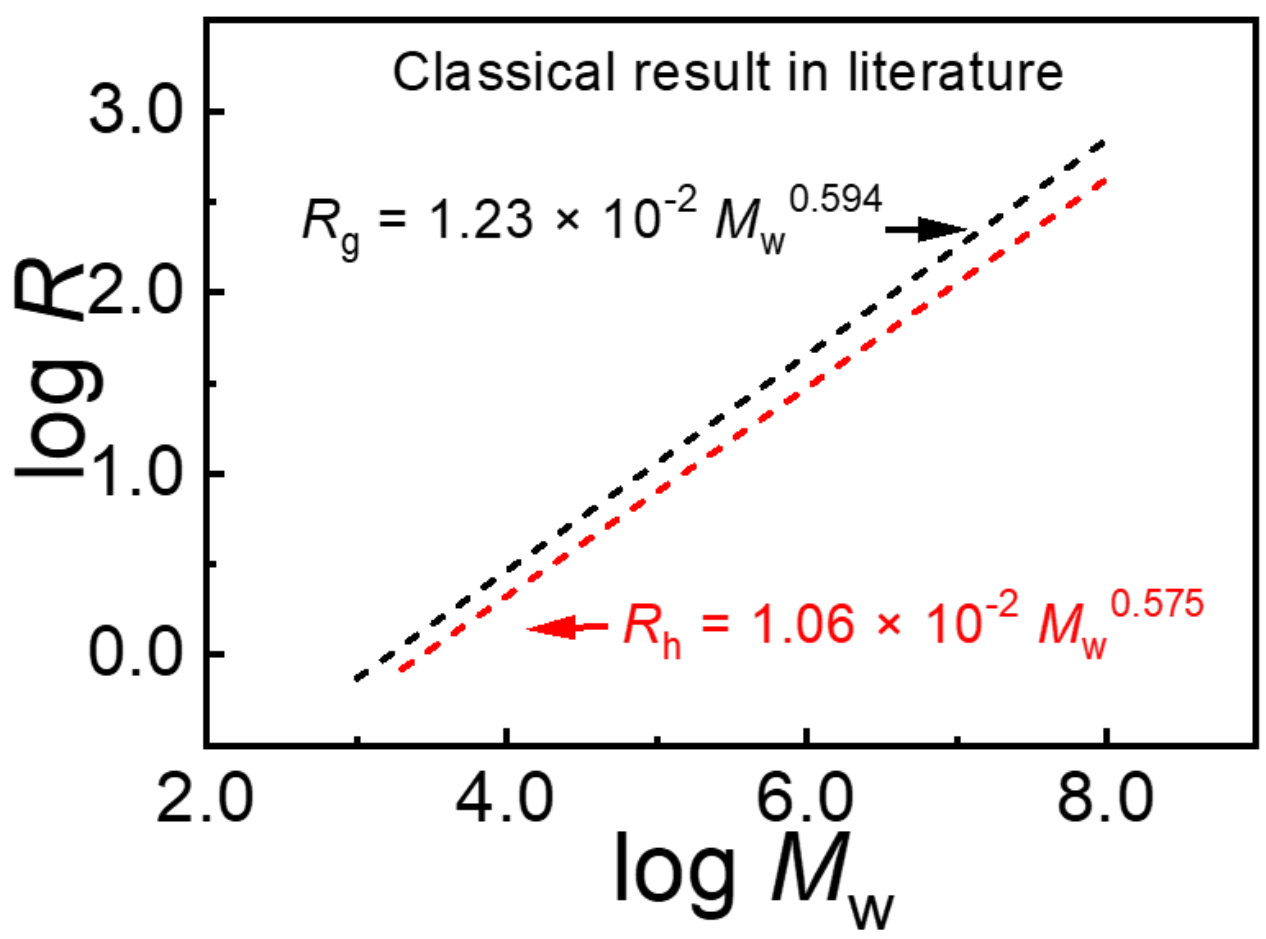


**Supplementary Fig. 5.** Scaling laws between the radius of gyration ($R_g$) and hydrodynamic radius ($R_h$) with weight-average molecular weight ($M_w$) for polystyrene in toluene[6].

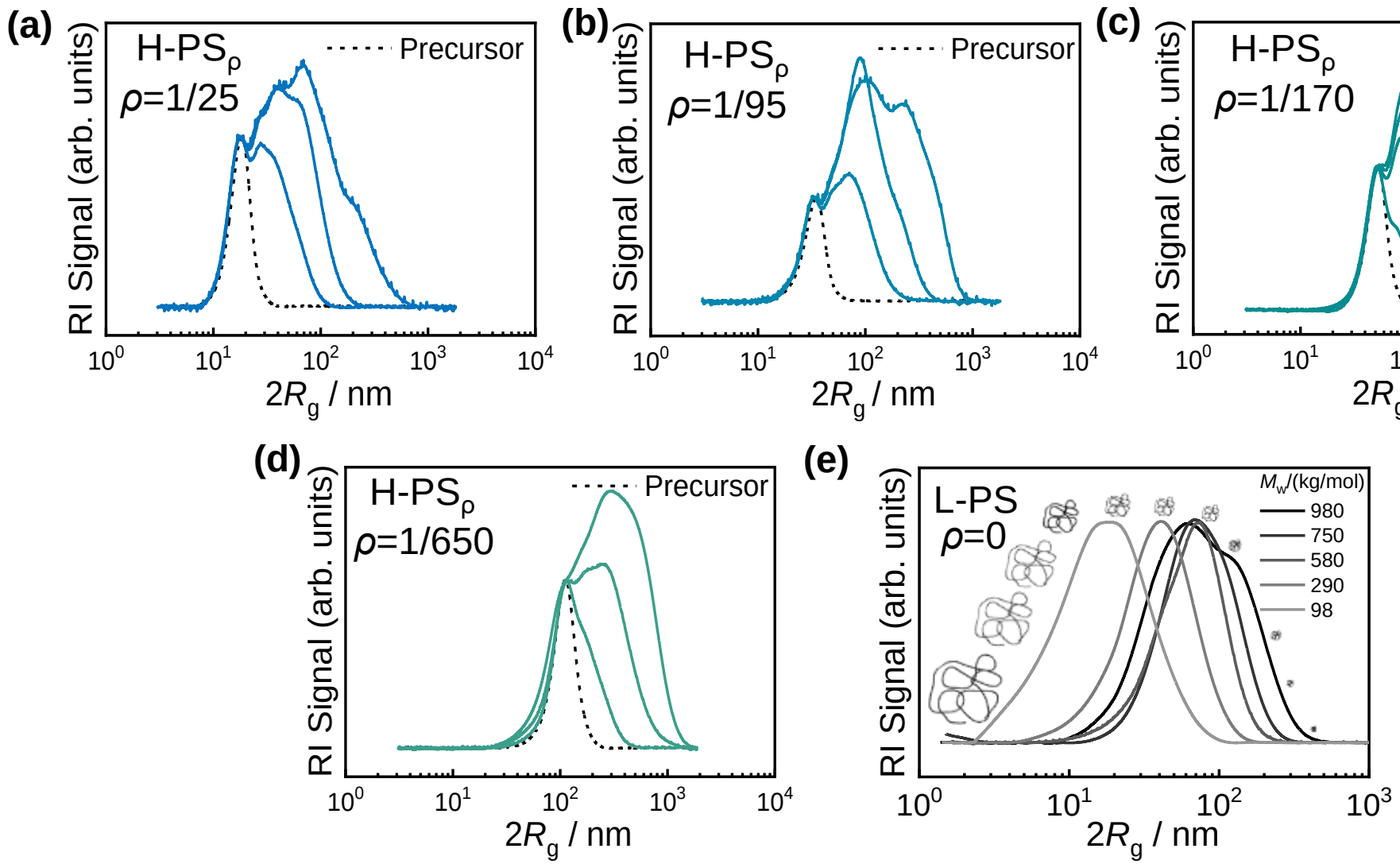


**Supplementary Fig. 6.** Apparent radius of gyration ($R_{g,app}$) profiles for polystyrene samples with varying branching densities: **a** $\rho$ = 1/25, **b** $\rho$ = 1/95, **c** $\rho$ = 1/170, **d** $\rho$ = 1/650 and **e** $\rho$ = 0.

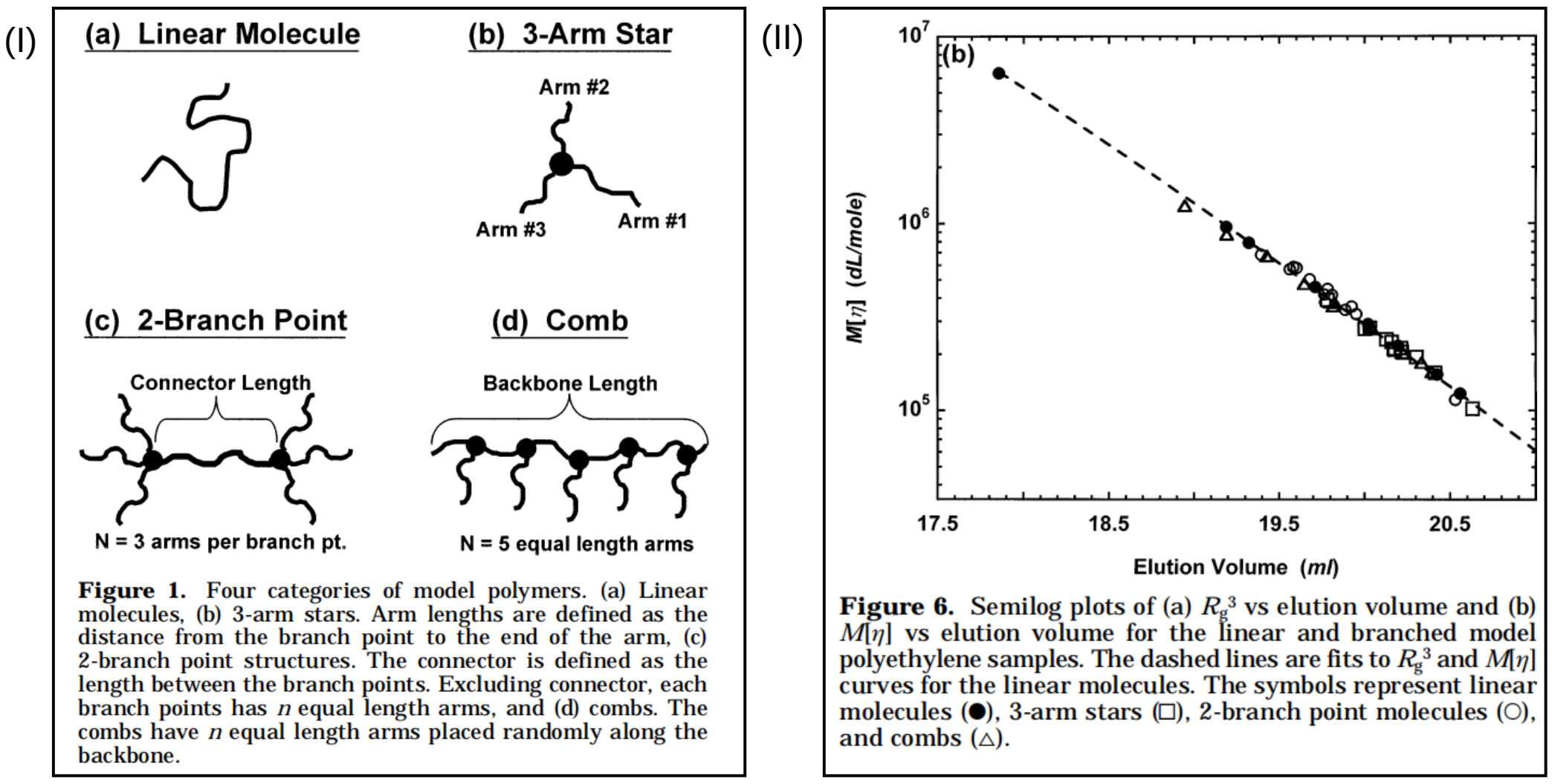


**Supplementary Fig. 7.** (I) Schematic representations of four categories of model polymers: **a** linear molecules, **b** 3-arm stars, **c** 2-branch point structures, and **d** combs. (II) Semilog plots of $M[\eta]$ versus elution volume for linear and branched model polyethylene samples. The dashed line represents the fit to the $M[\eta]$ curve for the linear molecules. The symbols represent linear molecules (solid circles), 3-arm stars (open squares), 2-branch point molecules (open circles), and combs (open triangles). All figures are reproduced with permission from Ref. [7]. Copyright 2004 American Chemical Society.

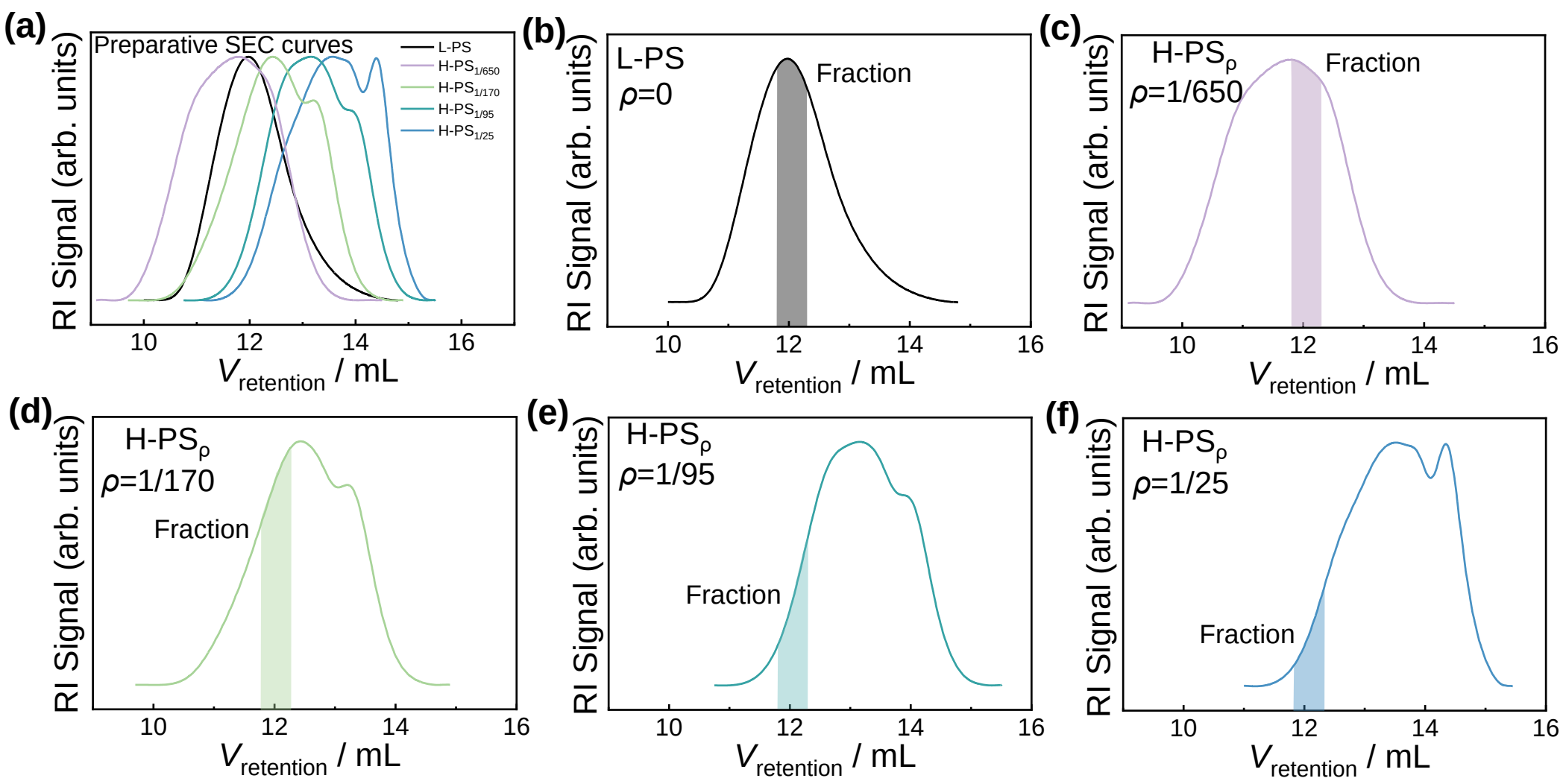


**Supplementary Fig. 8.** Preparative SEC fractionation of linear and hyperbranched polystyrene samples. **a** Overall preparative SEC elution profiles of the broadly distributed linear PS (L-PS) and four hyperbranched variants with varying branching densities (H-PS$_{1/650}$, H-PS$_{1/170}$, H-PS$_{1/95}$, and H-PS$_{1/25}$). **b-f** Isolation of specific fractions (indicated by the shaded regions) collected within an identical co-elution window (retention volume $V_{retention}$ = 11.8-12.3 mL, corresponding to the 11.8-12.3 min elution window at a flow rate of $Q$ = 5 mL/min, with tetrahydrofuran as the mobile phase) for L-PS and all hyperbranched variants.

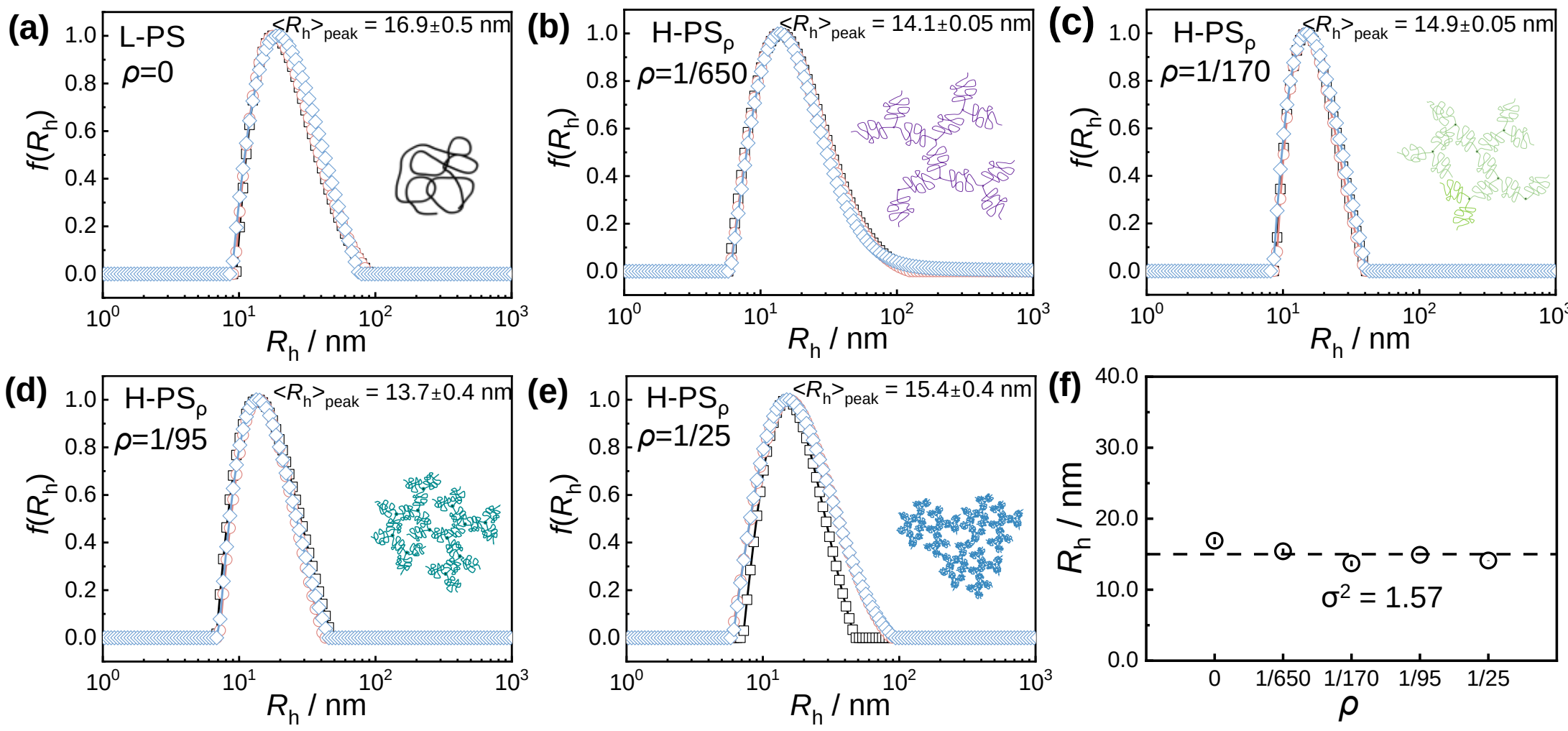


**Supplementary Fig. 9.** Dynamic light scattering (DLS) measurements of the co-eluting fractions captured within the same elution window of 11.8-12.3 min. **a-e** Hydrodynamic radius ($R_h$) distributions for fractions obtained from the five topological systems: linear PS and four hyperbranched variants (H-$PS_{1/650}$, H-$PS_{1/170}$, H-$PS_{1/95}$, and H-$PS_{1/25}$). **f** Summary of the peak $R_h$ values as a function of branching density ($\rho$). The dashed horizontal line denotes the overall average $R_h$ value of 15 nm across all samples, confirming that these co-eluting fractions possess indistinguishable and closely matched $R_h$ within experimental error (±2 nm) regardless of their topological architectures. The variance ($\sigma^2$) of the peak $R_h$ values across the five samples is 1.57.

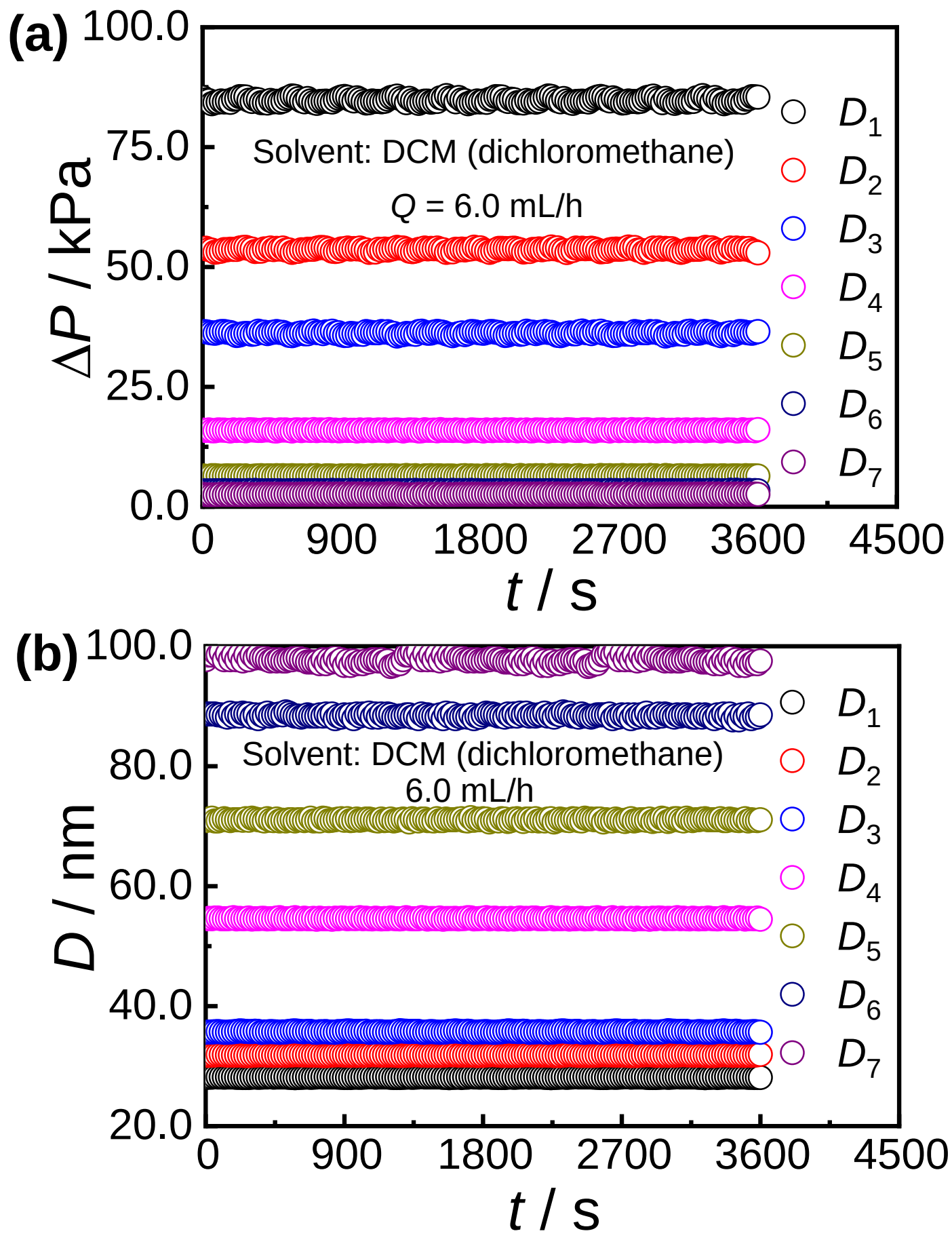


**Supplementary Fig. 10.** **a** Transmembrane pressure ($\Delta P$) versus time ($t$) for seven membranes with different pore sizes (AAO $D_1$-$D_7$) using dichloromethane (DCM) as the solvent at a flow rate ($Q$) of 6.0 mL/h. **b** Pore size ($D$) calculated via the Hagen-Poiseuille equation versus time ($t$) for the same membranes under identical solvent and flow rate conditions.

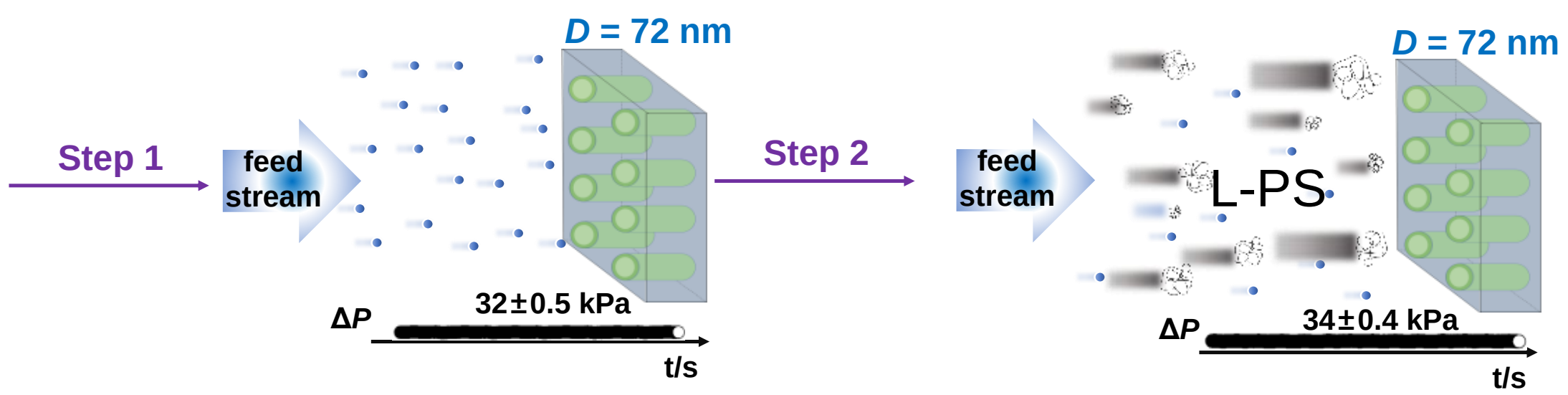


**Supplementary Fig. 11.** Sequential interception protocol (solvent→L-PS) with reverse solvent flushing to minimize polarization effects, where transmembrane pressure ($\Delta P$) remains stable (32±0.5 - 34±0.4 kPa) during experiments with $D$ = 72 nm AAO membranes, indicating membrane structural stability.

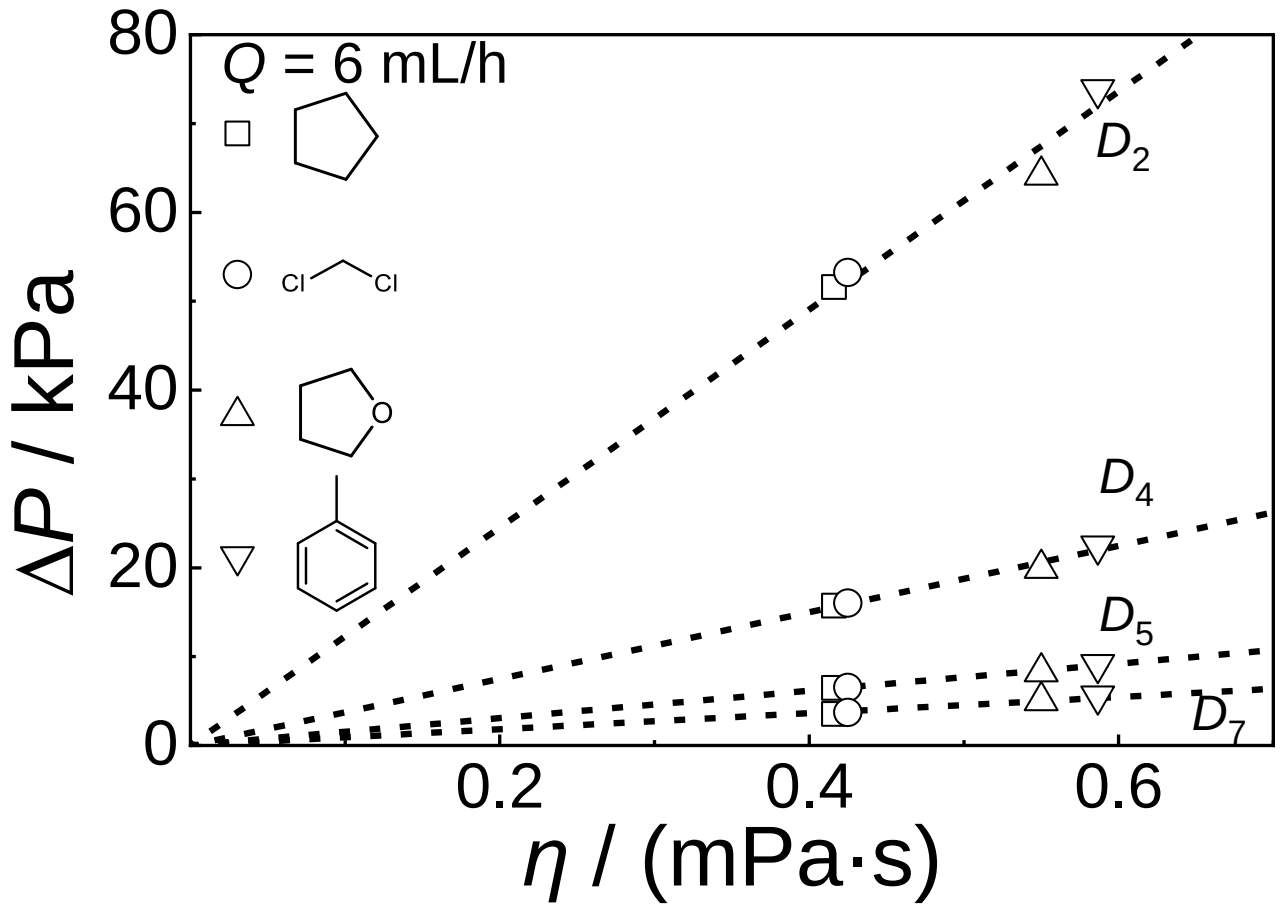


**Supplementary Fig. 12.** The solvent viscosity ($\eta$) dependence of the transmembrane pressure ($\Delta P$) at a fixed flow rate of 6 mL/h, for membranes with different pore sizes ($D_2$, $D_4$, $D_5$, $D_7$) and various solvents (as indicated by molecular structures).

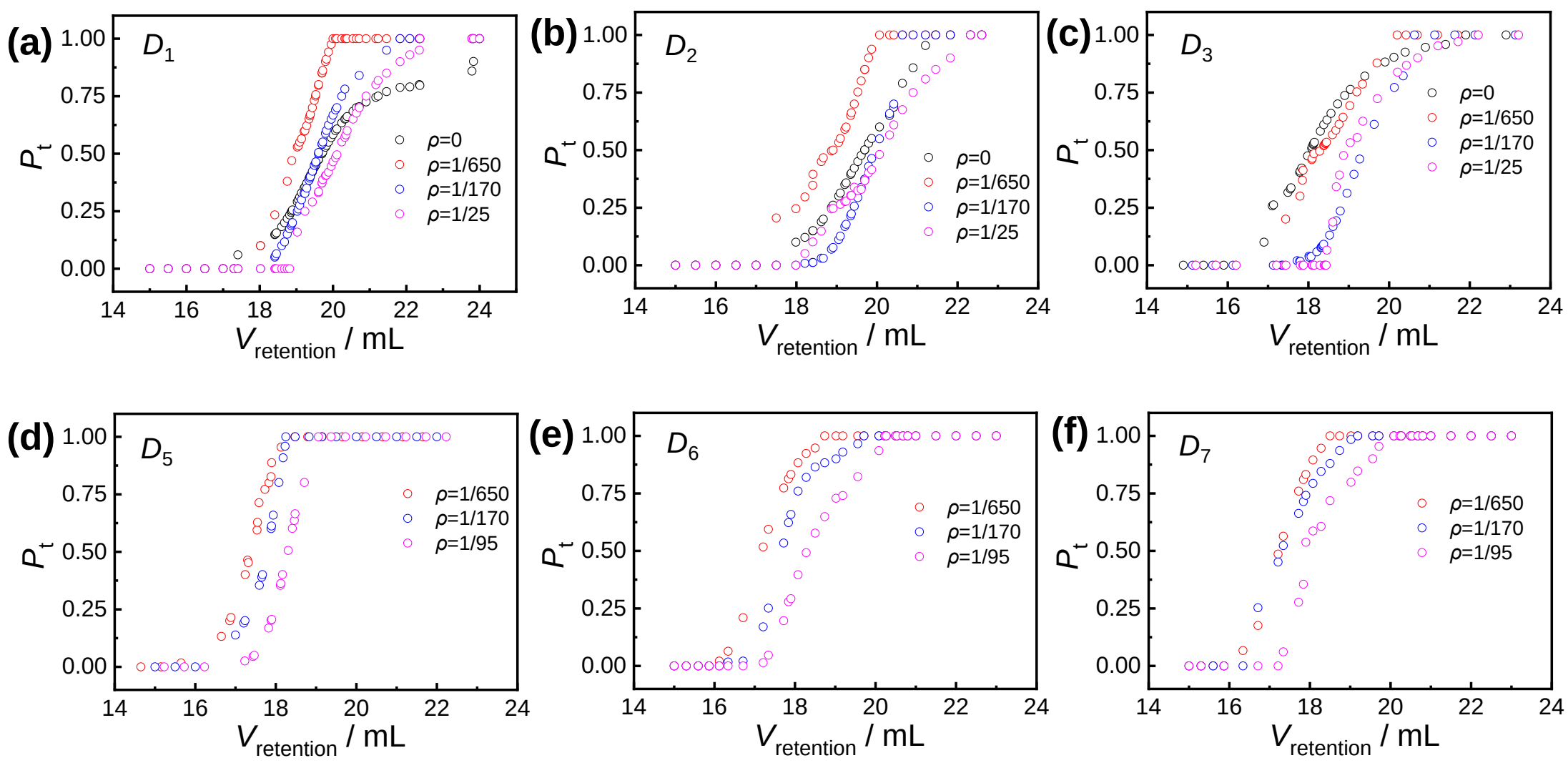


**Supplementary Fig. 13.** Dependence of translocation probability ($P_t$) on retention volume ($V_{retention}$) for nanopore $D_1$ - $D_3$ (**a-c**) and $D_5$ – $D_7$ (**d-f**).

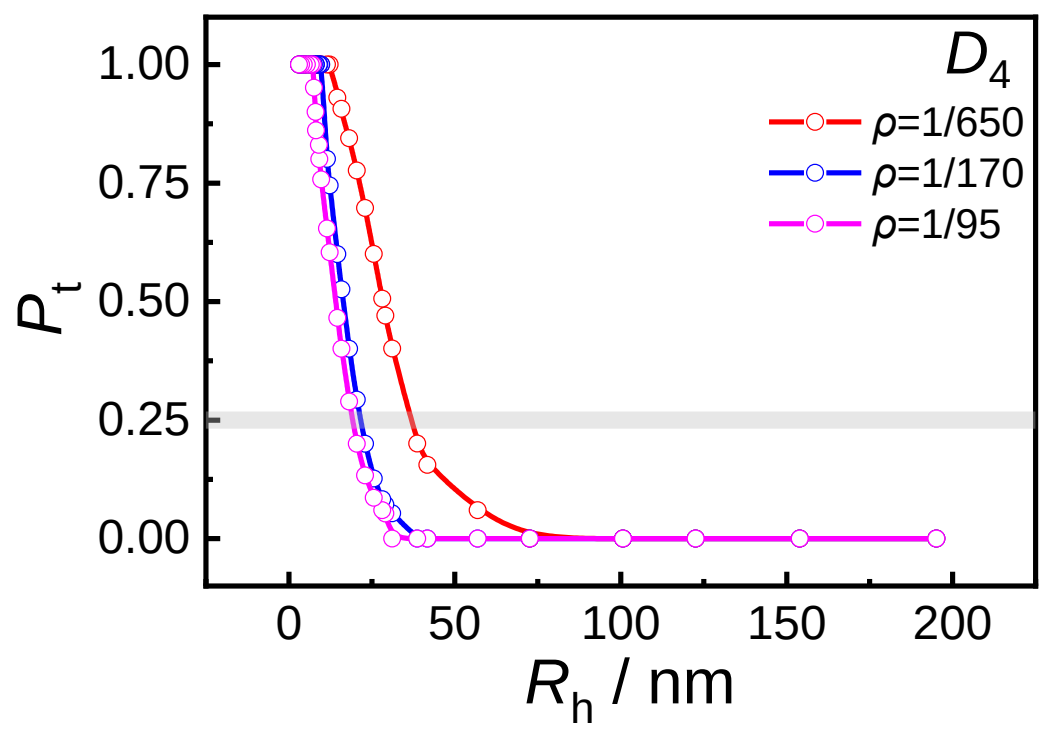


**Supplementary Fig. 14.** Dependence of translocation probability ($P_t$) on hydrodynamic radius ($R_h$) for nanopore $D_4$, where the intersection point of the gray band ($P_t = 0.25$) and the curve is $R_{h,c}$.

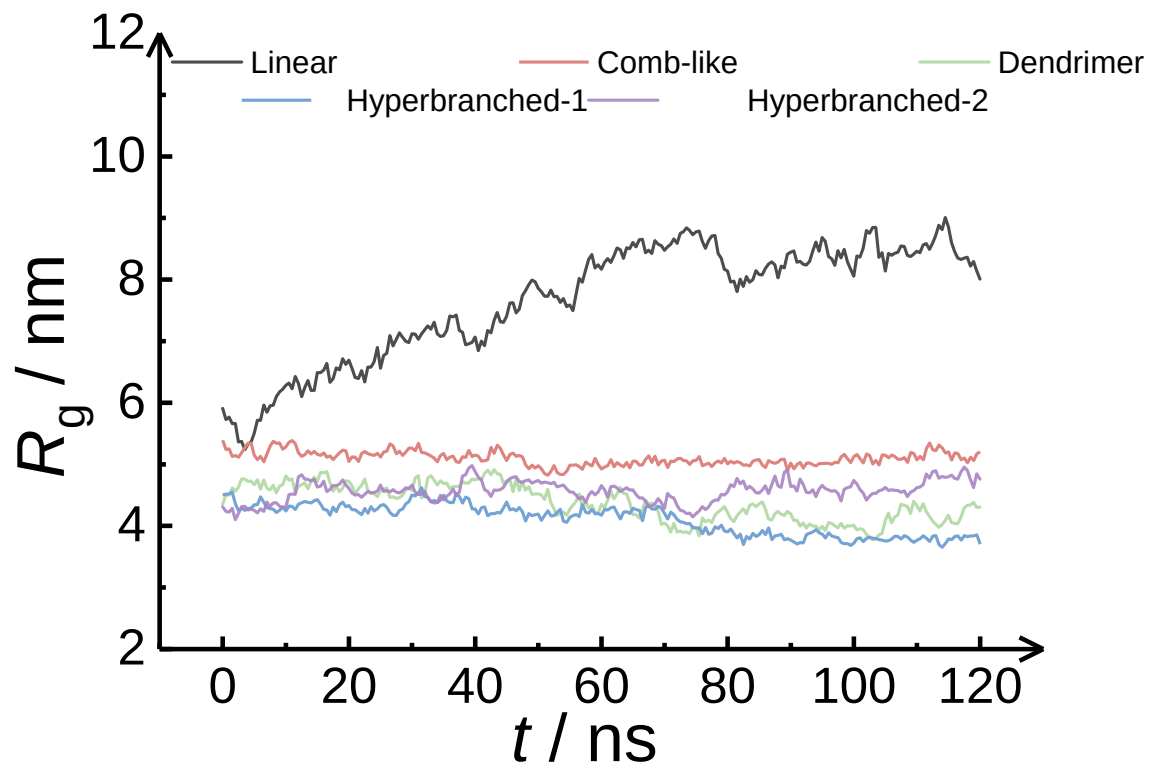


**Supplementary Fig. 15.** Time evolution of the radius of gyration ($R_g$) over 120 ns all-atom molecular dynamics simulations for the linear (black), comb-like (red), dendrimer (green), hyperbranched-1 (blue, H-$PS_{1/14}$), and hyperbranched-2 (purple, H-$PS_{1/7}$) polymer systems, each comprising 700 monomers.

**References**

(1) Li, L.; He, C.; He, W.; Wu, C. Formation Kinetics and Scaling of "Defect-Free" Hyperbranched Polystyrene Chains with Uniform Subchains Prepared from Seesaw-Type Macromonomers. *Macromolecules* **2011**, *44*, 8195-8206.

(2) Lu, T. Sobtop, version 1.0 (dev4); http://sobereva.com/soft/Sobtop (accessed Jan 30, 2026).

(3) Showalter, S. A.; Brüschweiler, R. Validation of Molecular Dynamics Simulations of Biomolecules Using NMR Spin Relaxation as Benchmarks: Application to the AMBER99SB Force Field. *J. Chem. Theory Comput.* **2007**, *3*, 961-975.

(4) Abraham, M. J.; Murtola, T.; Schulz, R.; Páll, S.; Smith, J. C.; Hess, B.; Lindahl, E. GROMACS: High performance molecular simulations through multi-level parallelism from laptops to supercomputers. *SoftwareX* **2015**, *1*, 19-25.

(5) Humphrey, W.; Dalke, A.; Schulten, K. VMD: Visual molecular dynamics. *J. Mol. Graph.* **1996**, *14*, 33-38.

(6) Fetters, L. J.; Hadjichristidis, N.; Lindner, J. S.; Mays, J. W. Molecular Weight Dependence of Hydrodynamic and Thermodynamic Properties for Well‐Defined Linear Polymers in Solution. *J. Phys. Chem. Ref. Data* **1994**, *23*, 619-640.

(7) Sun, T.; Chance, R. R.; Graessley, W. W.; Lohse, D. J. *Macromolecules* **2004**, *37*, 4304-4312.
