# Supplementary material for: Conformational entropy bottleneck in polymer nanopore interception driven by branching architecture and excluded volume
Source: Nat Commun. 2026 Sep 29;17:11231. doi: 10.1038/s41467-026-78055-1 (PMC13720087; doi:10.1038/s41467-026-78055-1)
Supplement: Supplementary file 2 [file 41467_2026_78055_MOESM2_ESM.pdf]

nature portfolio

Peer Review File

Conformational Entropy Bottleneck in Polymer Nanopore Interception Driven by Branching Architecture and Excluded Volume

Corresponding Author: Professor Lianwei Li

This file contains all reviewer reports in order by version, followed by all author rebuttals in order by version.

Attachments originally included by the reviewers as part of their assessment can be found at the end of this file.

Version 0:

Reviewer comments:

Reviewer #1

(Remarks to the Author)
This manuscript by Wang et al. presents an integrated approach combining experiments, computational and theoretical analysis to investigate the role of polymer architecture—specifically, branching—and excluded volume on the interception of polymers by nanopores in the weak-compression limit (i.e., at ultra-low flows where diffusion dominates). Using well-defined anodic aluminum oxide nanopores, broadly distributed linear and hyperbranched polystyrene samples, molecular dynamics (MD) simulations, and scaling analysis, the authors report a topology-dependent scaling relationship for the critical interception size $R_{h,c} \sim \rho^{-1/2}$. The authors demonstrate that hyperbranched chains are intercepted at significantly smaller relative pore sizes compared to their linear counterparts. Combining the experimental size-exclusion chromatography (SEC) protocols with the observations from MD simulations, the study proposes a "conformational entropy bottleneck" mechanism where the restricted conformational fluctuations of branched polymers impose a higher free-energy barrier for pore entry.

The manuscript is generally clear and understandable, and the methodology appears robust. However, there are a few points as mentioned below where the theoretical justification and discussion could be improved to strengthen the paper's impact and rigor.

1. Hydrodynamic sizes are derived from SEC calibrated with linear polystyrene standards. This may introduce systematic, topology-dependent bias for hyperbranched polymers. Since the main conclusions rely on comparing sizes across branching densities, the robustness of this approach should be justified.

2. The experimental finding that the critical hydrodynamic interception size scales as $R_{h,c} \sim \rho^{-1/2}$ is a central highlight of the paper. While the experimental data fit this beautifully, the theoretical framework provides qualitative support but does not rigorously derive the exponent. The authors should either strengthen the derivation or present the scaling more cautiously as an empirical observation. Also, providing a physical basis for this exponent will greatly elevate the theoretical impact of the paper.

3. Simulations convincingly show reduced conformational fluctuations with increasing branching, supporting the proposed mechanism. However, a more direct quantitative link to $R_{h,c}$ or the observed scaling would strengthen the manuscript.

4. In the SI, the authors mention the 700-mer polystyrene chains subjected to 100 ns of cyclic annealing. While cyclic annealing is a valid strategy to overcome local minima, 100 ns may not be enough for a polymer of this size and topological complexity to achieve global equilibration. The authors should provide supplementary data such as Rg or end-to-end distances demonstrating that the chains are indeed fully equilibrated.

5. Uncertainty in Pt (±3%) is reported but not propagated to $R_{h,c}$. What uncertainty was considered while defining blue region? Also, in Figures depicting the translocation probability (Pt) vs. critical retention volume or hydrodynamic radius, please ensure that the method used to calculate the error bars is explicitly stated in the relevant figure captions.

6. In Figure 7, It is not clear where the FWHM comes from? Is it from Rg distribution? It should be clearly mentioned in captions and the distributions should be provided. Df is also not defined and what does it signify?

7. Methods section should be revised to add more technical details to ensure reproducibility.

Reviewer #2

(Remarks to the Author)
I co-reviewed this manuscript with one of the reviewers who provided the listed reports. This is part of the Nature Communications initiative to facilitate training in peer review and to provide appropriate recognition for Early Career Researchers who co-review manuscripts.

Reviewer #3

(Remarks to the Author)
The authors submitted a manuscript the effect of (hyper-)branching of polystyrene (PS) polymers on the transport through nanopores. They observe a distinct power-law dependency between the critical interception size and the branch density. Though the work seems sound and it can be of interest to the polymer physics community, I think the is not of interest to the more generic audience of Nature Communications. Also, the novelty of the work is too minor compared to existing work - by the authors and by other authors - on the effect of PS polymer branching on the flow through nanopores. See for example, https://doi.org/10.1021/ma301468z, https://doi.org/10.1039/C0PY00361A
Therefore, I do not recommend this work to be considered for Nature Communications. Instead, I recommend that the authors submit the work to a specialized journal such as ACS Macromolecules.

Reviewer #4

(Remarks to the Author)
attached.

Version 1:

Reviewer comments:

Reviewer #1

(Remarks to the Author)
The authors have provided substantial evidence and made substantial changes to the manuscript. The comments of this reviewer have been addressed.

The authors have also adequately addressed the comments of Reviewer 4.

Reviewer #2

(Remarks to the Author)
I co-reviewed this manuscript with one of the reviewers who provided the listed reports. This is part of the Nature Communications initiative to facilitate training in peer review and to provide appropriate recognition for Early Career Researchers who co-review manuscripts.

Open Access This Peer Review File is licensed under a Creative Commons Attribution 4.0 International License, which permits use, sharing, adaptation, distribution and reproduction in any medium or format, as long as you give appropriate credit to the original author(s) and the source, provide a link to the Creative Commons license, and indicate if changes were made.
In cases where reviewers are anonymous, credit should be given to 'Anonymous Referee' and the source.
The images or other third party material in this Peer Review File are included in the article's Creative Commons license, unless indicated otherwise in a credit line to the material. If material is not included in the article's Creative Commons license and your intended use is not permitted by statutory regulation or exceeds the permitted use, you will need to obtain permission directly from the copyright holder.
To view a copy of this license, visit https://creativecommons.org/licenses/by/4.0/

<u>To the comments of reviewer 1</u>:

This manuscript by Wang et al. presents an integrated approach combining experiments, computational and theoretical analysis to investigate the role of polymer architecture—specifically, branching—and excluded volume on the interception of polymers by nanopores in the weak-compression limit (i.e., at ultra-low flows where diffusion dominates). Using well-defined anodic aluminum oxide nanopores, broadly distributed linear and hyperbranched polystyrene samples, molecular dynamics (MD) simulations, and scaling analysis, the authors report a topology-dependent scaling relationship for the critical interception size Rh,c∼ρ−1/2. The authors demonstrate that hyperbranched chains are intercepted at significantly smaller relative pore sizes compared to their linear counterparts. Combining the experimental size-exclusion chromatography (SEC) protocols with the observations from MD simulations, the study proposes a "conformational entropy bottleneck" mechanism where the restricted conformational fluctuations of branched polymers impose a higher free-energy barrier for pore entry.

The manuscript is generally clear and understandable, and the methodology appears robust. However, there are a few points as mentioned below where the theoretical justification and discussion could be improved to strengthen the paper's impact and rigor.

Reply: We sincerely appreciate your thoughtful and constructive comments on our manuscript. We have carefully considered each point raised and made the necessary revisions to strengthen our manuscript further. We believe that these enhancements will improve the rigor and impact of our findings.

1) **Question:** Hydrodynamic sizes are derived from SEC calibrated with linear polystyrene standards. This may introduce systematic, topology-dependent bias for hyperbranched polymers. Since the main conclusions rely on comparing sizes across branching densities, the robustness of this approach should be justified.

   Reply: We thank you for raising this fundamental and important question regarding the validity of using linear polystyrene standards to calibrate the hydrodynamic radius of hyperbranched polymers. We fully agree that this is a critical issue for the interpretation of our data. Below we provide a comprehensive response, grounded in **both classical literature and our own rigorous experimental validations**.

   a) **Classical SEC Theory Establishes Hydrodynamic Volume as the Universal Separation Principle**

      Since the seminal work of Grubisic, Rempp, and Benoit (1967), **it has been firmly established that size exclusion chromatography separates polymers based on their hydrodynamic volume, $V_h \sim [\eta]M$**, regardless of polymer species or chain architecture (linear, star, comb, etc.) [<u>Grubisic, Z.; Rempp, P.; Benoit, H. J. *Polym. Sci. B Polym. Lett.* **1967**, *5,* 753.</u>]. This "universal calibration" concept is the cornerstone of modern SEC.

      More importantly, Teraoka (<u>Teraoka, I. *Macromolecules* **2004**, *37,* 6632.</u>) provided a rigorous theoretical foundation by calculating the partition coefficients of polymers with various topologies (asymmetric stars, two-branch-point, combs) in slit-like pores. He demonstrated unequivocally that **the hydrodynamic radius R_h, not the radius of gyration R_g, is the correct molecular dimension that dictates the partitioning behavior**. Specifically, when the partition coefficient K was plotted against R_h/D (pore size ratio), all data for different architectures collapsed onto a single master curve. In contrast, plotting against R_g/D resulted in significant deviations. Since R_h is directly proportional to the cube root of the hydrodynamic volume ($R_h \sim ([\eta]M)^{1/3}$), Teraoka's work provides the theoretical basis for why SEC retention volume universally correlates with R_h.

Thus, using SEC retention volume to determine $\boldsymbol{R_h}$ **is not an empirical approximation but a physically grounded method, valid for linear and branched polymers alike.**

b) **Reported Experimental Evidence for Branched Polymers Follows the Same Principle**

Sun et al. (Sun, T.; et al. *Macromolecules* **2004**, *37*, 4304.) conducted an extensive SEC study on linear and branched polyethylenes (3-arm stars, H-shaped, and combs). Their key finding is highly relevant to our work: **For branched polymers, the elution volume satisfactorily correlates with** $[\eta]M$ **(and thus** R_h**), as shown below (Figure R1)**. In contrast, branched polymers deviated strongly from the linear R_g curve but remained on the $[\eta]M$ curve. **This directly demonstrates that for non-linear architectures, hydrodynamic volume (or** R_h**) is the sole determinant of SEC elution.**

Furthermore, Wang et al. (Wang, Y.; et al. *Macromolecules* **2011**, *44*, 403.) extended the theoretical analysis of "mean span dimension" and showed that for branched polymers, the reduction in size relative to linear chains is much milder for R_h and span dimension than for $\boldsymbol{R_g}$[4]. This is because branching primarily compresses the distal segments (affecting $\boldsymbol{R_g}$) but has a less dramatic effect on the core density (affecting R_h). **Consequently,** $\boldsymbol{R_h}$ **remains a robust and topology-independent metric for SEC calibration, even for highly branched structures like our hyperbranched PS.**

c) **Our Experimental Validation Confirms No Topology-Induced Bias**

Despite the strong literature support, we recognized that hyperbranched polymers with extremely high branching density (e.g., H-PS$_{1/25}$) might push the limits of conventional universal calibration. **Therefore, we performed three independent experimental validations in the revised manuscript (see Figures R2-R4 and associated discussion).**

- **Co-elution + Offline DLS (Data for Publication)**: We performed high-precision preparative SEC (Waters Ultrastyragel Linear THF, 19×300 mm, Q = 5 mL/min) to systematically fractionate both the broadly distributed linear PS (L-PS) and all four hyperbranched variants (H-PS$_{1/25}$, H-PS$_{1/95}$, H-PS$_{1/170}$, and H-PS$_{1/650}$), as shown in **Figure R2**. Samples were meticulously collected in 0.5-minute slice intervals. By deliberately shifting the collection windows based on their respective broad elution profiles, we successfully isolated series of narrow-distribution fractions. **Crucially, this protocol allowed us to obtain highly valuable "co-eluting matrices"—fractions of vastly different architectural densities that eluted at the exact same retention time window**. In the specific interception window of 11.8-12.3 min, we successfully captured fractions from all five topological systems (L-PS F3, H-PS$_{1/25}$ F1, H-PS$_{1/95}$ F2, H-PS$_{1/170}$ F3, and H-PS$_{1/650}$ F6). **DLS measurements confirmed that these fractions have indistinguishable** $\boldsymbol{R_h}$ **within experimental error (±2 nm), as shown in Figure R3.**
- **Quadruple-detector SEC (TD-SEC) (Data for Review Only)**: To provide ultimate theoretical closure, these fractionated samples were further subjected to quadruple-detector SEC (TD-SEC). By plotting the product of intrinsic viscosity and absolute molecular weight ($M[\eta]$, strictly proportional to hydrodynamic volume) against the retention volume ($V_{retention}$), **all data points from both linear and hyperbranched variants impeccably collapsed onto a single master curve (Benoit's Universal Calibration), as shown in Figure R4**. Together, these multi-dimensional fractionations and absolute scattering verifications provide direct, unambiguous physical evidence that the SEC retention volume in our system is a pure function of hydrodynamic size, devoid of topological bias. Consequently, calibrating the R_h of our hyperbranched systems using linear standards is both

physically sound and quantitatively highly reliable. Considering the importance of our experimental result, we respectfully request permission to publish the TD-SEC result elsewhere.

- **Shape Factor Analysis (Data for Review Only)**: Single-concentration static/dynamic light scattering on co-eluted fractions gave $R_g/R_h \approx 1.42$ for linear PS and ≈ 1.08 for hyperbranched PS (H-PS~1/650), confirming the expected coil-to-globule compaction. Absolute concentration was not determined, because accurate concentration determination is difficult for SEC-fractionated samples; the shape factor itself is robust in the dilute regime. **These data (Figure R5) are provided for reviewers only and will not appear in the published manuscript.**

d) Revisions in the Manuscript

We have revised the description and analysis of **page 6 line 18** to **page 7 line 7** of the manuscript as follows: "It is well-established by rigorous theoretical calculations that SEC separation is universally governed by hydrodynamic volume (or R_h), rather than R_g, irrespective of chain topology. … To experimentally verify that topological differences do not introduce systematic bias in our R_h determination, preparative SEC fractionation and offline DLS were performed on co-eluting matrices (**Figures S8** and **S9**), confirming precise physical size equivalence."

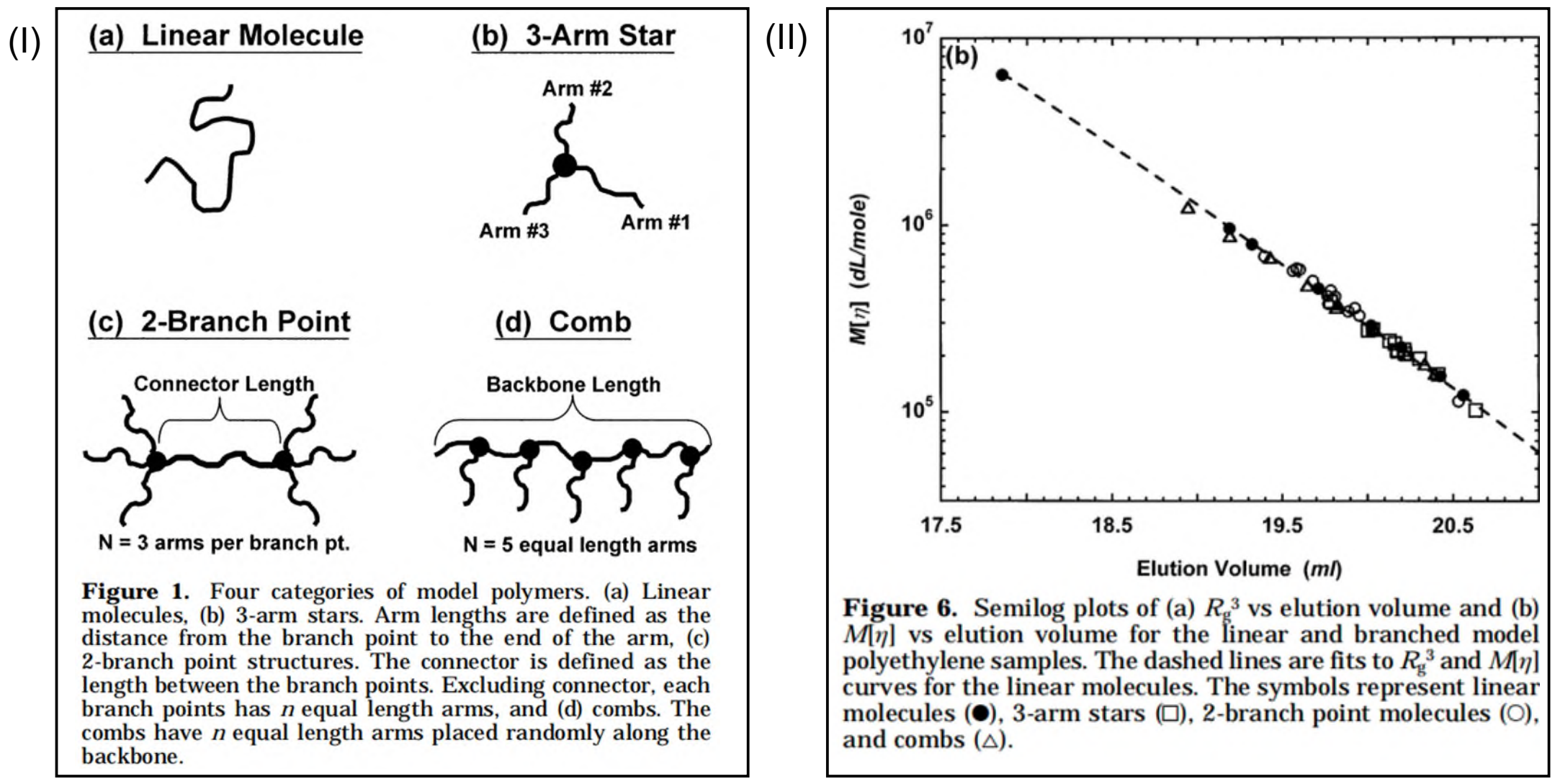

Figure R1. (I) Schematic representations of four categories of model polymers: (a) linear molecules, (b) 3-arm stars, (c) 2-branch point structures, and (d) combs. **(II)** Semilog plots of M[η] versus elution volume for linear and branched model polyethylene samples. The dashed line represents the fit to the M[η] curve for the linear molecules. The symbols represent linear molecules (solid circles), 3-arm stars (open squares), 2-branch point molecules (open circles), and combs (open triangles). All figures are reproduced with permission from Ref. [7] (Sun, T.; Chance, R. R.; Graessley, W. W.; Lohse, D. J. *Macromolecules* 2004, **37**, 4304-4312). Copyright 2004 American Chemical Society.

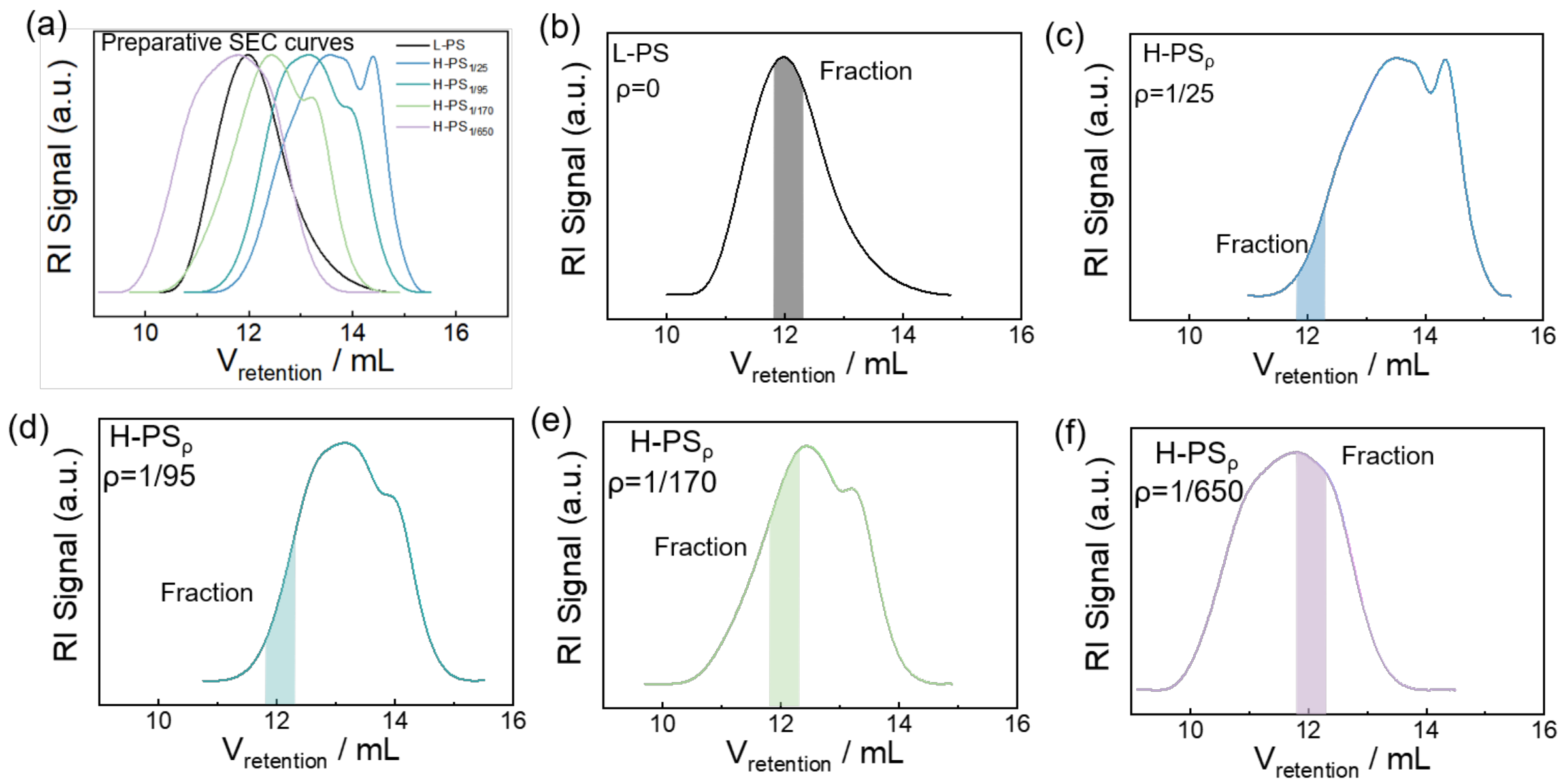

Figure R2. Preparative SEC fractionation of linear and hyperbranched polystyrene samples. (a) Overall preparative SEC elution profiles of the broadly distributed linear PS (L-PS) and four hyperbranched variants with varying branching densities (H-$PS_{1/25}$, H-$PS_{1/95}$, H-$PS_{1/170}$, and H-$PS_{1/650}$). (b-f) Isolation of specific fractions (indicated by the shaded regions) collected within an identical co-elution window (retention volume $V_{retention}$ = 11.8-12.3 mL, corresponding to the 11.8-12.3 min interception window at a flow rate of Q = 5 mL/min) for L-PS and all hyperbranched variants.

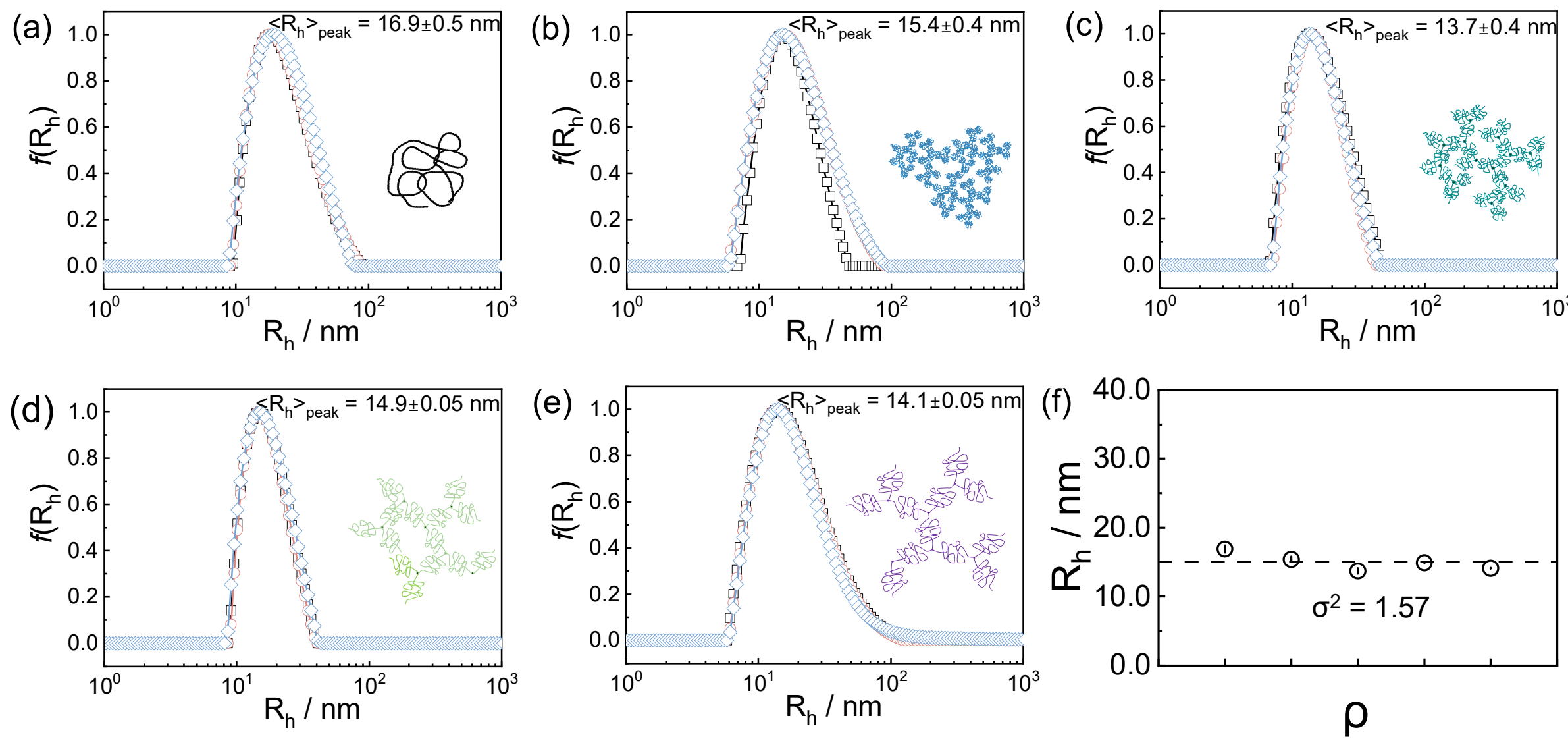

Figure R3. Dynamic light scattering (DLS) measurements of the co-eluting fractions captured within the specific interception window of 11.8-12.3 min. (a-e) Hydrodynamic radius (R_h) distributions for fractions obtained from the five topological systems: linear PS and four hyperbranched variants (H-$PS_{1/25}$, H-$PS_{1/95}$, H-$PS_{1/170}$, and H-$PS_{1/650}$. (f) Summary of the peak R_h values as a function of branching density (ρ).The dashed horizontal line denotes the overall average R_h value of 15 nm across all samples, confirming that these co-eluting fractions possess indistinguishable and closely matched R_h within experimental error (±2 nm) regardless of their topological architectures.

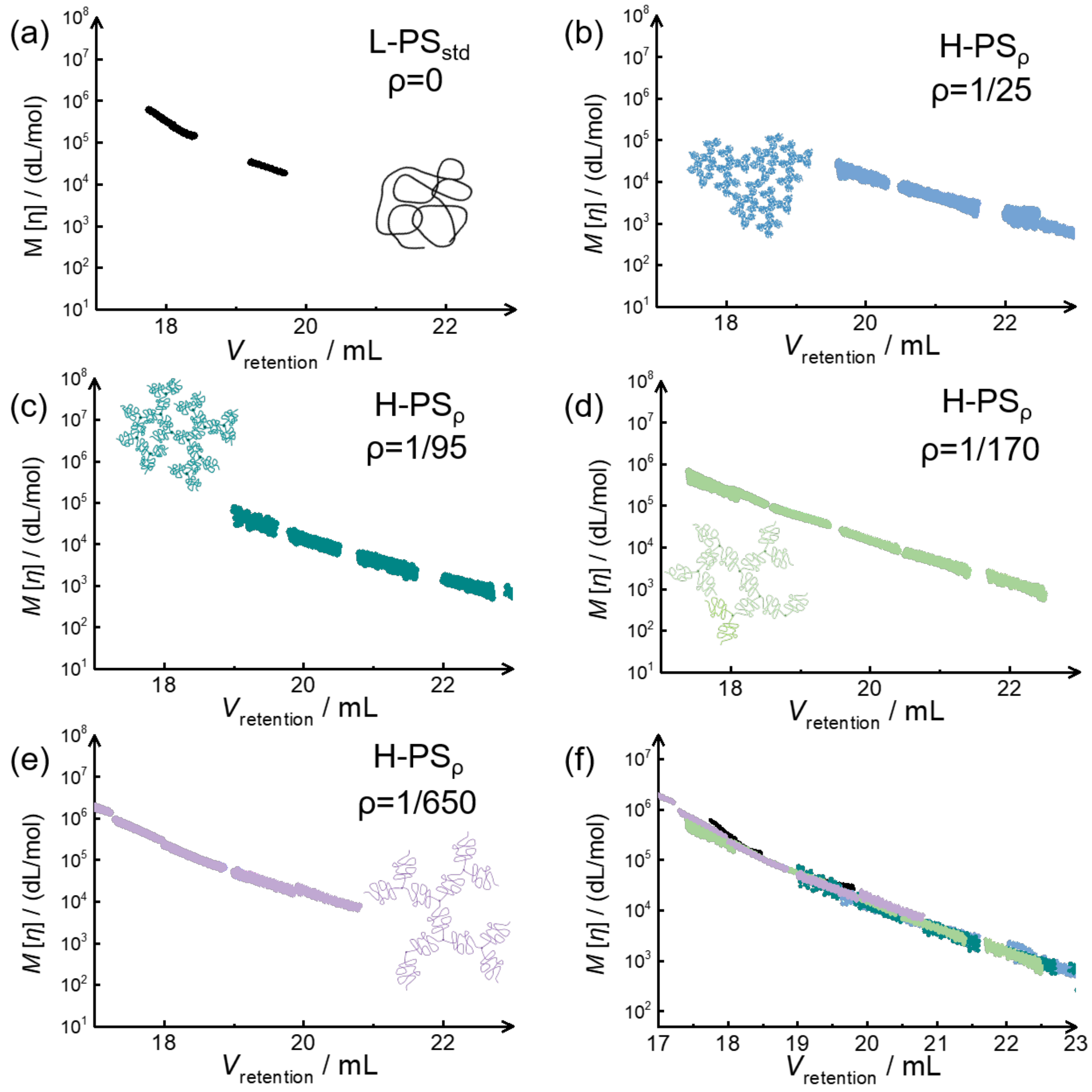

Figure R4. Quadruple-detector SEC (TD-SEC) validation of Benoit's Universal Calibration for fractionated linear and hyperbranched polystyrene samples. (a-e) Plots of the product of absolute molecular weight and intrinsic viscosity (M[η], which is strictly proportional to hydrodynamic volume) versus retention volume ($V_{retention}$) for linear PS (L-PS_{std}, ρ=0) and four hyperbranched fractions with varying branching densities (ρ=1/25, 1/95, 1/170, and 1/650). (f) An overlay of all data points from both the linear and hyperbranched fractions. The impeccable collapse of all data onto a single master curve demonstrates that the SEC retention volume in this system is a pure function of hydrodynamic size, completely devoid of topological bias.

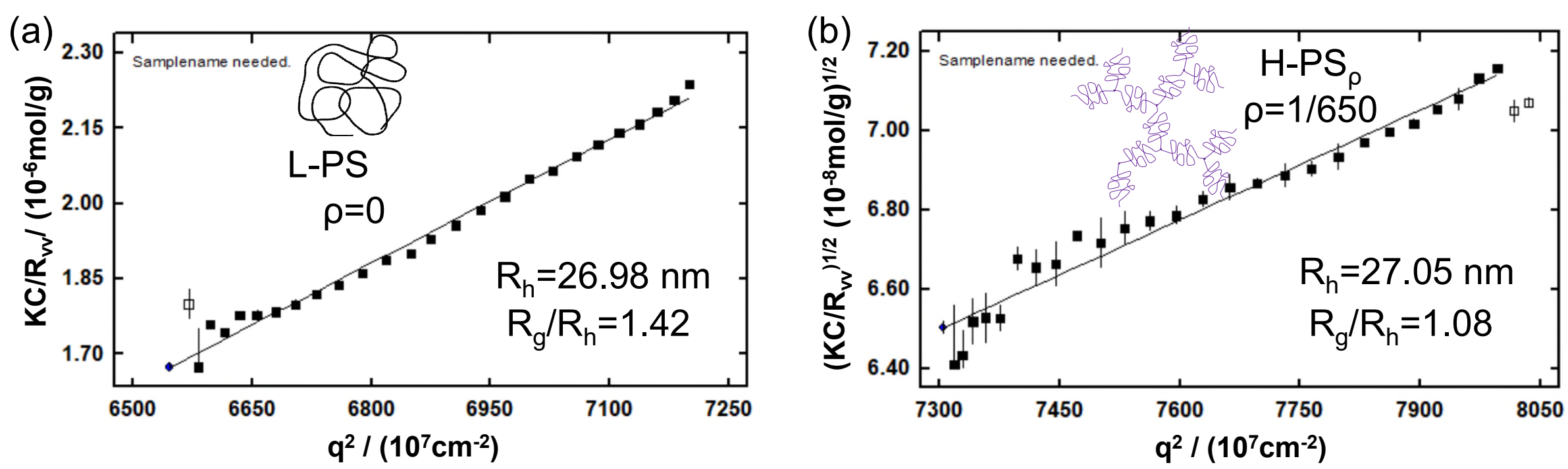

Figure R5. Single-concentration static/dynamic light scattering of co-eluting fractions. (a) Zimm plot for linear PS (L-PS, $R_g/R_h \approx 1.42$). (b) Berry plot for hyperbranched PS (H-PS$_{1/650}$, $R_g/R_h \approx 1.08$). Both fractions exhibit similar $R_h \approx 27$ nm. Note: Due to unknown absolute concentrations of SEC fractions, a default nominal concentration of C = 1 mg/mL was used for data processing, which does not affect the robustness of the topology-dependent shape factor (R_g/R_h).

2) **Question:** The experimental finding that the critical hydrodynamic interception size scales as $R_{h,c} \sim \rho^{-1/2}$ is a central highlight of the paper. While the experimental data fit this beautifully, the theoretical framework provides qualitative support but does not rigorously derive the exponent. The authors should either strengthen the derivation or present the scaling more cautiously as an empirical observation. Also, providing a physical basis for this exponent will greatly elevate the theoretical impact of the paper.
Reply: We thank you for this insightful comment. We fully agree that the observed scaling $R_{h,c} \sim \rho^{-1/2}$ is an experimental highlight, and that our current theoretical analysis provides only qualitative support, not a rigorous derivation of the exponent. We have addressed this as follows.

a) **Cautious presentation of the scaling**

In the revised manuscript, we have changed the wording from "reveals a power-law relationship" to "**exhibits an approximate scaling behavior** $R_{h,c} \sim \rho^{-1/2}$", and explicitly state that the exponent should be regarded as an **empirical observation** at this stage. The corresponding text in the Results and Conclusion sections has been adjusted accordingly.

b) **Physical basis for the exponent (qualitative)**

Although a rigorous derivation is not yet available, we can rationalize why $\rho^{-1/2}$ is physically meaningful. For idea hyperbranched chains, Flory theory (Rubinstein, M., Colby, R. H. Polymer Physics; Oxford University Press, **2003**) gives $R_g^2 \sim N_{total}N_{sub}^{1/2}$, i.e., $R_g \sim \rho^{-1/4}$. The critical interception size $R_{h,c}$ is mainly governed by the **core region** of the chain, not the outermost extensions. Branching makes the chain more spherical; R_h scales similarly to R_g but is slightly more sensitive to branching because it weights short distances more heavily. Experimentally, $R_{h,c} \sim \rho^{-1/2}$ implies $R_{h,c} \sim R_g^2$, suggesting that **the interception is related to the two-dimensional projected area of the chain** rather than its linear dimension. A qualitative blob analysis (Eq. 13 in the main text) shows that the free-energy barrier $\Delta E \sim N_{total}(R_h/D)^{1/(3\nu-1)}$. Setting $\Delta E \sim k_BT$ as the interception criterion and inserting the size scaling of hyperbranched chains leads to $R_{h,c}/D \sim \rho^{-1/2}$ under reasonable choices of exponents. We emphasize that this is not a strict derivation but provides a consistent physical picture.

c) **Fundamental theoretical difficulty**

As you points out, classical theories (Flory, blob models) are based on **average conformations** and ignore the **width of the conformational distribution**. However, our experiments and simulations show that the full width at half maximum (FWHM) of the R_g distribution is a key factor determining interception. Hyperbranched architectures exhibit an enormous diversity of configurations, and a rigorous treatment of many-body interactions and topological constraints on conformational fluctuations remains an unsolved challenge in the field. Therefore, deriving the exact exponent -1/2 from first principles is currently beyond reach. **This limitation is precisely why our experimental finding—together with the observed scaling—can stimulate future theoretical developments.**

d) **Revisions in the manuscript**

In the *Scaling Analysis of Critical Interception Size* section (**page 16 line 8-13**), we now state: "Strikingly, the experimental data exhibits an approximate empirical scaling behavior, $2R_{h,c}/D \sim \rho^-$

$^{1/2}$. From a physical perspective, since Flory theory predicts $R_g \sim \rho^{-1/4}$ for ideal hyperbranched chains, the observed scaling implies $R_{h,c} \sim R_g^2$. This intriguing relationship suggests that the nanopore interception is dominantly governed by the two-dimensional projected area of the dense topological core, rather than its simple linear dimension."

At the end of the theoretical analysis (**page 25 line 11-18**), we have added the following sentence: "It is worth noting that while the qualitative blob analysis conceptually supports the scaling of the free-energy barrier, a rigorous derivation of the exact -1/2 exponent from first principles remains challenging. … Therefore, the observed scaling is currently regarded as a robust empirical observation, highlighting a genuine theoretical gap regarding how topological constraints and many-body interactions restrict conformational fluctuations."

We believe these revisions appropriately address your concern by presenting the scaling more cautiously, providing a qualitative physical basis, and acknowledging the genuine theoretical difficulty.

3) **Question:** Simulations convincingly show reduced conformational fluctuations with increasing branching, supporting the proposed mechanism. However, a more direct quantitative link to $R_{h,c}$ or the observed scaling would strengthen the manuscript.

Reply: We agree with you that a direct quantitative link between simulations and the scaling exponent would strengthen the manuscript. **In the revised manuscript, we have therefore added a discussion based on the topology-dependent shape factor, defined as $\rho = R_g/R_h$ (1.08 for hyperbranched chains), which suggests that branching leads to a more compact architecture in which the mass distribution and hydrodynamic size become more closely coupled.** This parameter provides a semi-quantitative bridge between the simulated radius of gyration and the experimentally determined hydrodynamic radius. R_g reflects the spatial mass distribution of the polymer chain and is particularly sensitive to long-range chain extension, whereas R_h describes the hydrodynamic equivalent size of the molecule in solution. Therefore, R_g and R_h should not be treated as directly interchangeable quantities or converted by a simple linear relationship. Instead, their ratio reflects how the internal mass distribution of a polymer is translated into its hydrodynamic size.

We would also like to clarify the scope of the simulation. Real hyperbranched polymers contain a very large number of possible topological isomers, arising from different branching positions, branch lengths, terminal distributions and local crowding environments. Each topology may have its own conformational ensemble. Therefore, a rigorous quantitative prediction of the experimental $R_{h,c}$ value or the R_h-M scaling exponent would require extensive averaging over many topological isomers, molecular weights and conformational ensembles. The present simulation focuses on representative molecular architectures and is intended to reveal the molecular origin of the topology-dependent size behavior rather than to directly derive the experimental scaling exponent from a single fixed topology. **A major challenge in the field is that the many-body effects governing hydrodynamic interactions are fundamentally different from those governing static chain dimensions**. Theory that simultaneously accounts for both remains lacking. Therefore, our simulations are used to establish the mechanism (reduced fluctuation amplitude with increasing branching), not to derive the exponent directly.

Nevertheless, the simulations do provide a direct link to $R_{h,c}$ via the distribution width: **for a specific configuration of hyperbranched chains, the FWHM of R_g narrows drastically from linear (0.82) to hyperbranched (0.17).** This suppressed fluctuation raises the free-energy barrier for pore entry, shifting interception to a smaller relative size. We have clarified this mechanistic link in the revised manuscript and explicitly state that the exponent $\rho^{-1/2}$ remains empirically determined, pending future theoretical

advances that can treat configurational diversity and hydrodynamic many-body effects on an equal footing.

Revisions in the manuscript

We have revised the description and analysis of Figure 7 (**page 21 line 6** to **page 22 line 3**) of the manuscript as follows："To elucidate the molecular origins of topology-dependent interception, we examined the simulated radius of gyration (R_g) distributions (**Figure 7c**). … This tighter coupling of spatial mass distribution and hydrodynamic size, synergizing with the drastically narrowed FWHM, directly raises the free-energy barrier for pore entry, inherently shifting the critical interception ($R_{h,c}$) to a smaller relative size."

4) **Question:** In the SI, the authors mention the 700-mer polystyrene chains subjected to 100 ns of cyclic annealing. While cyclic annealing is a valid strategy to overcome local minima, 100 ns may not be enough for a polymer of this size and topological complexity to achieve global equilibration. The authors should provide supplementary data such as R_g or end-to-end distances demonstrating that the chains are indeed fully equilibrated.

Reply: We thank you for pointing out the importance of verifying the equilibration of large and topologically complex polymer chains. **In response to your suggestion, we performed an additional 20 ns constant-temperature molecular dynamics simulation starting from the final configuration obtained after the first 100 ns cyclic annealing simulation.**

We would like to clarify the relationship between the original and newly added simulations. In the original manuscript, all statistical results were obtained from the first 100 ns simulation trajectories. The first 100 ns were cyclic annealing simulations, which were used to help the chains overcome local conformational minima and accelerate conformational rearrangement. Based on your comments, we further extended the simulations by 20 ns under constant-temperature conditions. Therefore, the newly added 100–120 ns trajectories were used as a stability check for the late-stage configurations obtained after the first 100 ns cyclic annealing simulations, rather than as a replacement for the statistical basis of the original manuscript.

To evaluate whether the first 100 ns simulations had reached a stable regime with respect to the key structural observable used in this work, we compared the average R_g values during the last 20 ns of the first 100 ns trajectories, namely 80–100 ns, with those obtained from the additional 100–120 ns constant-temperature simulations. The results show that the R_g values remain close to the late-stage plateau values. Specifically, the average R_g values for the linear, comb-like, dendritic, hyperbranched-1 and hyperbranched-2 systems changed from 8.238, 5.025, 4.117, 3.821 and 4.627 nm during 80–100 ns to 8.485, 5.137, 4.120, 3.789 and 4.678 nm during 100–120 ns, respectively. The corresponding relative changes are +3.0%, +2.2%, +0.1%, −0.8% and +1.1%, respectively. These small changes indicate that the additional 20 ns constant-temperature simulations are continuous with the late-stage plateau of the first 100 ns cyclic annealing simulations and that no new systematic drift in R_g is observed.

We further performed a 5 ns block-average analysis for the additional 100–120 ns trajectories. For the linear chain, the average R_g values in the 100–105, 105–110, 110–115 and 115–120 ns intervals are 8.533, 8.408, 8.653 and 8.359 nm, respectively. For the comb-like system, the corresponding values are 5.092, 5.121, 5.210 and 5.126 nm. For the dendritic system, they are 3.879, 4.226, 4.150 and 4.216 nm. For hyperbranched-1, they are 3.783, 3.792, 3.769 and 3.810 nm. For hyperbranched-2, they are 4.565, 4.563, 4.766 and 4.804 nm. These block averages show that R_g fluctuates around the late-stage plateau values during the additional constant-temperature simulations, without a persistent monotonic drift.

In the revised Supplementary Information, we have added the 0–120 ns R_g time evolution

curves ([Figure R6](#)), and attached the original data file to the cover letter. The first 100 ns correspond to cyclic annealing simulations, and the last 20 ns correspond to the additional constant-temperature simulations. We have also included the comparison between the 80–100 ns and 100–120 ns average R_g values, as well as the 5 ns block-average results for the additional simulation stage.

We have also revised the statement regarding equilibration to avoid overstatement. For such large and topologically complex polymer systems, it is difficult to prove complete global equilibration of the entire conformational space based on a single trajectory. **Therefore, instead of claiming absolute "full equilibration", we now state that the key structural observable considered in this work, namely R_g, reaches a stable late-stage plateau within the present simulation protocol**. The additional 20 ns constant-temperature simulations further confirm that the first 100 ns results are stable with respect to the relative comparison among different topologies. Thus, the main simulation conclusions of the manuscript remain robust.

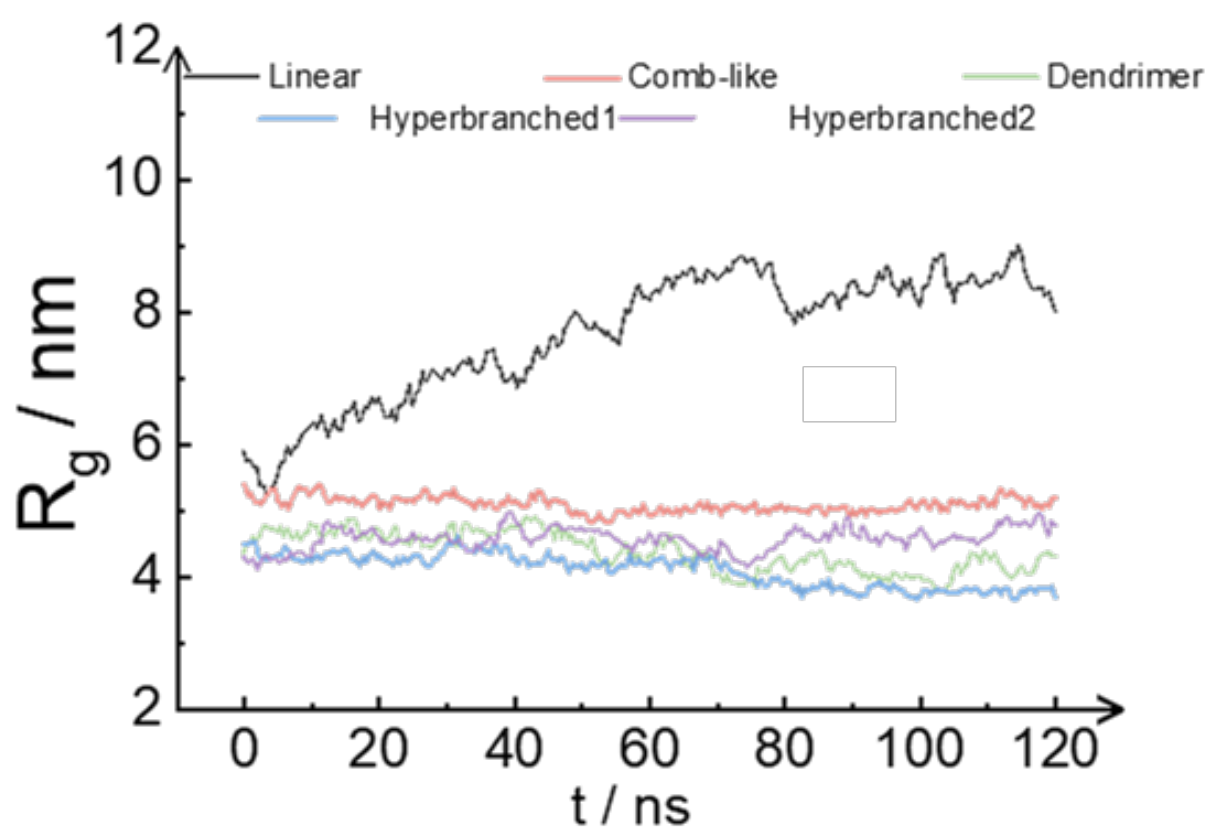

Figure R6. Time evolution of the radius of gyration (R_g) over 120 ns all-atom molecular dynamics simulations for the (a) linear, (b) comb-like, (c) dendritic, (d) hyperbranched-1 (H-PS$_{1/14}$), and (e) hyperbranched-2 (H-PS$_{1/7}$) polymer systems, each comprising 700 monomers.

Revisions in the manuscript

We have revised the *Conformational Distribution and Fluctuation by Computational Simulations* section (**page 19 line 21** to **page 20 line 4**) of the manuscript as follows: "Given the immense topological complexity of these large macromolecules, achieving absolute global equilibration is fundamentally challenging. To rigorously overcome local conformational minima, the systems were initially subjected to a 100 ns cyclic annealing protocol (**Figure S15**). Subsequent extended constant-temperature simulations (up to 120 ns) confirmed that the key structural observable, R_g, had reached a stable late-stage plateau without persistent monotonic drift, thereby ensuring the statistical robustness of our comparative topological analysis."

5) **Question:** Uncertainty in P_t (±3%) is reported but not propagated to $R_{h,c}$. What uncertainty was considered while defining blue region? Also, in Figures depicting the translocation probability (P_t) vs. critical retention volume or hydrodynamic radius, please ensure that the method used to calculate the error bars is explicitly stated in the relevant figure captions.

Reply: We fully agree with you that a clear explanation of error propagation is essential for quantitative reliability. We have addressed this carefully in our revised manuscript as follows:

a) Propagation of Uncertainty from P_t to $R_{h,c}$

The estimated ± 3% uncertainty in the translocation probability (P_t) primarily originates from minor

baseline fluctuations during SEC measurements, variations in the signal-to-noise ratio at the edges of the molecular weight distribution, and slight deviations during the peak normalization process. To propagate this uncertainty to the critical interception size ($R_{h,c}$), we mapped the ± 3% vertical deviation in P_t at the critical interception threshold onto the horizontal axis (retention volume) to obtain the uncertainty range $\Delta V_{r,c}$. Using our established SEC universal calibration scaling laws (as shown in **Figure S4** and **S5**), this $\Delta V_{r,c}$ is mathematically converted into the specific uncertainty of the hydrodynamic radius, $\Delta R_{h,c}$. We found that a ± 3% variance in P_t typically translates to an approximate error of: ± 2 nm in $R_{h,c}$, depending on the slope of the calibration curve at that specific volume. We have now incorporated these precise $\Delta R_{h,c}$ error bars into the revised figures.

b) Uncertainty Considered in Defining the Blue Region

The shaded blue region in Figure 5b, which highlights the scaling relationship $2R_{h,c}/D \sim \rho^{-1/2}$, visually represents a compounded tolerance band. When defining this region, we considered two major sources of uncertainty that affect the y-axis ($2R_{h,c}/D$):

- Numerator Uncertainty ($\Delta R_{h,c}$): The propagated error from SEC P_t calculation as described above.
- Denominator Uncertainty (ΔD): The inherent structural dispersity of the AAO nanopore diameters. Although the pores are highly uniform, statistical analysis from SEM imaging and Poiseuille fitting (**Table S3**) reveals a slight size variation (**e.g., standard deviations of ~ 1-2 nm**).

Therefore, the upper and lower bounds of the blue region reflect the maximum and minimum possible values of $2R_{h,c}/D$ derived from standard error propagation principles:

$$\frac{\Delta(2R_{h,c}/D)}{2R_{h,c}/D} = \sqrt{\left(\frac{\Delta R_{h,c}}{R_{h,c}}\right)^2 + \left(\frac{\Delta D}{D}\right)^2}$$

This provides a rigorous physical basis for demonstrating that all experimental data points fall within the theoretically predicted scaling envelope.

As suggested, we have updated the relevant figure captions (e.g., **Figures 4h, 5a**, and **S13-S14**) and the Methods section (**Supplementary Information page 9 line 4 to 10**) to explicitly describe the origin of the error bars. Accordingly, a short discussion has been added: "Error bars for the translocation probability (P_t) represent a ± 3% instrument/baseline uncertainty derived from repeated SEC normalization. Horizontal error bars for the critical hydrodynamic radius ($R_{h,c}$) represent the propagated uncertainty, calculated by mapping the P_t error onto the retention volume axis and converting it via the SEC calibration equation. The shaded blue region encompasses the compounded standard deviation considering both the measurement uncertainty of $R_{h,c}$ and the structural size distribution (ΔD) of the AAO nanopores."

6) **Question:** In Figure 7, It is not clear where the FWHM comes from? Is it from Rg distribution? It should be clearly mentioned in captions and the distributions should be provided. Df is also not defined and what does it signify?

Reply: We sincerely thank you for pointing out these omissions. We have explicitly defined FWHM and D_f to ensure the clarity and rigor of the computational and theoretical discussions in the revised manuscript:

a) Clarification on FWHM (Full Width at Half Maximum)

The FWHM values are directly extracted from the radius of gyration (R_g) distribution curves obtained from our all-atom molecular dynamics simulations. We would like to respectfully clarify that these R_g distribution curves are already plotted and provided in **Figure 7c** of the manuscript. Physically,

the FWHM of the R_g distribution serves as a quantitative metric for the amplitude of conformational fluctuations. A broader distribution with a larger FWHM (e.g., linear chains, FWHM = 0.820) indicates high conformational flexibility and a wide range of accessible states. In contrast, a narrower distribution with a smaller FWHM (e.g., hyperbranched chains, FWHM ~ 0.17-0.18) signifies a more rigid, compact architecture where conformational fluctuations are significantly restricted. This restricted fluctuation directly contributes to the higher free-energy barrier for pore entry (the conformational entropy bottleneck) observed in our experiments.

b) Definition and Significance of D_f

D_f stands for the Fractal Dimension, a fundamental parameter in polymer physics used to characterize the spatial compactness and mass distribution of polymer architectures. It relates the polymer's mass (M) to its size (R) via the scaling law $M \sim R^{D_f}$. In **Figure 7b**, D_f is used to illustrate the structural evolution from flexible coils to rigid spheres:

- For flexible linear polymers in a good solvent, the structure is relatively loose, corresponding to $D_f \sim 1.7$ (since $R_g \sim M^{0.6}$).
- For a hard sphere, the mass fills the 3D space uniformly, yielding $D_f = 3.0$.
- For hyperbranched polymers, the value falls in between (e.g., $D_f \sim 2.2$).

The significance of D_f in our context is to visually and physically demonstrate that hyperbranched chains are much denser and more sphere-like than linear chains. This architectural compactness explains why their interception behavior is largely governed by topology rather than temperature-induced swelling, distinguishing them from linear coils.

c) Revisions in the Manuscript

We have revised the description and analysis of Figure 7 (**page 21 line 6-19**) of the manuscript as follows: "The full width at half maximum (FWHM) of R_g serves as a precise quantitative metric for conformational fluctuations. … This structural densification is quantitatively evaluated by the fractal dimension (D_f, $M \sim R^{D_f}$). While linear chains adopt swollen random coils ($D_f = 1.7$), hyperbranched polymers exhibit more compact, space-filling architectures ($D_f = 2.2$), approaching the theoretical limit of a non-fluctuating solid hard sphere ($D_f = 3.0$)."

7) **Question:** Methods section should be revised to add more technical details to ensure reproducibility.

Reply: We sincerely thank you for this constructive suggestion. We completely agree that the intricate nature of the weak-compression interception experiment requires comprehensive technical details to ensure full reproducibility. In response to your valuable comment, we have substantially expanded the Methods section to provide a step-by-step, standardized protocol for the interception tests. Specifically, we have detailed three crucial experimental stages that guarantee the physical isolation of polymer chains and the total dominance of Brownian diffusion over flow-induced stretching:

Preparation of Ultra-Dilute Solutions: We clarified that the polymer stock concentrations were strictly capped below 1.00 g/L (less than 1/10 of the critical overlap concentration, C^*). This mathematically eliminates multi-chain synergistic translocation and inter-chain entanglement.

In-situ Hydrodynamic Calibration: We added the specific parameters for membrane assembly and calibration, including the strict thermostatic control at 25 ± 1 °C and the pre-flushing with pure solvent at 6 mL/h to determine the exact effective hydrodynamic pore size in-situ via the Poiseuille equation.

Precise Flow Control and Immediate Analysis: We detailed the extreme macroscopic flow rate limit of 0.2 mL/h , which ensures that the localized shear energy is negligible (~10^{-6} k_BT), leaving translocation entirely governed by conformational entropy. We also explicitly described the dead-volume flushing

protocol (discarding the initial 250 μL) and our strict "immediate SEC testing" strategy for the effective permeate to prevent system errors.

Revisions in the Supplementary Information

We believe these additions thoroughly address your concerns regarding experimental reproducibility. The technical details have been incorporated into the **revised Supplementary Information (page S6 line 19** to **page S8 line 10)** as follows:

"**Standardized Protocol for Polymer Interception under the Weak-Compression Limit**

Preparation of Ultra-Dilute Polymer Solutions. To isolate the intrinsic interception behavior and fundamentally eliminate artifacts caused by multi-chain synergistic translocation, the polymer stock solutions were prepared at extremely dilute concentrations. The concentration was strictly capped … To thoroughly flush any residual solvent from the system's dead volume, the initial 250 μL of the filtrate was discarded. Once the system reached a fully stable operational state, exactly 250 μL of the effective permeate was collected. To maximize data fidelity and prevent systemic errors originating from time-dependent column drift, we strictly adhered to an "immediate sampling and testing" protocol, instantly subjecting the collected permeate to SEC analysis."

To the comments of reviewers 2 and 4 (co-reviewed):

Reviewer 2: I co-reviewed this manuscript with one of the reviewers who provided the listed reports. This is part of the Nature Communications initiative to facilitate training in peer review and to provide appropriate recognition for Early Career Researchers who co-review manuscripts.

Reviewer 4: In this work, Wang et al used hyperbranched polystyrene, monodisperse AAO nanopores, all atom MD simulations, and theoretical modeling to focus how branching topology controls the critical interception size of polymers in nanopores under the weak-compression limit. They identified a unique scaling relation Rh, c ~ ρ-1/2 for hyperbranched polymers, distinct from linear chains. The present work is novel so that it is suitable to be published on NC. Nevertheless, I have a few comments which I list below in no particular order:

Reply: We sincerely thank you for the positive evaluations of our work. We have carefully considered your comments and made the necessary revisions to strengthen our manuscript further. We believe that these enhancements will greatly improve the clarity of our manuscript and its overall impact.

1) **Question:** 'Figure 1'simplification: Reduce subplots by moving repetitive curves to the SI; keep only representative data for better readability.

Reply: We thank you for this constructive suggestion. We fully agree that simplifying Figure 1 significantly improves the overall readability of the manuscript. Accordingly, we have streamlined Figure 1 by moving the apparent radius of gyration (R_g) profiles to the Supplementary Information (**now Figure S6**), as shown below (**Figure R1**).

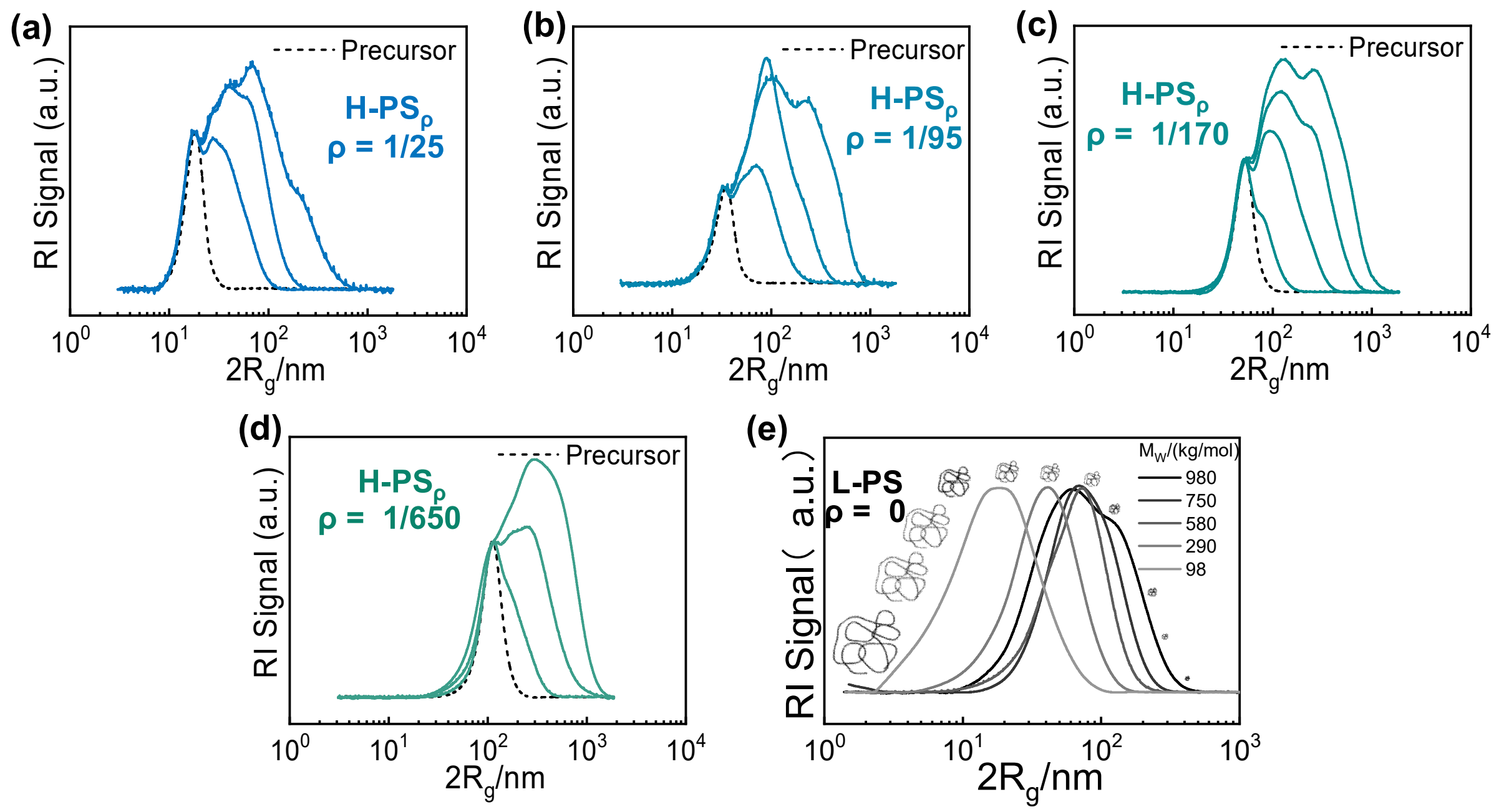

Figure R1. Apparent radius of gyration ($R_{g,app}$) profiles for polystyrene samples with varying branching densities: (a) ρ = 1/25, (b) ρ = 1/95, (c) ρ = 1/170, (d) ρ = 1/650 and (d) ρ = 0.

2) **Question:** SEC validation: It should be noted that SEC only provides apparent molecular weight for nonlinear polymers, and the hydrodynamic radius is converted using universal scaling laws established from linear standards. For hyperbranched polymers with significantly different architectures, especially high-branching-density systems, the quantitative accuracy of this conversion method requires further support. I recommend to provide dynamic light scattering measurements of key samples to verify the

reliability of Rh distributions converted from SEC using hydrodynamic radius, thereby improving the rigor of key data.

Reply: We thank you for raising this fundamental and important question regarding the validity of using linear polystyrene standards to calibrate the hydrodynamic radius of hyperbranched polymers. We fully agree that this is a critical issue for the interpretation of our data. Below we provide a comprehensive response, grounded in **both classical literature and our own rigorous experimental validations**.

a) **Classical SEC Theory Establishes Hydrodynamic Volume as the Universal Separation Principle**

Since the seminal work of Grubisic, Rempp, and Benoit (1967), **it has been firmly established that size exclusion chromatography separates polymers based on their hydrodynamic volume, $V_h \sim [\eta]M$**, regardless of polymer species or chain architecture (linear, star, comb, etc.) [Grubisic, Z.; Rempp, P.; Benoit, H. J. *Polym. Sci. B Polym. Lett.* **1967**, *5*, 753.]. This "universal calibration" concept is the cornerstone of modern SEC.

More importantly, Teraoka (Teraoka, I. *Macromolecules* **2004**, *37*, 6632.) provided a rigorous theoretical foundation by calculating the partition coefficients of polymers with various topologies (asymmetric stars, two-branch-point, combs) in slit-like pores. He demonstrated unequivocally that **the hydrodynamic radius R_h, not the radius of gyration R_g, is the correct molecular dimension that dictates the partitioning behavior**. Specifically, when the partition coefficient K was plotted against R_h/D (pore size ratio), all data for different architectures collapsed onto a single master curve. In contrast, plotting against R_g/D resulted in significant deviations. Since R_h is directly proportional to the cube root of the hydrodynamic volume ($R_h \sim ([\eta]M)^{1/3}$), Teraoka's work provides the theoretical basis for why SEC retention volume universally correlates with R_h.

Thus, using SEC retention volume to determine **R_h is not an empirical approximation but a physically grounded method, valid for linear and branched polymers alike.**

b) **Reported Experimental Evidence for Branched Polymers Follows the Same Principle**

Sun et al. (Sun, T.; et al. *Macromolecules* **2004**, *37*, 4304.) conducted an extensive SEC study on linear and branched polyethylenes (3-arm stars, H-shaped, and combs). Their key finding is highly relevant to our work: **For branched polymers, the elution volume satisfactorily correlates with $[\eta]M$ (and thus R_h), as shown below (Figure R2)**. In contrast, branched polymers deviated strongly from the linear R_g curve but remained on the $[\eta]M$ curve. **This directly demonstrates that for non-linear architectures, hydrodynamic volume (or R_h) is the sole determinant of SEC elution.**

Furthermore, Wang et al. (Wang, Y.; et al. *Macromolecules* **2011**, *44*, 403.) extended the theoretical analysis of "mean span dimension" and showed that for branched polymers, the reduction in size relative to linear chains is much milder for R_h and span dimension than for R_g[4]. This is because branching primarily compresses the distal segments (affecting R_g) but has a less dramatic effect on the core density (affecting R_h). **Consequently, R_h remains a robust and topology-independent metric for SEC calibration, even for highly branched structures like our hyperbranched PS.**

c) **Our Experimental Validation Confirms No Topology-Induced Bias**

Despite the strong literature support, we recognized that hyperbranched polymers with extremely high branching density (e.g., H-$PS_{1/25}$) might push the limits of conventional universal calibration. **Therefore, we performed three independent experimental validations in the revised manuscript (see Figures R3-R5 and associated discussion).**

- **Co-elution + Offline DLS (Data for Publication)**: We performed high-precision preparative SEC (Waters Ultrastyragel Linear THF, 19×300 mm, Q = 5 mL/min) to systematically fractionate both the broadly distributed linear PS (L-PS) and all four hyperbranched variants (H-$PS_{1/25}$, H-$PS_{1/95}$, H-$PS_{1/170}$, and H-$PS_{1/650}$), as shown in **Figure R2**. Samples were meticulously collected in 0.5-minute slice intervals. By deliberately shifting the collection windows based on their respective broad elution profiles, we successfully isolated series of narrow-distribution fractions. **Crucially, this protocol allowed us to obtain highly valuable "co-eluting matrices"—fractions of vastly different architectural densities that eluted at the exact same retention time window**. In the specific interception window of 11.8-12.3 min, we successfully captured fractions from all five topological systems (L-PS F3, H-$PS_{1/25}$ F1, H-$PS_{1/95}$ F2, H-$PS_{1/170}$ F3, and H-$PS_{1/650}$ F6). **DLS measurements confirmed that these fractions have indistinguishable R_h within experimental error (±2 nm), as shown in Figure R3.**
- **Quadruple-detector SEC (TD-SEC) (Data for Review Only)**: To provide ultimate theoretical closure, these fractionated samples were further subjected to quadruple-detector SEC (TD-SEC). By plotting the product of intrinsic viscosity and absolute molecular weight ($M[\eta]$, strictly proportional to hydrodynamic volume) against the retention volume ($V_{retention}$), **all data points from both linear and hyperbranched variants impeccably collapsed onto a single master curve (Benoit's Universal Calibration), as shown in Figure R4**. Together, these multi-dimensional fractionations and absolute scattering verifications provide direct, unambiguous physical evidence that the SEC retention volume in our system is a pure function of hydrodynamic size, devoid of topological bias. Consequently, calibrating the R_h of our hyperbranched systems using linear standards is both physically sound and quantitatively highly reliable. Considering the importance of our experimental result, we respectfully request permission to publish the TD-SEC result elsewhere.
- **Shape Factor Analysis (Data for Review Only)**: Single-concentration static/dynamic light scattering on co-eluted fractions gave $R_g/R_h \approx 1.42$ for linear PS and ≈ 1.08 for hyperbranched PS (H-PS~1/650), confirming the expected coil-to-globule compaction. Absolute concentration was not determined, because accurate concentration determination is difficult for SEC-fractionated samples; the shape factor itself is robust in the dilute regime. **The data (Figure R5) is provided for reviewers only and will not appear in the published manuscript.**

d) Revisions in the Manuscript

We have revised the description and analysis of **page 6 line 18** to **page 7 line 7** of the manuscript as follows: "It is well-established by rigorous theoretical calculations that SEC separation is universally governed by hydrodynamic volume (or R_h), rather than R_g, irrespective of chain topology. … To experimentally verify that topological differences do not introduce systematic bias in our R_h determination, preparative SEC fractionation and offline DLS were performed on co-eluting matrices (**Figures S8** and **S9**), confirming precise physical size equivalence."

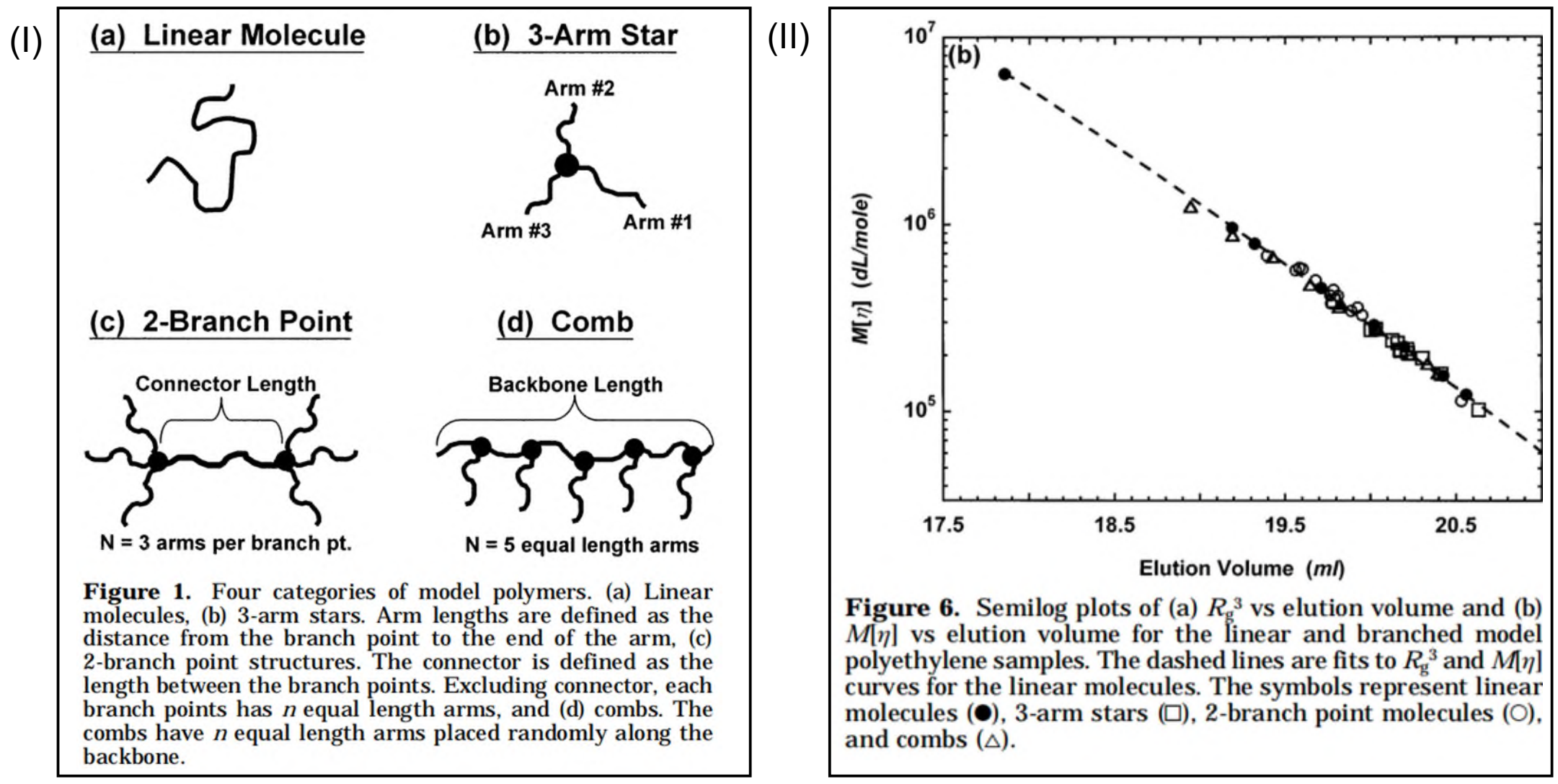

Figure R2. **(I)** Schematic representations of four categories of model polymers: (a) linear molecules, (b) 3-arm stars, (c) 2-branch point structures, and (d) combs. **(II)** Semilog plots of M[η] versus elution volume for linear and branched model polyethylene samples. The dashed line represents the fit to the M[η] curve for the linear molecules. The symbols represent linear molecules (solid circles), 3-arm stars (open squares), 2-branch point molecules (open circles), and combs (open triangles). All figures are reproduced with permission from Ref. [7] (Sun, T.; Chance, R. R.; Graessley, W. W.; Lohse, D. J. *Macromolecules* 2004, **37**, 4304-4312). Copyright 2004 American Chemical Society.

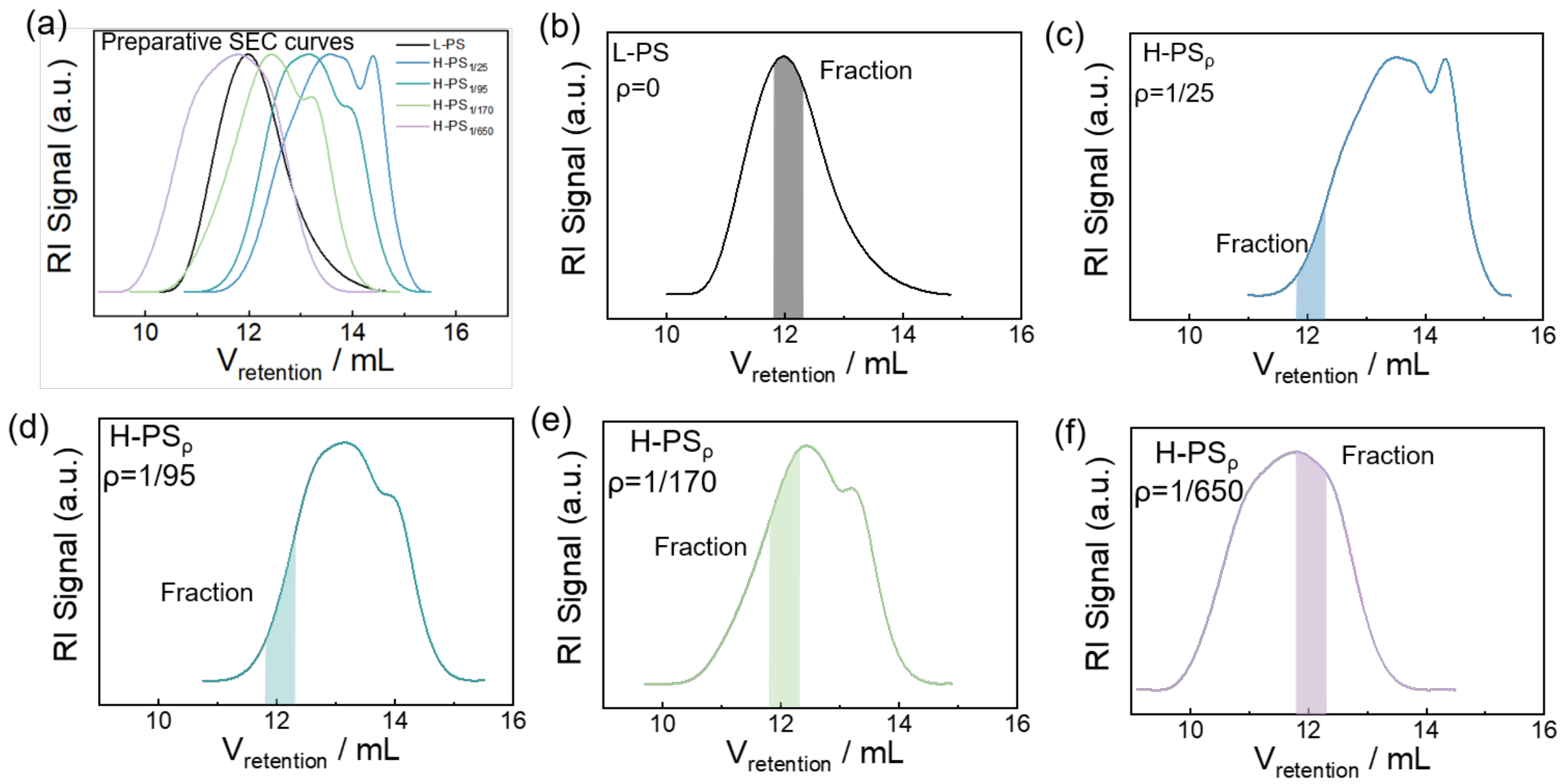

Figure R3. **Preparative SEC fractionation of linear and hyperbranched polystyrene samples.** (a) Overall preparative SEC elution profiles of the broadly distributed linear PS (L-PS) and four hyperbranched variants with varying branching densities ($H\text{-}PS_{1/25}$, $H\text{-}PS_{1/95}$, $H\text{-}PS_{1/170}$, and $H\text{-}PS_{1/650}$). (b-f) Isolation of specific fractions (indicated by the shaded regions) collected within an identical co-elution window (retention volume $V_{retention}$ = 11.8-12.3 mL, corresponding to the 11.8-12.3 min interception window at a flow rate of Q = 5 mL/min) for L-PS and all hyperbranched variants.

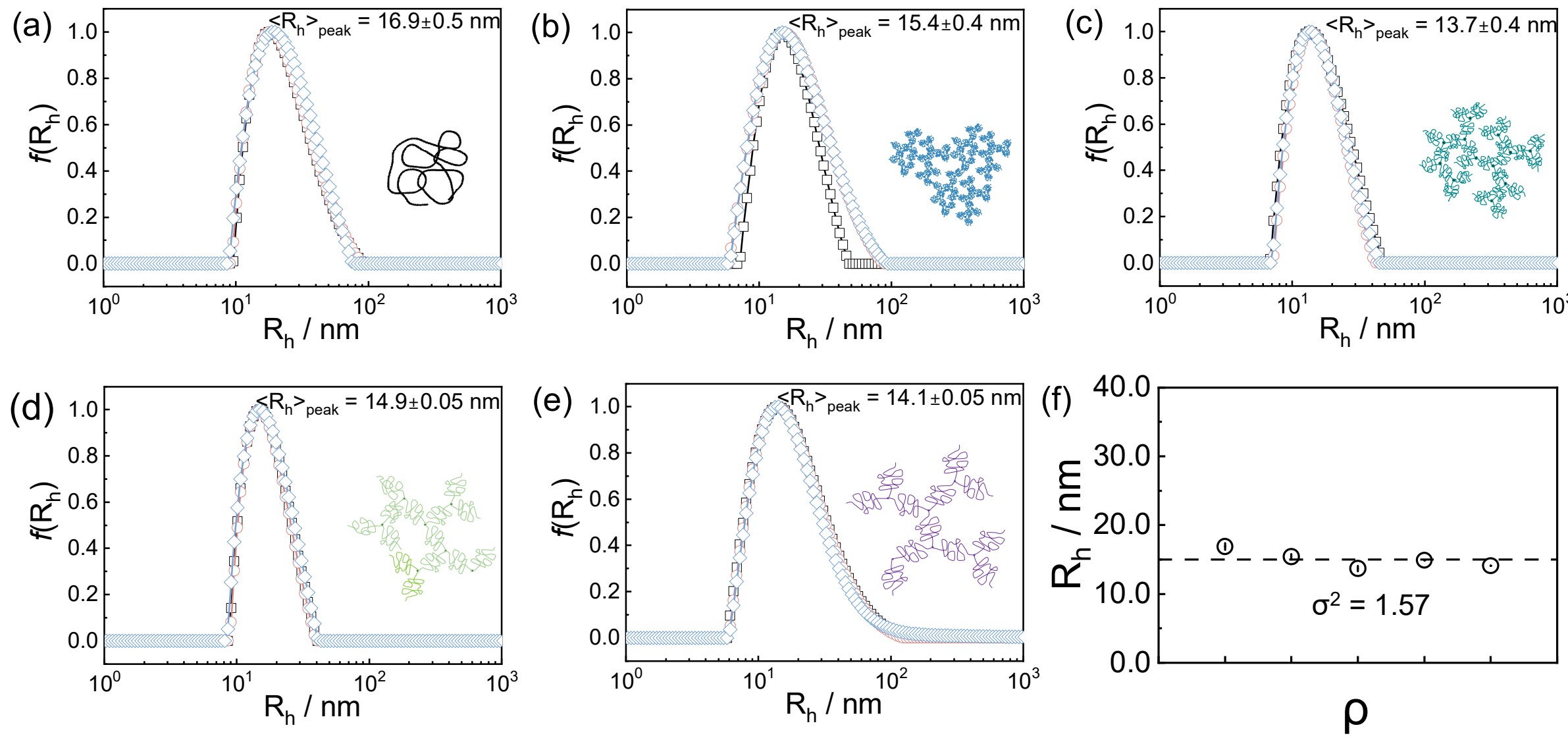

Figure R4. Dynamic light scattering (DLS) measurements of the co-eluting fractions captured within the specific interception window of 11.8-12.3 min. (a-e) Hydrodynamic radius (R_h) distributions for fractions obtained from the five topological systems: linear PS and four hyperbranched variants (H-$PS_{1/25}$, H-$PS_{1/95}$, H-$PS_{1/170}$, and H-$PS_{1/650}$. (f) Summary of the peak R_h values as a function of branching density (ρ).The dashed horizontal line denotes the overall average R_h value of 15 nm across all samples, confirming that these co-eluting fractions possess indistinguishable and closely matched R_h within experimental error (±2 nm) regardless of their topological architectures.

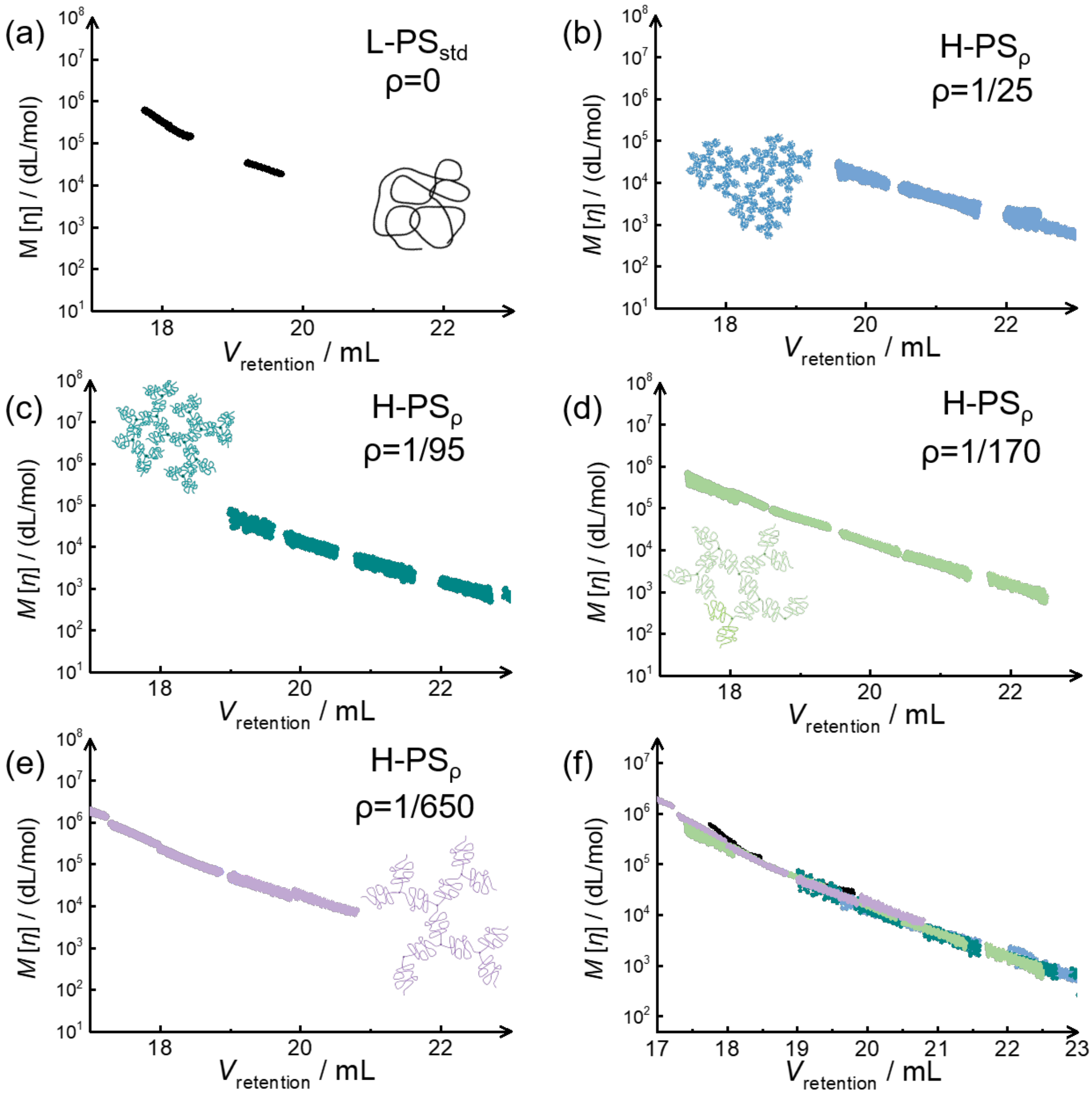

Figure R5. Quadruple-detector SEC (TD-SEC) validation of Benoit's Universal Calibration for fractionated linear and hyperbranched polystyrene samples. (a-e) Plots of the product of absolute molecular weight and intrinsic viscosity (M[η], which is strictly proportional to hydrodynamic volume) versus retention volume ($V_{retention}$) for linear PS (L-PS$_{std}$, ρ=0) and four hyperbranched fractions with varying branching densities (ρ=1/25, 1/95, 1/170, and 1/650). (f) An overlay of all data points from both the linear and hyperbranched fractions. The impeccable collapse of all data onto a single master curve demonstrates that the SEC retention volume in this system is a pure function of hydrodynamic size, completely devoid of topological bias.

3) **Question:** I recommend to also use static light scattering to measure R_g and dynamic light scattering to measure R_h, so that the shape factor R_g/R_h can be obtained. And the shape factor R_g/R_h is a perfect parameter to quantify the degree of branching.

Reply: We thank you for the constructive comment. Single-concentration static/dynamic light scattering on co-eluted fractions gave $R_g/R_h \approx 1.42$ for linear PS and ≈ 1.08 for hyperbranched PS (H-PS~1/650), confirming the expected coil-to-globule compaction. Absolute concentration was not determined, because accurate concentration determination is difficult for SEC-fractionated samples; the shape factor itself is robust in the dilute regime. **The data (Figure R6, Data for Review Only) is provided for reviewers only and will not appear in the published manuscript.**

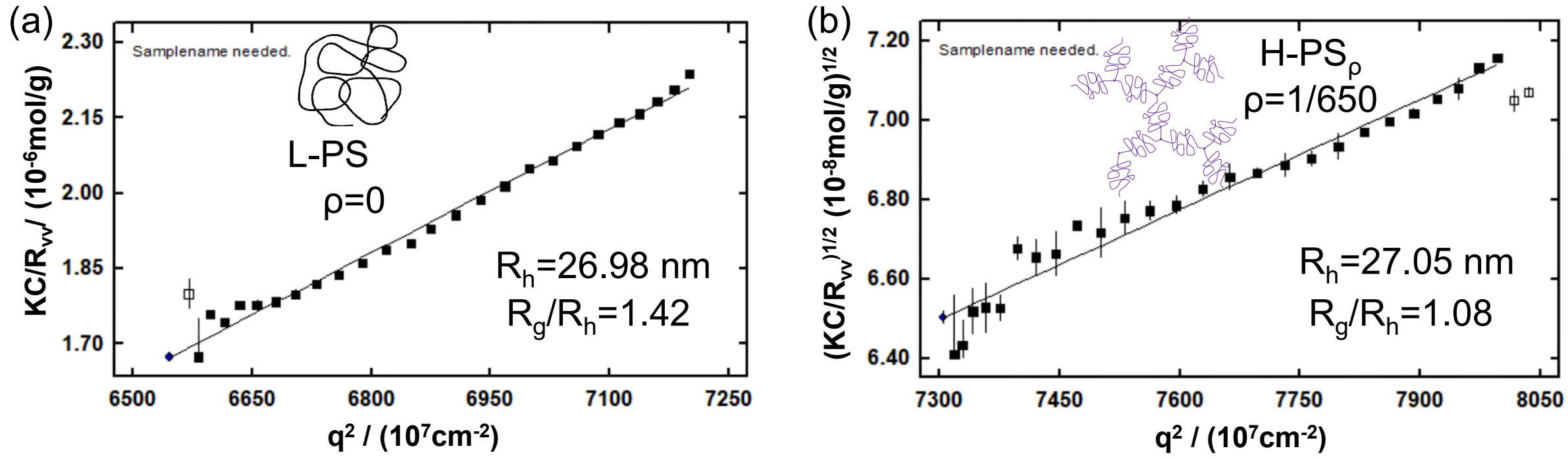

Figure R6. Single-concentration static/dynamic light scattering of co-eluting fractions. (a) Zimm plot for linear PS (L-PS, $R_g/R_h \approx 1.42$). (b) Berry plot for hyperbranched PS (H-$PS_{1/650}$, $R_g/R_h \approx 1.08$). Both fractions exhibit similar $R_h \approx 27$ nm. Note: Due to unknown absolute concentrations of SEC fractions, a default nominal concentration of C = 1 mg/mL was used for data processing, which does not affect the robustness of the topology-dependent shape factor (R_g/R_h).

4) **Question:** Figure 7b is unclear, especially the cartoons and arrows, and D_f is undefined.

Reply: We sincerely thank you for pointing out these omissions. The cartoons, arrows, and D_f have been explicitly defined to ensure the clarity and rigor of our computational and theoretical discussions. We have addressed these points carefully in the revised manuscript:

a) Clarification on the cartoons and arrows

We have expanded the description in the main text and figure caption to explicitly define the visual elements in **Figure 7b**. The cartoons visually illustrate the structural transition and spatial densification from highly flexible linear chains to more compact hyperbranched polymers, ultimately approaching an idealized solid hard sphere. Crucially, the dashed horizontal arrows denote the dynamic amplitude of conformational fluctuations (i.e., the span of accessible states along the size axis, R). As now clarified in the text, the wide-spanning arrows for linear chains reflect a broad continuous distribution of loose conformations. Conversely, the markedly shorter arrows for hyperbranched chains represent scenarios where the conformational boundaries are severely restricted by the dense hyperbranched topological structure.

b) Definition and Significance of D_f

We apologize for the lack of definition. D_f stands for the Fractal Dimension, a fundamental parameter in polymer physics used to characterize the spatial compactness and mass distribution of polymer architectures. It relates the polymer's mass (M) to its size (R) via the scaling law $M \sim R^{D_f}$. In **Figure 7b**, D_f is used to illustrate the structural evolution from flexible coils to rigid spheres:

- For flexible linear polymers in a good solvent, the structure is relatively loose, corresponding to $D_f \sim 1.7$ (since $R_g \sim M^{0.6}$).
- For a hard sphere, the mass fills the 3D space uniformly, yielding $D_f = 3.0$.
- For hyperbranched polymers, the value falls in between (e.g., $D_f \sim 2.2$).

The significance of D_f in our context is to visually and physically demonstrate that hyperbranched chains are much denser and more sphere-like than linear chains. This architectural compactness explains why their interception behavior is largely governed by topology rather than temperature-induced swelling, distinguishing them from linear coils.

c) Revisions in the Manuscript

We have revised the description and analysis of Figure 7 (**page 21 line 16-19**) of the manuscript as follows："This structural densification is quantitatively evaluated by the fractal dimension (D_f, $M \sim R^{Df}$). While linear chains adopt swollen random coils (D_f = 1.7), hyperbranched polymers exhibit more compact, space-filling architectures (D_f = 2.2), approaching the theoretical limit of a non-fluctuating solid hard sphere (D_f = 3.0)."

5) **Question:** Please double check if the abscissa of Figure 3d is labeled correct.

Reply: We thank you for your careful observation and for pointing out this oversight. The abscissa (x-axis) of **Figure 3d** was inadvertently reversed during the figure preparation process. We sincerely apologize for this careless error. The x-axis label has been corrected in the revised **Figure 3d** to accurately reflect the data, as shown below (**Figure R7**).

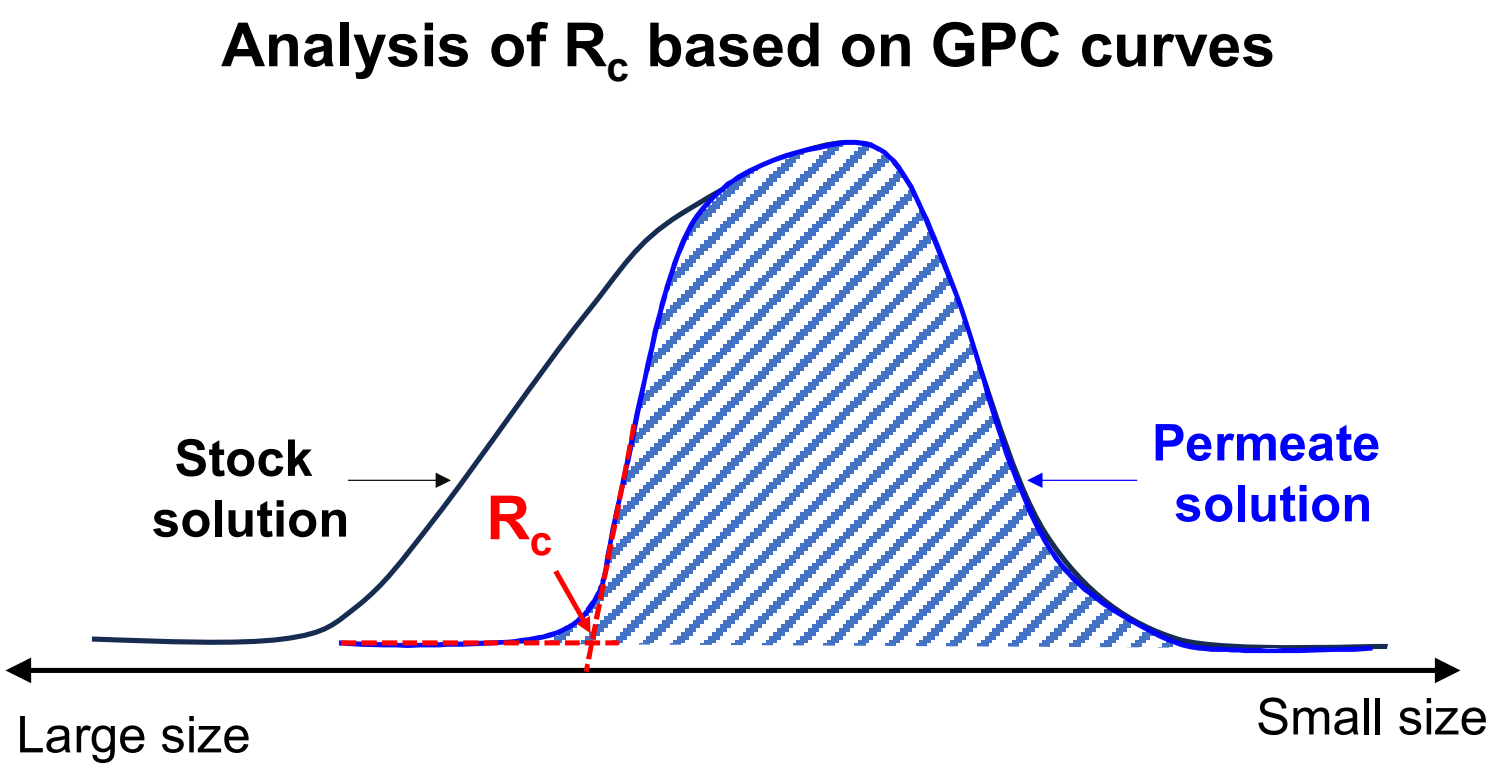

Figure R7. The retention size is obtained based on the molecular information of the original solution and the permeate solution through chromatographic analysis.

6) **Question:** Temperature data: The temperature-dependent experiments performed by the authors in the θ solvent for polystyrene clearly demonstrate the temperature insensitivity of the interception behavior of hyperbranched polymers. However, Figure 6c currently only presents SEC curves of hyperbranched polystyrene at 40 °C and 45 °C. More measurements at different temperatures need to be provided to validate the universality of temperature insensitivity in a broader temperature range.

Reply: We sincerely thank you for this excellent and rigorous suggestion. We fully agree that demonstrating the interception behavior over a broader temperature range—especially spanning across the classical θ-temperature of the polystyrene/cyclohexane system (~ 34.5 °C)—is crucial for validating the temperature insensitivity of hyperbranched polymers.

In response to your comment, we have performed additional temperature-dependent interception experiments for the hyperbranched polystyrene (H-PS) in cyclohexane at 30 °C and 35 °C. The newly updated **Figure 6c** now presents a comprehensive set of SEC permeate curves across a 15 °C span (30, 35, 40, and 45 °C). Note that a slight shift in curve shape and retention time may exist due to the potential change of column efficiency from time. The expanded dataset reveals a physically precise and highly consistent picture:

- At 30 °C (a poor solvent condition below the θ-temperature), we observe a slight increase in permeation. This is physically reasonable, as the polymer chains undergo a minor conformational collapse, slightly reducing their hydrodynamic size and facilitating passage through the nanopores.
- In sharp contrast, from 35 °C to 45 °C (spanning near and above the θ-temperature), the SEC curves

of the permeates overlap almost perfectly, showing much weaker temperature dependence.

This nuanced behavior robustly validates our proposed mechanism. Unlike highly flexible linear chains, which undergo dramatic coil-to-globule transitions and significant volume expansions driven by thermal solvent quality variations, the dense topological constraints and enhanced intrachain excluded volume of the hyperbranched architecture strongly suppress temperature-induced swelling in the good solvent regime (35-45 °C). While extreme poor-solvent conditions (30 °C) can induce a minor global compaction, the overall critical interception size of H-PS is predominantly dictated by its invariant branching topology rather than thermal-responsive solvent interactions. We have updated **Figure 6** in the revised manuscript to include these new data points and have explicitly discussed this nuanced temperature response. The updated figure was shown below (**Figure R8**).

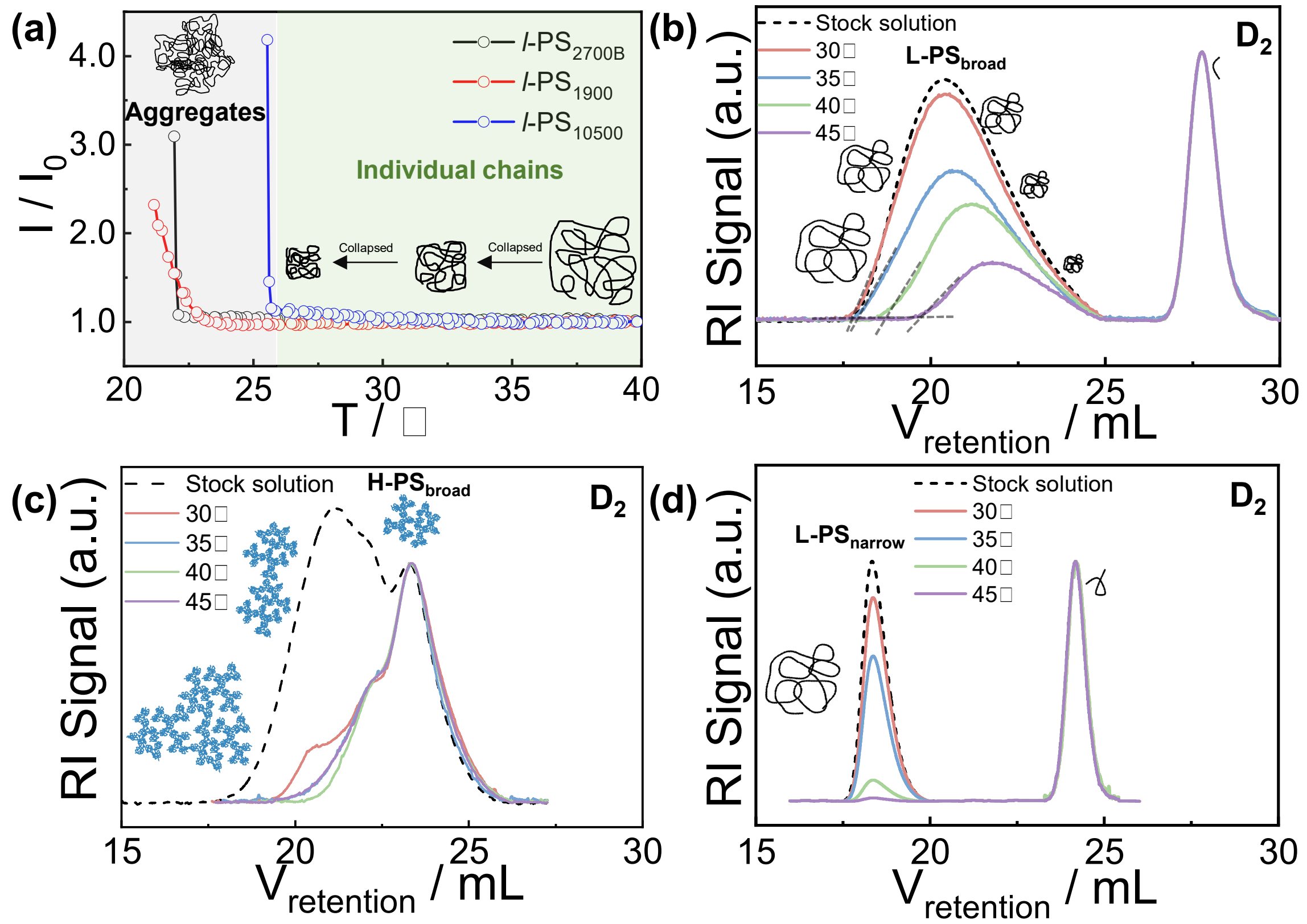

Figure R8. Temperature Dependence of Interception Behavior for Linear and Hyperbranched Polystyrene. (a) Static light scattering measurements of temperature-dependent relative scattering intensity for linear polystyrenes in cyclohexane. (b) Temperature-dependent interception profiles of broadly dispersed linear PS in cyclohexane. (c) Temperature-dependent interception profiles of broadly dispersed hyperbranched PS in cyclohexane. (d) Temperature-dependent interception profiles of narrowly dispersed linear PS in cyclohexane. All measured using a membrane with pore diameter D_2.

Revisions in the Manuscript

We have revised the description and analysis of Figure 6 (**page 18 line 8-19**) of the manuscript as follows：

“**Figures 6b and 6c** further illustrate the distinct temperature dependence of interception between linear and hyperbranched chains. Ultra-low molecular weight components were included as internal standards for concentration correction. … Within this wide good-solvent regime, the corresponding critical interception sizes remained nearly constant at approximately 20-23 nm, demonstrating a strong topological suppression of thermal swelling.”

7) Please remove one of the two commas on the first line of page 15.

Reply: We thank you for the careful reading and pointing out this typo. The redundant comma on the first line of page 15 has been removed in the revised manuscript.

<u>To the comments of reviewers 3</u>:

The authors submitted a manuscript the effect of (hyper-)branching of polystyrene (PS) polymers on the transport through nanopores. They observe a distinct power-law dependency between the critical interception size and the branch density. Though the work seems sound and it can be of interest to the polymer physics community, I think the is not of interest to the more generic audience of Nature Communications. Also, the novelty of the work is too minor compared to existing work - by the authors and by other authors - on the effect of PS polymer branching on the flow through nanopores. See for example, https://doi.org/10.1021/ma301468z, https://doi.org/10.1039/C0PY00361A. Therefore, I do not recommend this work to be considered for Nature Communications. Instead, I recommend that the authors submit the work to a specialized journal such as ACS Macromolecules.

Reply: We sincerely thank you for acknowledging that our work is sound and of interest to the polymer physics community. **However, we respectfully disagree with the assessment that the novelty is minor compared to previous work, and that the manuscript lacks broader interest for Nature Communications.**

a) A fundamentally different physical regime

The two papers cited by the reviewer [*Macromolecules* **2012**, *45*, 7583; *Polym. Chem.* **2011**, *2*, 1071] both address the **strong-compression limit**, where high-velocity flow induces substantial chain deformation (coil-stretch transition). In that regime, the critical parameter is the **flow rate** needed to pull a chain through a pore to realize chain deformation. In stark contrast, our work focuses on the **weak-compression limit**, where diffusion dominates and chain deformation is negligible. In this regime, the central parameter is the **critical interception size** $R_{h,c}$—an entropic bottleneck that determines whether a chain can passively enter a pore under near-equilibrium conditions. These two regimes are physically distinct and have been recognized as such since de Gennes. The weak-compression limit, despite its direct relevance to biological filtration and size-exclusion chromatography, has remained largely unexplored experimentally for branched architectures. Our study provides the first systematic investigation of this regime for hyperbranched polymers, revealing a topology-dependent scaling that is absent in the strong-compression literature. The two bodies of work are therefore complementary, not overlapping, and our contribution opens a new direction rather than incrementally extending previous findings.

b) Broader impact beyond polymer physics

The reviewer suggests that our work is only of interest to specialists. We respectfully argue otherwise. The transport of hyperbranched polymers through nanopores under diffusion-dominated conditions is directly relevant to **biological systems**. Glycogen and starch—two of the most abundant biopolymers in nature—are hyperbranched. Their translocation through glomerular filtration barriers, nuclear pore complexes, and other biological channels is implicated in metabolic diseases (diabetes, obesity) and cellular trafficking. By establishing how branching density quantitatively controls the critical interception size, our study provides a physical framework that can guide biomedical research on these systems. Moreover, our finding that the interception size follows a clear power law in branching density offers a design principle for advanced **membrane separation technologies**—e.g., separating polydisperse polymers by topology rather than by size, which has long been a goal in the field. These cross-disciplinary implications are explicitly stated in our introduction and conclusion, and they align well with the broad readership of Nature Communications.

We have revised the manuscript to more clearly highlight the distinction between the strong- and weak-compression regimes (Scheme 1 and Introduction) and to emphasize the biological and

technological motivations. We hope that with these clarifications, you will reconsider the novelty and general interest of our work.

To the comments of reviewer 1:

The authors have provided substantial evidence and made substantial changes to the manuscript. The comments of this reviewer have been addressed. The authors have also adequately addressed the comments of Reviewer 4.

Author reply: We sincerely thank the reviewer for the positive assessment of our revised manuscript. We greatly appreciate the reviewer's careful evaluation and recognition that the additional evidence and substantial revisions have addressed the concerns raised. The reviewer's constructive comments throughout the review process have been invaluable in improving the clarity and overall quality of our work.

To the comments of reviewer 2:

I co-reviewed this manuscript with one of the reviewers who provided the listed reports. This is part of the Nature Communications initiative to facilitate training in peer review and to provide appropriate recognition for Early Career Researchers who co-review manuscripts.

Author reply: We sincerely thank the reviewer for their time and contribution to the evaluation of our manuscript. We appreciate their participation in the Nature Communications co-reviewing initiative and their valuable contribution to the peer-review process.

In this work, Wang et al used hyperbranched polystyrene, monodisperse AAO nanopores, all-atom MD simulations, and theoretical modeling to focus how branching topology controls the critical interception size of polymers in nanopores under the weak-compression limit. They identified a unique scaling relation $R_{h,\,c} \sim \rho^{-1/2}$ for hyperbranched polymers, distinct from linear chains. The present work is novel so that it is suitable to be published on NC. Nevertheless, I have a few comments which I list below in no particular order:

(1) 'Figure 1'simplification: Reduce subplots by moving repetitive curves to the SI; keep only representative data for better readability.

(2) SEC validation: It should be noted that SEC only provides apparent molecular weight for nonlinear polymers, and the hydrodynamic radius is converted using universal scaling laws established from linear standards. For hyperbranched polymers with significantly different architectures, especially high-branching-density systems, the quantitative accuracy of this conversion method requires further support. I recommend to provide dynamic light scattering measurements of key samples to verify the reliability of Rh distributions converted from SEC using hydrodynamic radius, thereby improving the rigor of key data.

(3) I recommend to also use static light scattering to measure Rg and dynamic light scattering to measure Rh, so that the shape factor Rg/Rh can be obtained. And the shape factor Rg/Rh is a perfect parameter to quantify the degree of branching.

(4) Figure 7b is unclear, especially the cartoons and arrows, and Df is undefined.

(5) Please double check if the abscissa of Figure 3d is labeled correct.

(6) Temperature data: The temperature-dependent experiments performed by the authors in the θ solvent for polystyrene clearly demonstrate the temperature insensitivity of the interception behavior of hyperbranched polymers. However, Figure 6c currently only presents SEC curves of hyperbranched polystyrene at 40 °C and 45 °C. More measurements at different temperatures need to be provided to validate the universality of temperature insensitivity in a broader temperature range.

(7) Please remove one of the two commas on the first line of page 15.
